# Visualizing nanoscale charge flows in multi-dimensional WSe_2_/GaAs vertical diodes

**DOI:** 10.1038/s43246-026-01234-6

**Published:** 2026-06-13

**Authors:** Michele Zendrini, Claire Blaga, Matilde Cantele, Kamil Artur Wodzislawski, Alok Rudra, Mitali Banerjee, Anna Fontcuberta i Morral, Valerio Piazza

**Affiliations:** 1 Laboratory of Semiconductor Materials, Institute of Materials, Ecole Polytechnique Fédérale de Lausanne (EPFL), Lausanne, Switzerland; 2 Epitaxy Core Facility, Ecole Polytechnique Fédérale de Lausanne (EPFL), Lausanne, Switzerland; 3 Laboratory of Quantum Physics, Topology and Correlations, Institute of Physics, Ecole Polytechnique Fédérale de Lausanne (EPFL), Lausanne, Switzerland; 4 Institute of Physics, Ecole Polytechnique Fédérale de Lausanne (EPFL), Lausanne, Switzerland

**Keywords:** Two-dimensional materials, Electrical and electronic engineering

## Abstract

The unique properties of transition metal dichalcogenides provide a versatile platform for scalable, room-temperature quantum devices. Their future commercialization will inevitably depend on the development of heterostructures facilitating charge injection into pre-defined areas of 2D components. We showcase here an innovative approach to accurately characterize electrically active nanoscale regions in multidimensional heterostructures made of WSe₂ layers and GaAs, thin films and in-plane one-dimensional nanostructures. The rectifying behavior of these stacks is visualized through electron-beam induced current mapping with unprecedented spatial resolution. These electrical measurements reveal local diffusive and drift charge flows through the diode. Notably, we observe a significant reduction in the effective carrier concentration in WSe_2_ layers thinner than 15 nm. 2D/1D ensembles exhibit in-plane confinement of the space charge region as narrow as 140 nm. This level of control over dimensionality, position, and size opens exciting avenues to achieve tunable quantum sources, one step closer to 2D-based quantum devices.

## Abstract (web summary)

mlTransition metal dichalcogenides offer a promising foundation for scalable quantum devices, yet their commercialization hinges on precise charge injection in 2D components. Here, the authors employ electron-beam induced current mapping to characterize nanoscale regions in WSe₂/GaAs heterostructures, revealing insights that advance tunable quantum source development in 2D-based devices.

## Introduction

2D semiconductors are prime candidates for next-generation nanoelectronics and optoelectronics^1,2^. Transition metal dichalcogenides (TMDs) hosting tunable localized states are exceptional quantum platforms^3^. At the same time, the increasing number of reports on 2D-based devices such as transistors, photodetectors and emitters^4^ sparks the hope to integrate them in commercial quantum technologies in the near future^5^. Still, control over quantum properties requires the ability to access key mechanisms at the nanoscale such as exciton funneling in strained regions^6–8^. Straintronic emitters require highly localized tensile strain to funnel excitons and trigger efficient radiative recombination. This strategy presents undoubted advantages such as deterministic positioning of emitters, industrial scalability and compatibility with well-established cleanroom processes. Several works report quantum emission from TMD layers deformed at the nanoscale via nanomaterials, bubbles, and nanoindented surfaces^9–11^. However, these passive nanocomponents prevent solid-state injection to the 2D layers, with excess excitons only provided by optical pumping. While the majority of quantum sources are optically pumped, electrical injection brings clear benefits in terms of scalability and control^12^. Electrically pumped randomly distributed defect emitters in Gr/hBN/WSe_2_ 2D heterostructures were demonstrated long-ago^13–15^. AFM indentation has been used to introduce localized emitting sites in the stack^16^. Nonetheless, these processes do not ensure scalability and homogenous electrical injection.

**Fig. 1:**
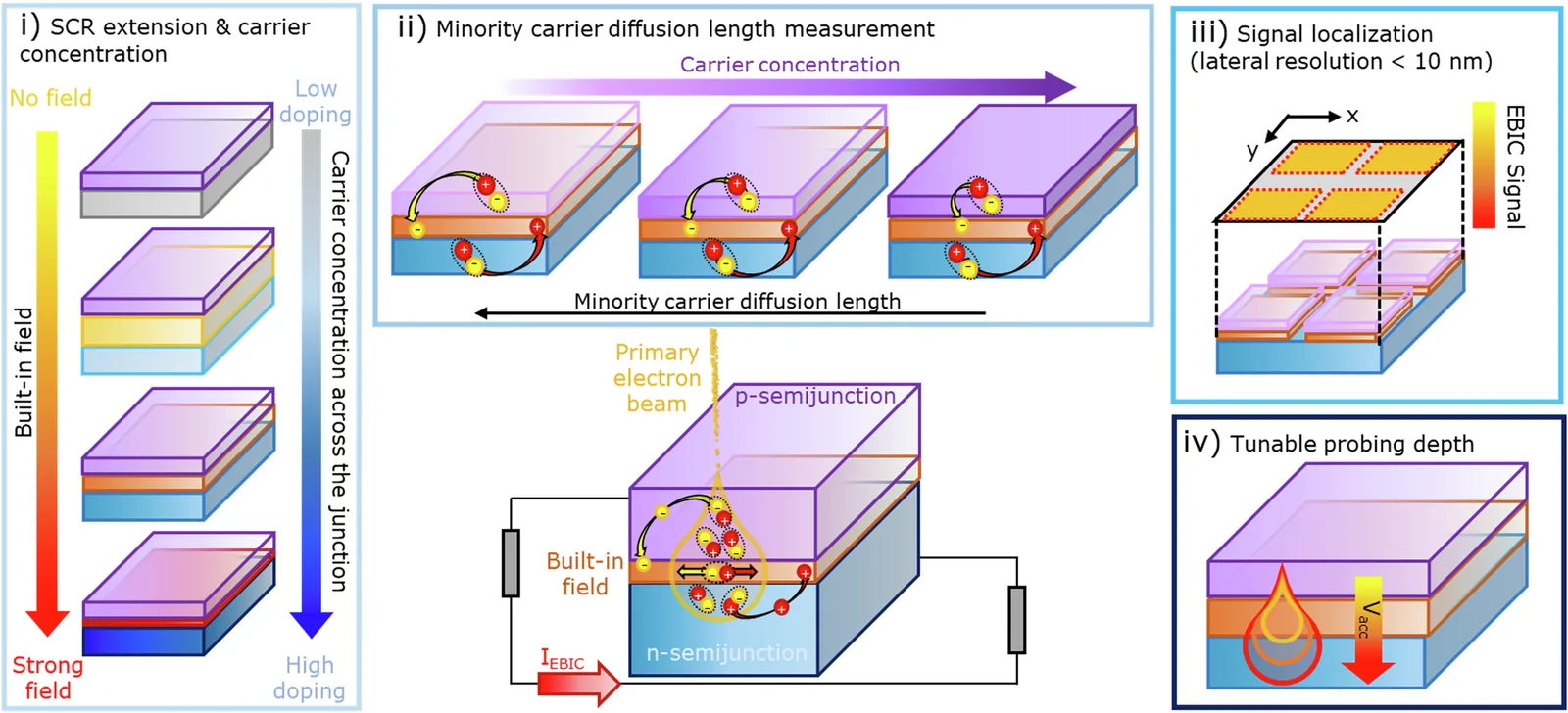
Working principle of EBIC mapping. A primary electron beam excites e^-^/h^+^ pairs due to inelastic scattering within a nanometric interaction volume. Excess charges in the space charge region drift apart while excited pairs in the vicinity of the junction diffuse. Both flows contribute to electron beam induced current. This technique is sensitive to the strength of the electric field and thus to the carrier concentration across the junction, which in turn defines the extension of the space charge region (SCR) (panel i). EBIC line profiles orthogonal to the rectifying interface offer a measure of the minority carrier diffusion length (panel ii). In case of localized junctions, maps display active and inactive regions with lateral resolution defined by the interaction volume (panel iii). By varying the energy of the primary beam, the penetration depth can be tuned thus accessing buried electric fields (panel iv).

In this work, we tackle two major limitations to large-scale production of TMD-based devices: localized electrical injection and nanoscale investigation of layered semiconductors. We propose to use III-V nanostructures to engineer carrier injection with nanometric accuracy in pre-defined positions. We focus on the built-in field at the heterointerface between 2D WSe_2_ layer on epitaxial GaAs, which form a vertical heterostructures. Selective area epitaxy tunes the geometry of nanostructures through the design of the mask opening^17–19^, thus enabling 0D, 1D and 2D heterointerfaces. Differently from their metal counterparts^20^, semiconductor heterojunctions can enable superior injection control through a diode architecture. The charge transfer across these heterojunctions is investigated via electron beam induced current mapping (EBIC). EBIC exploits a primary electron beam to locally excite excess charges, which are then collected as induced current under the effect of the built-in field^21,22^. Figure 1 presents the working principle of EBIC highlighting key relevant parameters such as depletion region extension^23^, charge concentration^24,25^ and minority carrier diffusion length^25,26^ (panels i and ii schematically represent planar junctions with built-in field and space charge region localized near the heterointerface). EBIC is also extremely effective to localize rectifying interfaces and associated charge transfer in-plane (panel iii) and in depth (panel iv) thanks to the reduced size of the interaction volume where the charge generation occurs^27^ granting a spatial resolution down to few tens of nanometers.

This work uses EBIC as a high-fidelity mapping technique for various WSe_2_/GaAs structures. First, we outline majority carrier type in WSe_2_ and unravel the intimate relationship between thickness and effective carrier concentration in 2D/3D heterostructures. Then we turn to 2D/1D systems demonstrating deterministically controlled in-plane confinement of the space charge region. EBIC maps finally showcase how the shape of nanocrystals and the thickness of the 2D component can be exploited to reduce the dimensionality of the heterointerface achieving an ordered array of quasi-0D region with size down to 150 nm.

## Results

### Electrical mapping of 2D/3D heterostructures

**Fig. 2:**
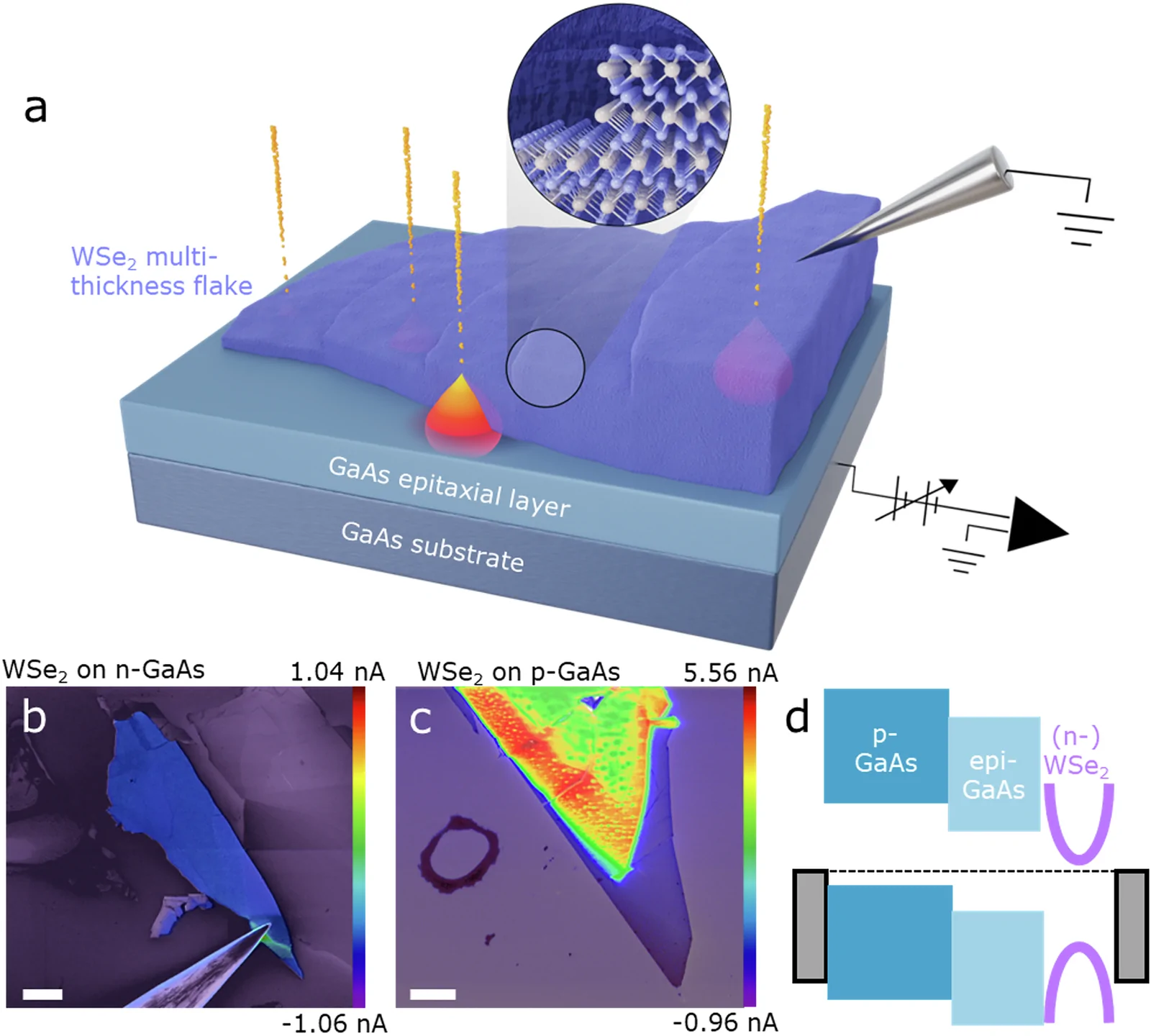
Electrical maps of exfoliated WSe_2_ on GaAs epitaxial thin films. **a** Schematic representation of 2D/3D WSe_2_/GaAs heterostructures. Interaction volumes are represented by droplets, probing at different depths depending on the flake thickness. Electrical maps acquired with bias V_b_* = *−1V overlaid with SEM images of WSe_2_ on epi-GaAs grown on (**b**) n- and (**c**) p- doped GaAs substrate. Substrates having a p-character creates a p-i-n heterojunction where a built-in field enables charge separation and collection, with current outlining the morphology of the flake and localized features (bubbles, wrinkles, defects). Map in panel c also reveals a variability in the current collected from different terraces of the flake. **d** Schematic band diagram illustrating the observed built-in field at the heterointerface. Scale bars* = *10 μm.

We start by investigating heterostructures containing a thin film of GaAs (nominally 50 nm thick) epitaxially grown on doped substrates (Supplementary Fig. 1). Multi-thickness WSe_2_ flakes are transferred on top, thus forming 2D/3D stacks as depicted in Fig. 2a. Upon electron beam exposure (with acceleration voltage of 2 keV and probe current ~0.4 nA), we measure the electrical response through in-situ tungsten tips mounted on nanomanipulators. The experimental setup enables to apply an external bias to the stack and to collect charges flowing through the heterostructures. The synchronization of current recording and electron beam position, together with the simultaneous detection of secondary electrons, returns spatially correlated SEM images and electrical maps. This lithography-free approach allows testing multiple flakes with relevant morphological features. We can thus outline the physical mechanism behind charge flow via spatial correlation between secondary electron emission (SE) and electrically active features. It is important to note that charge flows recorded in this configuration can have different origins such as potential difference between electrodes, excess charge generation, primary beam absorption and other possible mechanisms related to electron beam/semiconductor interactions^28–30^.

Figure 2b–c report the electrical maps of heterostructures containing Si- (n-type) and Zn-doped (p-type) GaAs substrates, respectively. First, the intensity of the current is significantly lower when WSe_2_ lies on n-doped substrates. Moreover, this current map shows little variation between edges, terraces and wrinkles of WSe_2_, or when exposing the metallic tip to the electron beam (on the bottom of Fig. 2b). These considerations indicate that this signal arises from collection of the primary beam and/or electron beam absorbed current^28^. While validating the efficient contact between the tip and the flake, this map outlines no charge transfer across the heterointerface. The electrical map acquired on WSe_2_/epi-GaAs/GaAs:Zn heterostructures (Fig. 2c) instead exhibits intense charge flow (manyfold the primary beam current) and reveal signal decrease around blisters, defects and wrinkles that naturally form at the heterointerface^31,32^. This behavior is consistent with excess charges collected by a built-in field at the WSe_2_/GaAs interface. Both measurements confirm the electron-rich nature of the exfoliated WSe_2_, consistently with other reports^33^ and demonstrates that WSe_2_/epi-GaAs/GaAs:Zn heterostructures act as vertical n-i-p diodes (Fig. 2d). EBIC maps under bias and in-situ IV measurements confirm current rectification in WSe_2_/GaAs samples on p-doped substrates (Supplementary Figs. 2 and 3). Measurements on 2D/3D heterostructures containing an epitaxial AlGaAs layer (WSe_2_/AlGaAs/GaAs:Zn) with %Al content ranging from 10% to 40% show the same behavior (Supplementary Fig. 4), demonstrating that the physical picture is independent of the band offset.

**Fig. 3:**
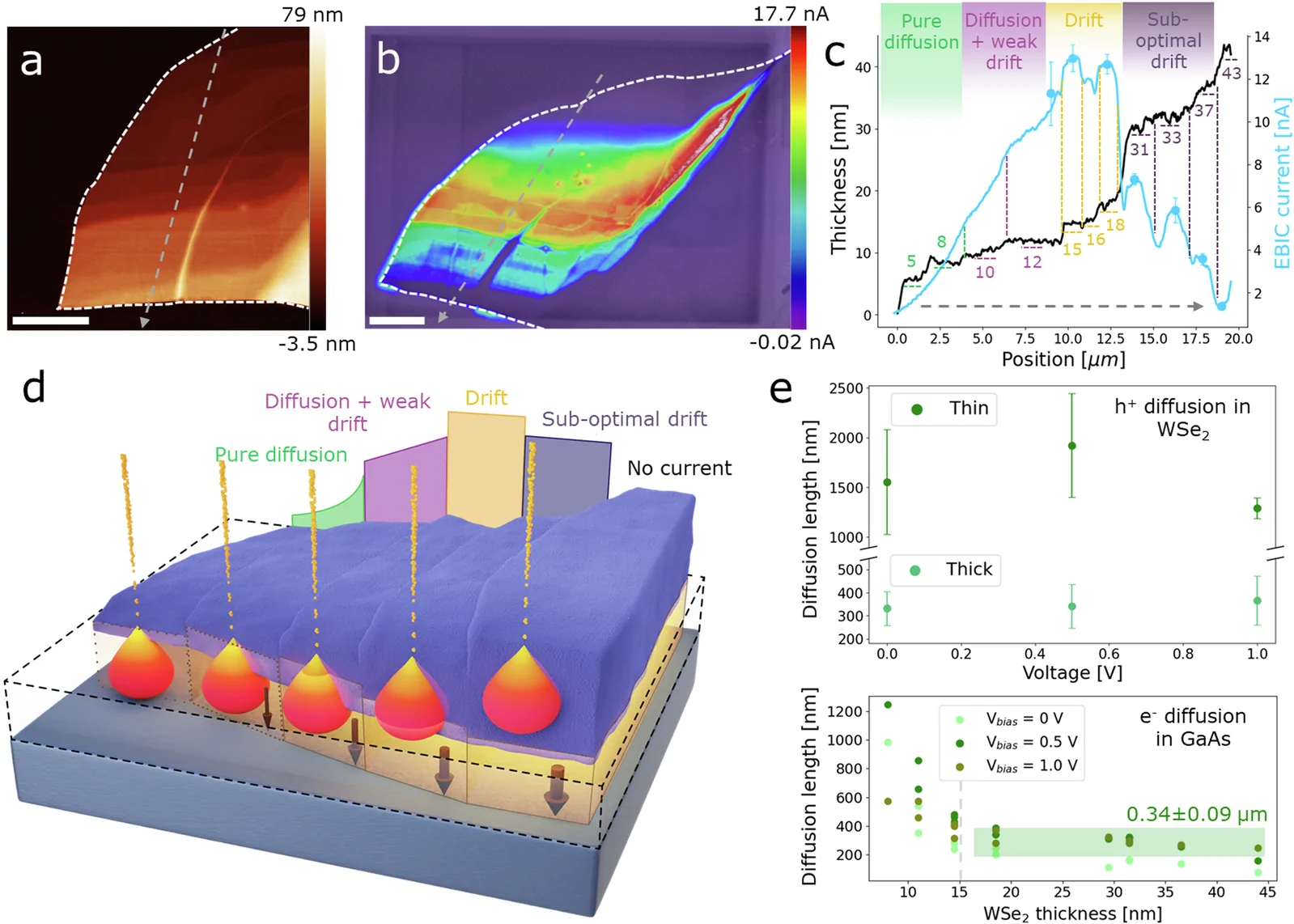
Thickness-dependent induced current. **a** AFM map of a WSe_2_ flake on epi-GaAs/GaAs:Zn substrate showing multiple atomically flat terraces. WSe_2_ thickness on the bottom is around 50 nm. **b** SEM/EBIC overlaid image of the flake reported in panel (a). EBIC map is acquired by applying 1 V reverse bias to enhance the built-in field. A dashed white line highlights the flake contour in panels (**a**) and (**b**). Scale bars represent 5 µm. **c** EBIC and AFM line profiles along the gray dashed arrows in panels (**a**) and (**b**). Each terrasse is delimited by dashed lines. The thickness of the terraces is indicated by the embedded values (in nm). The correlated plot highlights the current variation within terraces with thickness below 15 nm, signature of a diffusive transport. In contrast, current is constant on terraces thicker than the threshold thickness. Four regions can be defined where charge transport is dominated by pure diffusion (green), mixed diffusive/drift transport (violet box), pure drift (yellow and purple boxes, differing from optimal and suboptimal excitation). The color of dashed lines and thickness value reflect the color code used for the transport regimes. Points represent the current values averaged over the area of each terrace. Error bars represent the current variability on the terraces. **d** Comprehensive 3D schematic of carrier generation and collection mechanisms on terraces with varying thickness. The orange layer overlapping with the epi-GaAs and partially overlapping with WSe_2_ and with the GaAs substrate represents the space charge region. The arrows indicate the built-in field becoming less intense (thinner arrows) with decreasing thickness. Consistently with **c**, the panel describes (left to right) the diffusive transport in thin layers where the electron beam penetrates deep enough but the electric field vanishes (pure diffusion regime), intermediate regimes where current variation is sub-exponential (diffusion + weak drift), out-of-plane drift transport where current is constant on each terrace. In case of drift, we collect the highest signal (drift), a reduced signal (sub-optimal drift) or negligible signal (no current) depending on how much the interaction volume overlaps with the space charge region. **e** Minority carrier diffusion values in WSe_2_ at different applied reverse bias (top) and minority carrier diffusion values in GaAs as a function of flake thickness (bottom). Error bars in the top graph refer to fit deviation in multiple parallel profiles. The values in the bottom graphs are extracted from single profiles (at multiple reverse bias) across terraces with different thicknesses. Profiles crossing terraces thicker than 15 nm display a constant L_diff_ value estimated at around 0.3* ± *0.1 µm. Instead, the orthogonality condition collapses for profiles across thinner terraces due to the vanishing of the built-in field, and the 1D model cannot be applied. The fitting thus returns thickness-dependent L_diff_ values with no physical meaning.

Interestingly, these experiments reveal an unexpected variation of current with the WSe_2_ thickness. Figure 3a–b display respectively AFM and SEM/EBIC map of a WSe_2_ flake on epi-GaAs/GaAs:Zn substrate containing several terraces with thicknesses ranging from ~ 5 nm to ~ 50 nm. The presence of a gap at III-V/TMDs heterointerface due to hydrophilic adsorbates^34^, hinders the assessment of the real WSe_2_ thickness. Photoluminescence (PL) measurements excluded the presence of monolayers (Supplementary Fig. 5). Consistently with Fig. 2, the EBIC map in Fig. 3b shows that electrical signal is highly correlated to WSe_2_ thickness. In fact, the current collection appears negligible in the thinnest areas of the flake (top left of Fig. 3b). The signal gradually increases with thickness, peaks on terraces around 15 nm thick, and then decreases again on the thicker regions. The non-monotonous trend can be appreciated in line scans roughly perpendicular to the terraces’ elongation (Fig. 3c). The current decrease in thicker flakes is expected under these experimental conditions. Monte Carlo simulations indicate an electron beam penetration of ~ 40 nm (Supplementary Fig. 6), elegantly matching these experimental observations. The progressively increasing overlap between interaction volume and space charge region in the intrinsic epi-GaAs layer perfectly explains the electrical signal collected from 15–40 nm thick flakes. In this regime, the current collected purely depends on the thickness of the top layer. Therefore, we observe constant current values within terraces and a step-like variation with thickness.

Increasing the acceleration voltage of the primary beam allows to probe the heterostructure deeper. However, the increase of beam penetration depth is associated with interaction volume broadening which, in turn, affects the in-plane spatial resolution. Increasing acceleration voltage thus prevents accurate correlation with morphological features (see Supplementary Fig.7). For this reason, low acceleration voltage appears best suited to perform high fidelity micrograph correlation. Under these conditions, charge excitation and extracted current are more sensitive to the surface. Moreover, EBIC sensitivity is suboptimal and decreases with increasing thickness. We therefore optimized the probe current. In fact, the signal-to-noise ratio benefits from increasing the primary beam current. At the same time, aggressive exposure induces carbon deposition and beam induced damage^35^. The chosen probe current maximizes the signal-to-noise ratio while minimizing artifacts arising from electron beam exposure (Supplementary Fig. 8). These measurement conditions were found to be optimal to investigate thin terraces. Thin areas (thickness <15 nm) exhibit different characteristics. Here, the electrical signal varies significantly within each terrace. It is important to note a weak correlation with the terraces’ position, slowly vanishing when reaching the thinnest regions, where the current profile assumes an exponential shape. These elements—together with measurements with increasing electron beam energy (Supplementary Fig. 7)—indicate that the charge transport in thin flakes is dominated by in-plane diffusion. Diffusion tails in EBIC micrographs can be observed when the primary beam is perpendicular to the built-in field vector^27,36^, which is not the case for vertical diodes. The planar symmetry of the stack prevents visualization of the out-of-plane diffusion transport and should hinder lateral diffusion (as excited charges are expected to flow towards the heterointerface). The identification of in-plane diffusive transport in this system, therefore, implies a significant weakening of the vertical built-in field in thin WSe_2_ terraces. Non-idealities from sample preparation (contaminants, oxides, van-der-Waals gap) could account for this effect but it appears unlikely due to the tight correlation with the flake thickness throughout the investigated samples. On the contrary, the decrease of $\,\frac{\partial {I}_{{EBIC}}}{\partial x}\,$ in thin flakes indicates that the thickness defines the electric field strength (the following section will demonstrate the same behavior across multiple samples). Altogether, these observations return a complex picture where the maximum electric field at the heterointerface and EBIC probe depth varies with WSe_2_ thickness (Fig. 3d).

In-plane diffusive transport can be modeled through simple 1D Poisson equations^37^ and minority carrier diffusion length (L_diff_)^27^ can be extracted in several areas (Supplementary Fig. 9). Figure 3e reports hole L_diff_ in thick ( > 50 nm) and thin ( < 10 nm) WSe_2_ regions, which is found to vary from 0.35 ± 0.18 µm in thick areas and around ~2 µm in thin areas (details in Supplementary Fig. 10). In the latter case, contribution from the GaAs substrate must be excluded. Electron diffusion lengths in pure GaAs as a function of the WSe_2_ thickness (Fig. 3e) confirms the presence of the two transport regimes. Diffusion length associated to terraces is evidently dependent on the WSe_2_ thickness. This is extremely surprising as the minority carrier diffusion length in a semi-junction is not affected by the properties of the other semi-junction. This apparent WSe_2_ thickness-dependent electron diffusion behavior can be explained, once again by the absence (or extreme weakening) of the built-in field for thin terraces. Charges thus diffusion is therefore not perpendicular to the edge flake and the extracted values lose their physical meaning. In contrast, we observe a constant L_diff_ value - as expected - for terraces thicker than 15 nm, estimated to be around 0.29 ± 0.1 µm. The built-in field quenching and the diffusion length estimates suggest a reduction of the electron concentration in WSe_2_ with decreasing thickness. This hypothesis contrasts with theoretical and experimental works, which suggest an opposite trend^38,39^. However, airborne contaminants and molecules have been reported to have a thickness-dependent impact on carrier concentration^33^ consistent with our work. Measurements performed on hBN-encapsulated samples strongly support the link between the observed vanishing electric field and contaminants on the top surface (Supplementary Fig.11). The observation of drift and diffusive transport on different areas of the same 2D component has far-reaching implications for two reasons. First, charge flows in the two regimes occur along orthogonal directions (drift is out-of-plane, diffusion is in-plane) opening new perspective to design nanoscale circuits with customized carrier flows. Moreover, one can spatially separate 2D photonic (usually mono- or few-layers) and electronic (many-layers or bulk) regions by tailoring excess charge redistribution via thickness control, thus overcoming conventional trade-off between efficient light coupling and optimal current injection/collection.

### Electric field confinement in 2D/1D heterostructures

**Fig. 4:**
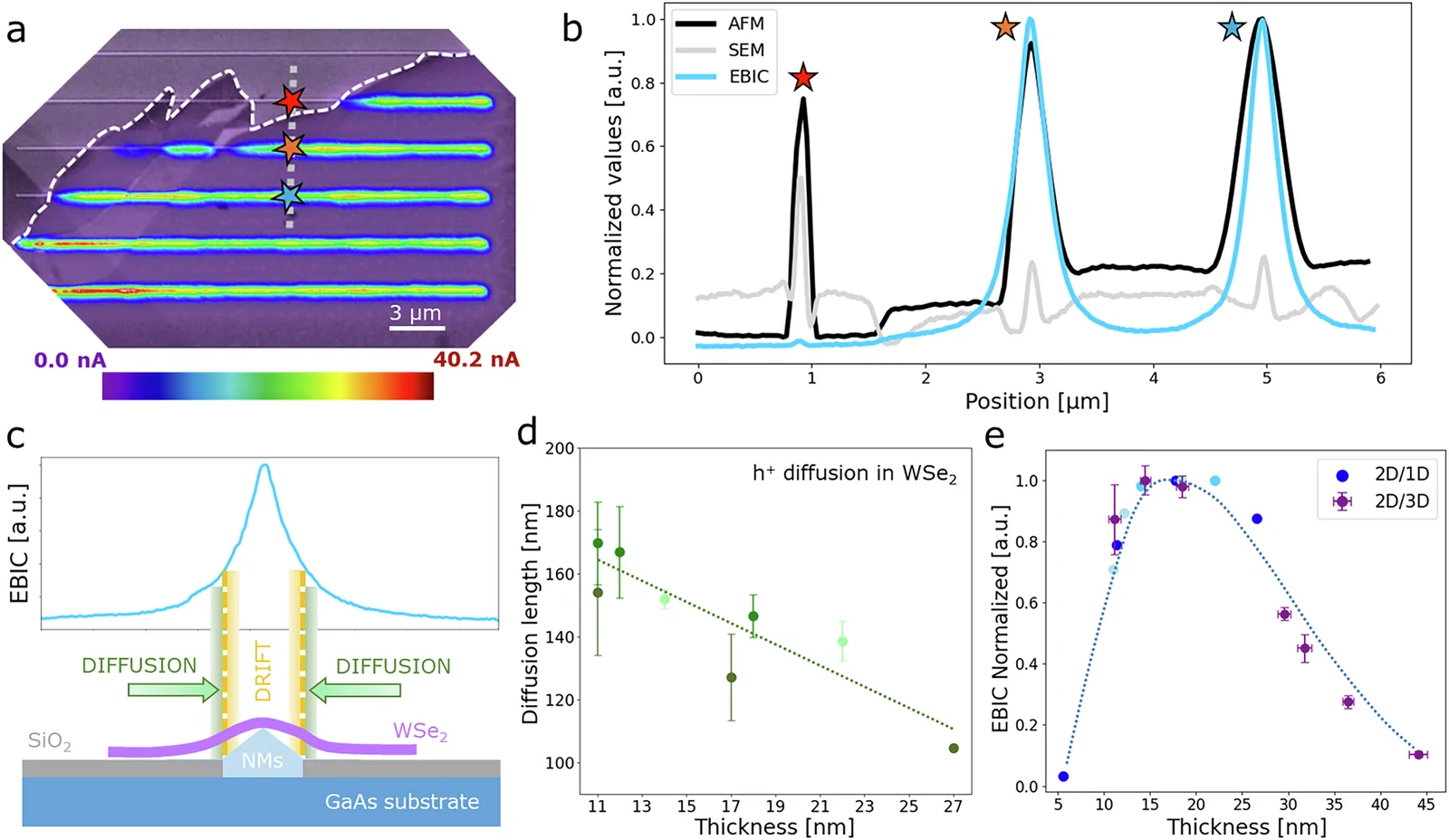
Electric field confinement in 2D/1D heterostructures. **a** Overlaid SEM/EBIC map (external bias: -2V) of a WSe_2_ flake on 1D GaAs NMs/GaAs:Zn substrate. The dashed white line indicates the edge of the WSe_2_ flake. **b** AFM/SEM/EBIC profiles from the line indicated by the gray dashed line in panel (**a**), with stars indicating the central position of the 1D nanostructures and highlighting clear morphological and electrical correspondence at the WSe_2_/GaAs-NMs interface (orange and yellow stars). Reasonably, bare NMs do not result in any current collection (red star). **c** Cross-sectional schematic of 2D/1D heterostructures and corresponding EBIC profile. The localized interface allows distinguishing areas dominated by out-of-plane drift from regions dominated by in-plane diffusion. **d** Minority carrier diffusion values in WSe_2_ as a function of flake thickness. Error bars refer to fit deviation in multiple parallel profiles. **e** Normalized EBIC intensity from different samples (2D/3D in purple and 2D/1D in blue shades), demonstrating current drop for very thin (<15nm) and very thick (~40nm)flakes, regardless of the substrate dimensionality. EBIC maps have been acquired under electron beam with 3 keV acceleration voltage and~0.4 nA probe current).

In-plane GaAs nanostructures offer an alternative path to achieve simultaneous vertical drift and lateral carrier diffusion. Here we exploit selective area epitaxy to grow ordered arrays of in-plane 1D GaAs nanomembranes (NMs) along <010> direction, surrounded by a SiO_2_ mask as done in previous works^17–19^. GaAs nanomembranes grow with perfect atomic arrangement and atomically flat, well-defined facets (Supplementary Fig. 12). Transfer of WSe_2_ flakes on GaAs NMs leads to 2D/1D heterojunctions. We investigated multiple samples containing terraces with thickness ranging between 10 nm and 30 nm. Figure 4a shows the EBIC map of one sample containing three WSe_2_ terraces (11 nm, 17 nm and 26 nm as reported in Supplementary Fig. 14) where localized 1D heterojunctions can be clearly visualized. The line scans in Fig. 4b highlight deterministic positioning of the electric field. Bare GaAs NMs (red star) display narrow SEM and AFM profiles, consistent with the <101> facets, and exhibit negligible electrical signal. WSe_2_/GaAs NMs (yellow and orange stars) instead can be identified by a weak SEM signal due to the screening by the top flake and a wider AFM profile due to the imperfect 2D-on−1D conformation^34^. EBIC signal peaks at these locations and sharply goes to noise level in between structures, demonstrating effective in-plane confinement of the space charge region. Photoluminescence maps show consistent emission quenching at these locations (Supplementary Fig. 13). These findings remarkably demonstrate a tight correlation between the adhesion of WSe_2_ on GaAs and the carrier transport.

The lateral confinement allows splitting the current recorded perpendicularly to the NM elongation into pure drift – at the GaAs location – and pure diffusion – on the SiO_2_ mask (Fig. 4c). Contrarily to the 2D/3D case, these heterostructures offer direct access to hole diffusion in thin WSe_2_ without perturbation from neighboring electric fields (fitting in Supplementary Figs. 14, 15, and 16). Figure 4d shows a gradual decrease of the hole diffusion length in WSe_2_ with increasing thickness, supporting the previous findings. We find an average L_diff_ ~ 100 nm for flakes with thickness around 25–30 nm, which deviates from the value estimated in equivalent areas of the 2D/3D heterostructures (Fig. 3e, L_diff_ ~ 300 nm for 40 nm thickness). Two hypotheses can account for this reduction: remote phonon scattering from the SiO_2_ mask or the compressive state of WSe_2_ localized around the GaAs/SiO_2_ interface observed at the edges of nanomaterials in multi-dimensional systems^40^. Both effects are theoretically expected to decrease charge mobility in TMDs^41,42^. Electrically active defects or surface states in GaAs nanostructures may also perturb the equilibrium carrier concentration of WSe_2_. Such mechanisms would also affect the strength of the electric field. To verify this hypothesis, we plot the peak current value (normalized to account for variable contact resistance) versus WSe_2_ thickness as reported in Fig. 4e. The plot reveals a common trend for all the samples of this study where the EBIC current drops for WSe_2_ flakes thinner than 15 nm. This evidence rules out any detrimental effect due to the III-V crystals. Instead, it confirms that WSe_2_/GaAs heterostructures exhibit highly reproducible performance even at the sub-micron scale.

**Fig. 5:**
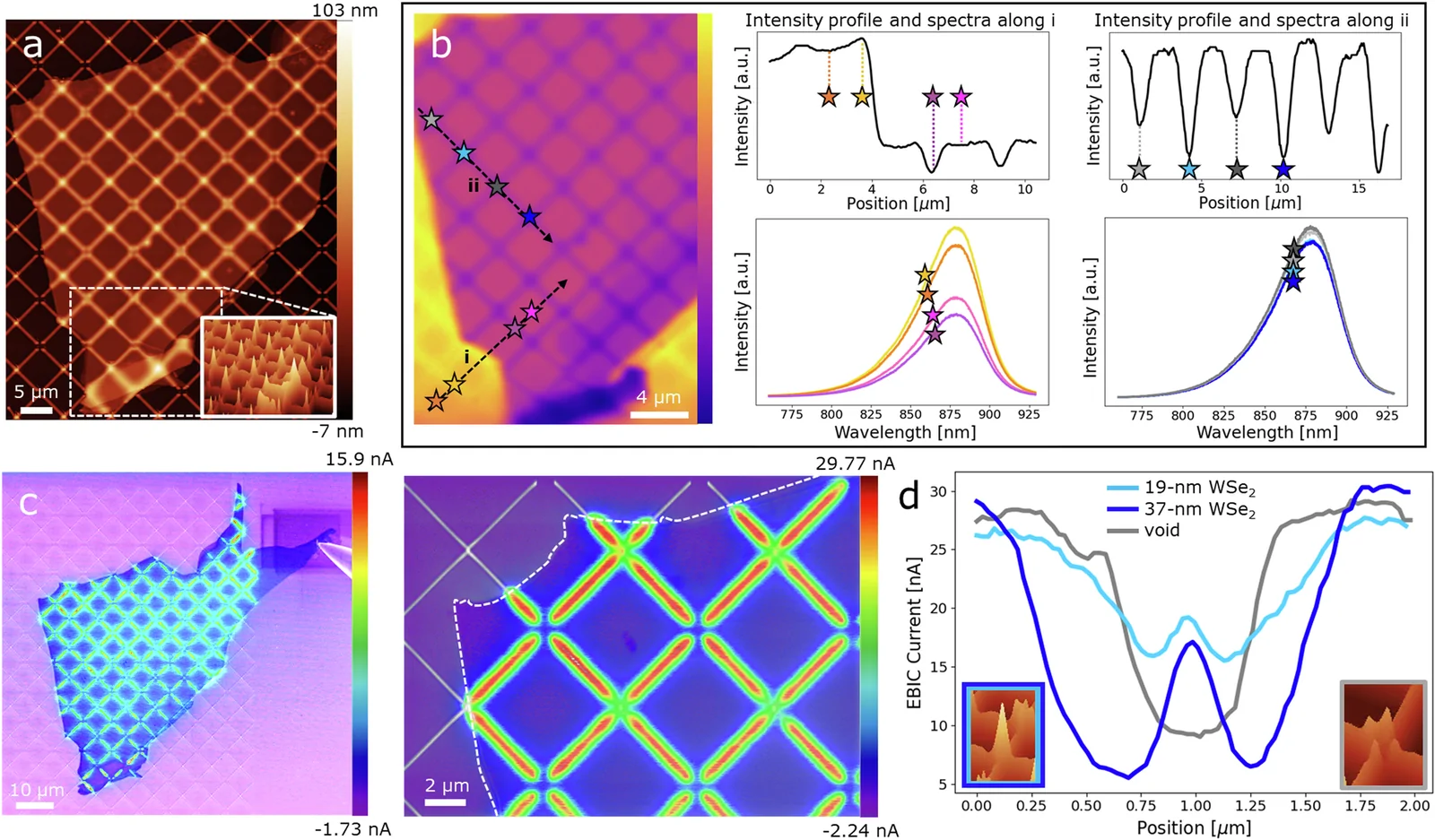
In-plane visualization of built-in field in 2D/1D nanonetworks. **a** AFM map of a WSe_2_ flake (thickness ranging from 10 nm to 40 nm) on GaAs NXs. The 3D inset showing a close-up highlights the presence of spikes at the intersections. **b** Room temperature PL of WSe_2_ on NXs. The map shows the luminescence from GaAs. Two intensity profiles and selected spectra are extracted along the GaAs elongation directions (i and ii arrows). Pixels along (i) offers access to four relevant locations, which are found spectrally equivalent (in order, left to right): SiO_2_/GaAs (orange star), GaAs NM-on-GaAs (yellow star) and equivalent positions covered with WSe_2_ (purple and pink stars, respectively). The intensity profile shows that emission of bare GaAs NMs is brighter than SiO_2_/GaAs while the reverse is true after transferring of WSe_2_, due to charge separation at the heterointerface. Pixels along (ii) pass through spikes and voids (blue and gray stars, respectively). Intensity profiles and spectra reflect the PL limitations to investigate these two non-equivalent locations, which appear hardly distinguishable due to low spatial resolution and competing mechanisms (laser screening and charge separation). **c** Low and high magnification SEM/EBIC maps of the WSe_2_/GaAs NXs. The close-up shows highly homogeneous current collection on the horizontal 1D structures and signal modulation at the spikes or voids location. **d** EBIC profiles across different NX intersections (void in gray, spike in blue palette). The profiles highlight current suppression at the void location and current localization at the spike location. Thick WSe_2_ leads to full isolation of the quasi-0D space charge region (dark blue). EBIC maps have been acquired under electron beam with 3 keV acceleration voltage and ~ 0.4 nA probe current).

Linear NMs offer a simple platform to describe in-plane confinement. Real devices may require interconnected networks with gaps and standing elements to exploit straintronic photonic mechanisms^9^. Figure 5a reports the AFM map of a WSe_2_ flake on a GaAs network, made of nano-crosses (NXs) grown along equivalent <001> and <010> directions, ensuring that all the outer facets belong to the same family^18^. The network consists of parallel and perpendicular linear nanostructures. Intersections resulted in localized spikes with average base diameter of 850 nm while disconnected areas appear as 530 nm wide voids (Supplementary Fig. 17). AFM data estimate WSe_2_ thicknesses varying from 10 to 40 nm. Consistently, room temperature micro-PL confirmed the absence of 1L-regions (Supplementary Fig. 18). GaAs band-edge luminescence mapping reveals quenching of the emission at the WSe_2_/GaAs interface. The absence of spectral differences across the sample confirms a reduced radiative efficiency due to charge separation (Fig. 5b). Further analysis by micro-PL is hindered by laser screening (Supplementary Figs. 5 and 13) preventing to investigate the impact of WSe_2_ thickness. Moreover, spikes and voids are hardly distinguishable due to charge separation and the micrometric spatial resolution of PL maps. EBIC instead achieves significantly higher spatial resolution. Figure 5c clearly shows the collection of charges exclusively from the heterointerface areas, revealing the position of spikes. Through external bias, the collected current on the elongated horizontal junctions can be stabilized with outstandingly low local variability (down to 17%, Supplementary Fig. 19).

Collected current systematically drops at the void position while spike locations exhibit a lower current decrease. In both cases, we expect a current decrease due to the detachment of the WSe_2_ from GaAs. In the void case, this is due to the absence of GaAs, thus the current decrease follows the discussed diffusion tail. We measure an average hole diffusion length across void location around 120 nm, consistent with linear nanostructures (Supplementary Fig. 20). For vertical spikes, the conformation of the WSe_2_ on GaAs plays a fundamental role in defining both the extent of the heterointerface and the strain distribution which may affect the diffusion length. Additionally, the presence of a central area of contact results in localized current, as shown in Fig. 5d. EBIC profiles at the spike location reveal the presence of current peaks at the central position. The combination of diffusion driven current drop and nearly pointwise heterointerface results in quasi-0D in-plane confinement of the built-in field, with an estimated extension down to 140 nm. Taller structures could improve the confinement but would also impact the strain distribution and could increase the chance of destructive events such as piercing^43^. Thicker regions instead appear to result in stronger confinement (blue curve of Fig. 5d), most likely due to the reduced conformity of more rigid flakes (Supplementary Fig. 21). Thickness therefore appears to play a significant role also to provide mechanical stability at the nanoscale.

### Conclusions

This work demonstrates innovative WSe₂/GaAs vertical heterostructures that enable highly precise in-plane electronic design at the nanoscale. The combination of the unique properties of layered 2D semiconductors with mature III-V processing techniques raises strong expectations for future optoelectronic and quantum applications.

The complex relationship between structural and functional properties in these ensembles is elucidated by correlative microscopy—atomic force microscopy for morphology, electron beam induced current for the first time applied to layered semiconductors to resolve rectifying features, and optical mapping for contextual analysis. Applying this approach to both 2D/3D and 2D/1D heterostructures, the study demonstrates how WSe₂ thickness critically influences charge carrier concentrations and built-in fields. Variations in the intensity of the electric signal and analysis of minority carrier diffusion lengths consistently point out to a progressive reduction of built-in field intensity below a critical thickness. This is attributed to a reduction in majority carrier concentration in thinner regions, likely driven by environmental factors like adsorbates.

Nanoscale mapping reveals that charge transport and in-plane electric field confinement can be precisely controlled at various length scales, even allowing to reduce WSe₂/GaAs interfaces to regions as small as 200 nm without compromising the overall functionality. These findings reveal enormous potential to downscale these heterostructures without loss of performance. Furthermore, by transferring WSe₂ onto a nano-network with varied morphologies, the research showcases simultaneous presence of quasi-0D and quasi−1D active regions in predefined location, demonstrating how heterointerface behavior and active regions can be deterministically confined and engineered at the nanometric level. In this framework, techniques such as micro-PL and EBIC play a key role in revealing localized charge separation, emission quenching, and the formation of potential injection sites as small as 140 nm, setting the stage for the development of sub-micron, position-controlled electronic and optoelectronic devices based on 2D materials. Altogether, this work underscores the potential for sophisticated, nanoscale electronic and quantum architectures leveraging 2D/3D and 2D/1D heterostructures with precise spatial engineering.

## Methods

### GaAs thin film growth via MOVPE

Zn-doped and Si-doped (100) GaAs wafers have been used to homoepitaxially grow GaAs thin films via metalorganic vapor phase epitaxy (MOVPE). The substrates are introduced into the MOVPE reactor (Aixtron AIX 200) where they undergo an initial annealing step of 2 min at ∼820 °C under arsine and nitrogen atmosphere. Growths are performed at 700 °C for 500 s, at a nominal planar growth rate of 0.1 nm/s, corresponding to 50 nm in thickness. AFM characterization of surface smoothness and SEM cross-sectional characterization of the thickness are presented in Supplementary Fig. 1.

### Substrate preparation and epitaxy of nanostructures

GaAs nanostructures were grown on Zn-doped (100) GaAs wafers via selective area MOVPE. To achieve selective growth, a 25 nm thick SiO_2_ mask is deposited by plasma-enhanced chemical vapor deposition. Afterward, the designed shapes are patterned by electron beam lithography (Vistec EBPG5000ES) on 20% diluted ZEP positive resist and cold developed with n-amyl acetate. The pattern is transferred to the mask by reactive ion etching with CHF_3_/SF_6_ chemistry in an SPTS/APS plasma etcher. Upon cleaning resist residuals with acetone and IPA sonication, the wafer is finally etched with diluted HF (1:39 wt% in H_2_O) to decrease the oxide surface roughness coming from reactive ion etching. The openings are elongated slits along equivalent [001] and [010] crystalline directions. As-prepared chips are introduced into the MOVPE reactor. The growth recipe begins with the same annealing step for thin film growth (2 min at ∼820 °C under AsH_3_ and N_2_). The growth step takes place at 650 °C with a TEGa flow rate of 66 sccm and an AsH_3_ flow rate of 40 sccm with nitrogen as a carrier gas. The molar V/III ratio is hence fixed to 3. These conditions correspond to an equivalent planar growth rate of ∼1 nm/min. The growth step duration is 90 s, providing fully developed pyramidal cross sections in the structures used for integration (Supplementary Fig. 10), consistently with previous investigations^18^. By patterning crossed, elongated slits in the mask, interconnected structures such as nano-crosses (NXs) grow naturally as singular elongated NMs along the two preferential growth directions. Spikes at the center are direct consequence of enhanced local growth rate due to the larger substrate area-to-mask ratio at the center of the X-shape^40^.

### WSe_2_ exfoliation and heterostructures fabrication

The TMD layer is mechanically exfoliated and dry-transferred by a visco-elastic transfer method on top of planar and nanostructured GaAs (100) substrates. The exfoliation is followed by optical microscopy recognition of few-layered flakes thanks to correlation of optical contrast with flake thickness. This is based on statistically estimated cross-reference between thickness and contrast with respect to the substrate via photoluminescence and AFM thickness identification. The transfer is performed through a PDMS strip attached to a laboratory glass slide. The glass slide is positioned upside down onto a three-axes transfer station with embedded optical microscope to deterministically position the flake on desired locations of the substrate. Once contact is obtained between flake and substrate, the substrate stage is heated up to 90 °C for 5 min to increase adhesion. Detachment of the PDMS strip from the substrate allows for final heterojunction creation by leaving the flake at the desired location.

### Atomic force microscopy

The morphological characterization of nanostructures and heterojunctions is performed by a Bruker Dimension Icon AFM in tapping mode with TESPA-V2 probes. These have a 15 μm long, Al-coated cantilever with nominal tip radius of 7 nm and nominal resonance frequency of 320 kHz.

### Electron beam induced current measurements

The electrical behavior of the heterojunctions and the charge transfer across the stack is investigated with a lithographic free approach. As-fabricated heterostructures are investigated via scanning electron microscopy in a Zeiss Gemini SEM 300 microscope operating in high vacuum (HV) mode. Secondary electron (SE) images are acquired with a Gemini II column in-lens SE detector at 3 keV electron beam energy with an injection current of 100 pA (as measured by a Faraday cup). Electron beam induced current (EBIC) measurements were performed on an Imina NANO platform compatible with the Zeiss electron microscope. Four miBots, each equipped with a 500 nm radius tungsten tip, were controlled individually via Precisio software. Piezoelectric motion ensured nanometer control over the electrical contacts to individual flakes. Electrical access to WSe_2_ is achieved by placing the nanomanipulator on thicker regions to ensure both mechanical stability and efficient in-plane charge collection. A conductive holder allows for electrical access to the substrate. This strategy aims at minimizing contaminations from common cleanroom processes (chemisorption, adsorption, defect formation^44^) on the native charge population. Additionally, the in-situ contacts induce site-specific removal of contaminants^45^ and enable to test multiple flakes laying on the GaAs surface under identical conditions. Measurements were performed at 2 and 3 keV acceleration voltage and injected current in the range of 0.4 nA (as measured by Faraday cup). Experiments were performed by applying an external bias ranging from a maximum of −3 V to +3 V through a Point Electronic amplifier controlled by a Digital Imaging scanning system.

### Electrical analysis

The one-dimensional steady state continuity equation for minority carriers can be written as: ${g}-R-\frac{1}{q}\frac{\partial J}{\partial x}=0$, where *g* is the generation rate, *R* is the recombination rate of minority carriers, and *q* is the elementary charge. At a fixed injection regime, in a homogeneous material, the generation and recombination rates can be treated as constants. In these conditions, the continuity equation admits an exponential solution for the diffusion current density as:

$$J=A\cdot {e}^{(-\frac{x}{{L}_{{diff}}})}$$

where *J* is the current density, *A* is an integration constant and *L*_*diff*_ is the minority carrier diffusion length. Fitting the experimental data, we extract the minority carrier diffusion length of the individual semi-junctions from 1D induced current profiles^27^.

In this context, linear current profiles are extracted from EBIC maps to obtain quantitative analysis of the results. Current profiles for 2D/3D heterojunctions are thoroughly described in the main text, while current profiles across NMs are modeled in two components: a central region where the current collection is sustained by the out-of-plane drift and an outer region where in-plane diffusion dominates the signal. An in-bound uncertainty region accounts for the imperfect conformation of WSe_2_ on the GaAs NMs. Nevertheless, signal collected from WSe_2_/SiO_2_ regions around GaAs NMs can be undoubtedly attributed to minority carrier diffusion. Numerical exponential fitting of EBIC profiles acquired over multiple samples and NMs locations enabled a statistically robust analysis of the minority carrier diffusion length (details in Supplementary Figs. 14, 15 and 16).

### Photoluminescence mapping

The optical behavior of the samples was probed with photoluminescence mapping to give additional insights on the EBIC measurements. These measurements are done by scanning a defined area of the sample with the excitation source, in this case, a 532 nm continuous wave solid state laser from Coherent, with the help of a 63x objective mounted in a piezo actuator. In each point of the scanned area, the light emitted by the sample is sent to an Andor Shamrock 500i spectrometer, onto a 300 l/mm grating. The wavelength resolved emission spectra are captured by an iDus CCD. All PL measurements are taken with an excitation power of 40 µW on the sample and an acquisition time of 1 s for each spectrum. The size of each pixel in the PL maps is 0.1 × 0.1 µm^2^.

## Supplementary information

Transparent Peer Review file

Supplementary Information
