# Supplementary material for: Visualizing nanoscale charge flows in multi-dimensional WSe2/GaAs vertical diodes
Source: Commun Mater. 2026 Jun 13;7(1):294. doi: 10.1038/s43246-026-01234-6 (PMC13727687; doi:10.1038/s43246-026-01234-6)
Supplement: Supplementary file 1 [file 43246_2026_1234_MOESM1_ESM.pdf]

nature portfolio

Peer Review File

Visualizing Nanoscale Charge Flows in Multi-Dimensional WSe2/GaAs Vertical Diodes

Corresponding Author: Dr Valerio Piazza

This file contains all reviewer reports in order by version, followed by all author rebuttals in order by version.

Version 0:

Reviewer comments:

Reviewer #1

(Remarks to the Author)
The manuscript reports nanoscale electrical characterization of WSe2/GaAs vertical heterostructures using electron beam induced current (EBIC) mapping. By integrating exfoliated WSe2 flakes with planar GaAs films and selectively grown GaAs nanomembranes, the authors visualize local charge collection, infer built-in field distributions, and extract minority carrier diffusion lengths. A key claim is a thickness-dependent reduction of effective carrier concentration in WSe2 below ~15 nm, leading to a transition from drift-dominated to diffusion-dominated transport. The work further demonstrates lateral confinement of space-charge regions down to ~140 nm using 1D and networked GaAs nanostructures.

The spatial visualization of confined electric fields and dimensional crossover in carrier transport is a useful experimental contribution. However, several of the underlying physical concepts invoked, such as thickness-dependent transport in WSe2 and carrier injection across 2D/III-V heterojunctions, are not new in themselves. The novelty primarily lies in nanoscale EBIC mapping of engineered hybrid-dimensional junctions, rather than in a fundamentally new heterostructure concept or in the direct demonstration of quantum optoelectronic functionality. The authors should clearly highlight the novelty of their study. My other concerns are given below, which the authors should address before the manuscript can be considered further.

1) It will be good to through light on the nature of the heterojunction. Is it a van der Waals-type junction, or is it more like a conventional p-n junction type? Does it depend strongly on thickness?
2) Is there any formation of depletion layer at the junction? If that is the case, then what is the extent of this layer?
3) What is the mechanism behind the crossover of transport behaviour exactly at 15 nm thickness?
4) The layered nature of WS2 will cause highly anisotropic transport when considered in the in-plane and out-of-plane directions. Can the author discuss what the effect of such confinement on in-plane transport is? Also, do the authors observe any crossover
5) The effect of strain is an important point which needs to be discussed in greater detail. The author can fabricate samples of the same width but different heights to determine how strain affects the observed behaviour.

Reviewer #2

(Remarks to the Author)
see attached file

Reviewer #3

(Remarks to the Author)
Overall Recommendation: Major Revision

This study introduces a high-resolution EBIC characterization approach for investigating multi-dimensional WSe_2/GaAs heterostructures, with significant potential to elucidate nanoscale charge transport mechanisms in semiconductor devices. While the methodology and key findings are innovative, several substantial issues require clarification and enhancement before the manuscript is suitable for publication.

Below are my specific recommendations for revision:

1. Deepen the discussion on the physical mechanism behind thickness-dependent built-in field weakening. The explanation for the marked reduction in built-in field in WSe_2 regions thinner than ~15 nm currently remains somewhat speculative (e.g., attributed mainly to adsorbates). To strengthen this conclusion, the authors should incorporate supporting literature or provide a simplified theoretical estimate, for instance, modeling the relationship between carrier concentration and thickness. Adding a concise paragraph in the Discussion summarizing possible mechanisms would greatly improve the manuscript's rigor.

2. Provide more detailed descriptions of experimental methods and key parameters. To allow better assessment of the accuracy and reproducibility of the EBIC results, it is essential to include crucial measurement parameters such as primary beam current, probe contact force/resistance, signal acquisition time, and signal-to-noise ratio. These details should be added either in the Methods section or in the Supplementary Information.

3. Include a discussion on the limitations of the techniques employed. A short subsection in the Discussion or Conclusion should address the inherent limitations of EBIC in characterizing 2D/3D heterostructures, for example, the trade-off between penetration depth and spatial resolution, surface sensitivity, and the influence of beam-induced effects. Additionally, a brief comment on how strain or interfacial defects introduced during sample transfer could affect the results would provide a more balanced perspective.

4. Improve consistency between figures, captions, and textual descriptions. Several inconsistencies were noted. For instance, the descriptions for Supplementary Figures 2 and 3 appear nearly identical and should be carefully verified and corrected. Furthermore, the explanation of Figure 3e in the main text should be clarified to better distinguish data points described as having "no physical meaning" from those with clear physical interpretations.

Version 1:

Reviewer comments:

Reviewer #1

(Remarks to the Author)
The authors have satisfactorily addressed most of my comments and modified the manuscript accordingly. The manuscript can now be accepted for publication.

Reviewer #2

(Remarks to the Author)
I think is good to go.

Reviewer #3

(Remarks to the Author)
The authors have addressed my concerns. Recommend acceptance at current version.

Open Access This Peer Review File is licensed under a Creative Commons Attribution 4.0 International License, which permits use, sharing, adaptation, distribution and reproduction in any medium or format, as long as you give appropriate credit to the original author(s) and the source, provide a link to the Creative Commons license, and indicate if changes were made.
In cases where reviewers are anonymous, credit should be given to 'Anonymous Referee' and the source.
The images or other third party material in this Peer Review File are included in the article's Creative Commons license, unless indicated otherwise in a credit line to the material. If material is not included in the article's Creative Commons license and your intended use is not permitted by statutory regulation or exceeds the permitted use, you will need to obtain permission directly from the copyright holder.
To view a copy of this license, visit https://creativecommons.org/licenses/by/4.0/

EPFL

Laboratory of Semiconductor Materials,
Institute of Materials, School of Engineering

Lausanne, April 28th 2026

Communications Materials

Dear Editor,

We thank the reviewers for the positive comments on our manuscript. Please find below the detailed answers to the comments from them. We provide modifications and/or clarifications for each of the comments. All subsequent changes in the manuscript and SI are highlighted here in red and green.

With kind regards,

Reviewer #1 (Remarks to the Author):
The manuscript reports nanoscale electrical characterization of WSe_2/GaAs vertical heterostructures using electron beam induced current (EBIC) mapping. By integrating exfoliated WSe_2 flakes with planar GaAs films and selectively grown GaAs nanomembranes, the authors visualize local charge collection, infer built-in field distributions, and extract minority carrier diffusion lengths. A key claim is a thickness-dependent reduction of effective carrier concentration in WSe_2 below ~15 nm, leading to a transition from drift-dominated to diffusion-dominated transport. The work further demonstrates lateral confinement of space-charge regions down to ~140 nm using 1D and networked GaAs nanostructures.
We thank the referee for thoroughly evaluating our manuscript.

The spatial visualization of confined electric fields and dimensional crossover in carrier transport is a useful experimental contribution. However, several of the underlying physical concepts invoked, such as thickness-dependent transport in WSe_2 and carrier injection across 2D/III-V heterojunctions, are not new in themselves. The novelty primarily lies in nanoscale EBIC mapping of engineered hybrid-dimensional junctions, rather than in a fundamentally new heterostructure concept or in the direct demonstration of quantum optoelectronic functionality. The authors should clearly highlight the novelty of their study.
We thank the reviewer for the comment, which is highly pertinent. We partially agree. In our view, the demonstration of effective charge separation when interfacing 2D layers with monolithically integrated in-plane nanostructures is also a point of novelty. We modified the abstract and the relevant section of the introduction accordingly.

*** Changes in the manuscript, abstract**
"We showcase here an innovative approach to accurately ~~engineer~~ characterize electrically active nanoscale regions in multidimensional heterostructures made of WSe_2 layers and GaAs, thin films and in-plane one-dimensional nanostructures."

*** Changes in the manuscript, page 2**
"In this work, we tackle two major limitations to large-scale production of TMD-based devices: localized electrical injection and nanoscale investigation of layered semiconductors. We propose to use III-V nanostructures to engineer carrier injection with nanometric accuracy in pre-defined positions. We ~~engineer vertical heterostructures composed of~~ focus on the built-in field at the heterointerface between 2D WSe_2 layer on epitaxial GaAs, which form a vertical heterostructures. Selective area epitaxy tunes the geometry of nanostructures through the design of the mask opening[17–19], thus enabling 0D, 1D and 2D heterointerfaces."

My other concerns are given below, which the authors should address before the manuscript can be considered further.

1) It will be good to through light on the nature of the heterojunction. Is it a van der Waals-type junction, or is it more like a conventional p-n junction type? Does it depend strongly on thickness?

We think the system is best described as a conventional p–n junction. AFM measurements in fact reveal a gap on the order of a few nm between the two materials, which prevents the formation of interlayer excitons. We thus expect excited e^-/h^+ pairs to require a built-in field to cross the heterointerface and be collected in the external circuit. As AFM did not reveal any significant adhesion difference as a function of thickness, we expect this mechanism to be valid for all the terraces. Another element pointing in this direction is the observation of EBIC collection at the outer edge of the heterostructure, consistent with the drift/diffusion model of a conventional p–n junction. For example, the SEM/EBIC (overlaid) image below highlights the presence of electrical signal on in-plane nanostructures not only in the regions where the two materials overlap but also next to the edge of the 2D material.

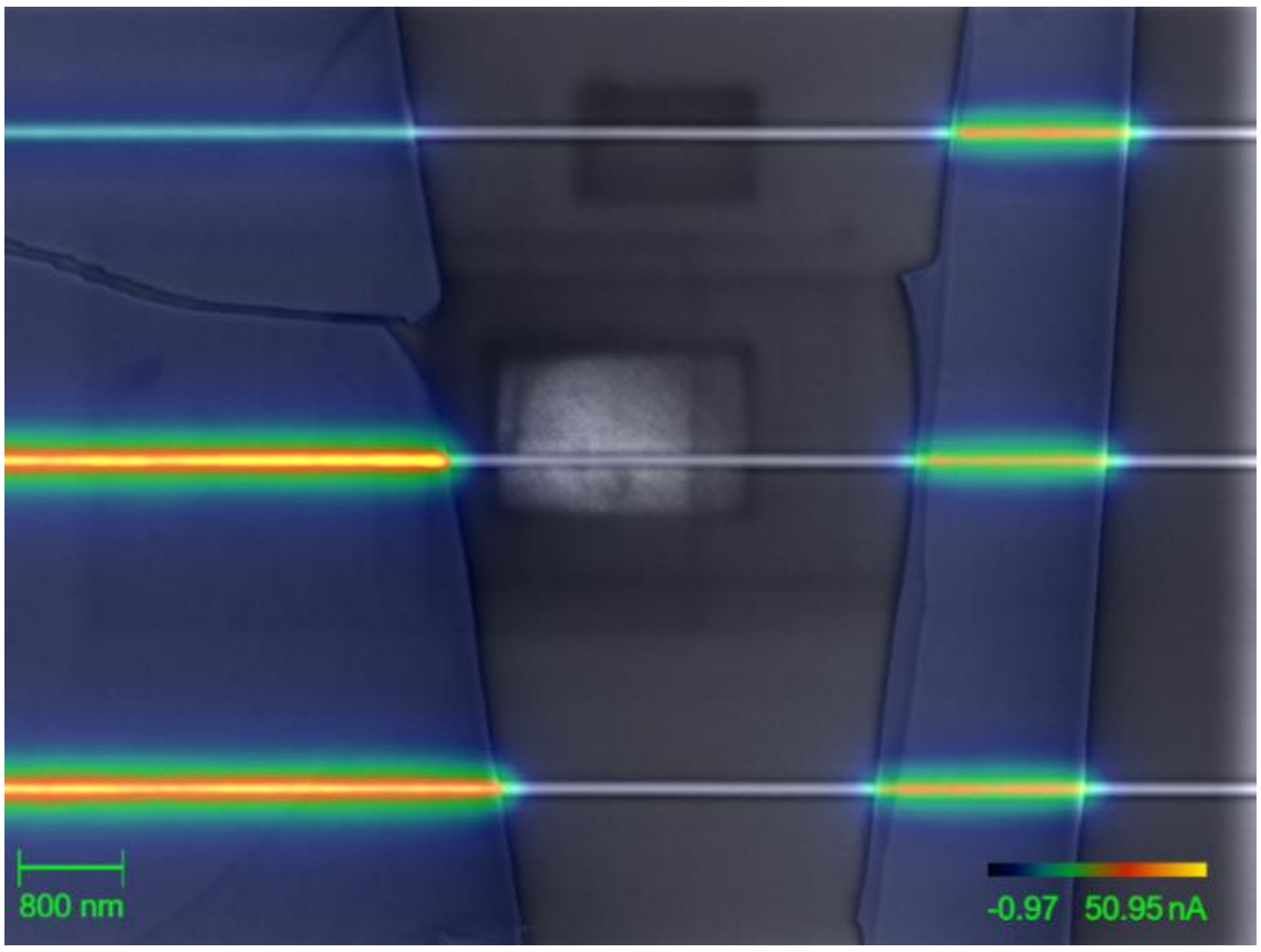

2) Is there any formation of depletion layer at the junction? If that is the case, then what is the extent of this layer?

Using the model for conventional p–n junctions, we expect the formation of a depletion layer across the interface between GaAs and WSe_2. Due to the vertical configuration of the junction, it extends parallel to the primary beam; thus, its extension cannot in this configuration[1]. The electric field direction here is out of plane, while the EBIC maps resolve in-plane electric features. Tuning the acceleration voltage can provide information on the depletion region extension in the WSe_2, being the top layer. The close agreement between the flake thickness at which the EBIC signal disappears, and the simulation of the interaction depth suggests an ultra-thin depletion layer (below 10 nm).

Unfortunately, we cannot provide a quantitative estimation of the depletion region. This would require scanning the electron beam on the diode cross section. Such a measurement—conventionally performed on bulk devices such as solar cells—cannot be applied in this case without major technical developments to avoid damage the 2D layer and artifacts associated to its limited thickness.

3) What is the mechanism behind the crossover of transport behaviour exactly at 15 nm thickness?

The crossover at 15 nm thickness corresponds to an optimal trade-off between electric field strength and extension of the interaction volume. The interaction volume is shown to penetrate roughly 40 nm into the top WSe_2 layer under these measurement conditions. This implies reduced overlap of the penetration depth with the heterojunction when increasing thickness of the 2D flake. At the same time, the strength of the electric field decreases with flake thinning. These two factors contribute oppositely to the generated current, which reaches its maximum at a flake thickness of roughly 15 nm in this experimental configuration.

For WSe_2 thicknesses below 15 nm, the built-in field intensity decreases to a point where charges diffuse laterally to the closest active junction. At 15 nm and above this threshold, charges flow towards the heterointerface, thus in the out-of-plane direction.

4) The layered nature of WSe2 will cause highly anisotropic transport when considered in the in-plane and out-of-plane directions. Can the author discuss what the effect of such confinement on in-plane transport is? Also, do the authors observe any crossover

Indeed, we expect in-plane and out-of-plane transport to have highly different characteristics.
It is widely accepted that 2D materials have out-of-plane resistivity much larger than their in-plane resistivity. Explanations ranging from electron density in vdW gaps, flat energy dispersion, and tunneling and hopping transport mechanisms are used to describe the unfavorable conduction through layers[2,3]. In turn, one would expect a larger out-of-plane series resistance. Although most studies focus on majority carriers, it is reasonable to apply the same arguments to minority carriers. This would translate into a larger in-plane minority carrier diffusion length.

Unfortunately, our experimental setup does not allow us to draw conclusions regarding either the series resistance or the variability of the minority carrier diffusion length along the two perpendicular directions.

Series resistance is commonly considered to decrease the carrier collection efficiency, i.e., the signal-to-noise ratio, in EBIC measurements. This arises from the total series resistance, which is commonly limited by the contact resistance when using direct contact with nanometric tips. For the same reason, current–voltage characteristics are ineffective in describing the in-plane transport in this type of experiment.
Minority carrier diffusion length measurement by EBIC, instead, has been extensively used to investigate factors affecting carrier mobility, even in nanomaterials. A remarkable example is the work of Gutsche et al., who used EBIC and quantitative measurements of minority carrier diffusion length to quantify surface recombination rates[4]. However, the planar symmetry of the vertical heterostructure investigated in this work does not enable visualization of out-of-plane diffusion, thus hindering any comparison. For the same reasons no crossover can be observed.

We thank the referee for the comment which encourages us to enrich the discussion in the main manuscript as follows:

*** Changes in the manuscript, page 5**
"Diffusion tails in EBIC micrographs can be observed when the primary beam is perpendicular to the built-in field vector[27,36], which is not the case for vertical diodes. The planar symmetry of the stack prevents visualization of the out-of-plane diffusion transport and should hinder lateral diffusion (as excited charges are expected to flow towards the heterointerface). The identification of in-plane diffusive transport in this system therefore implies a significant weakening of the vertical built-in field in thin WSe_2 terraces."

5) The effect of strain is an important point which needs to be discussed in greater detail. The author can fabricate samples of the same width but different heights to determine how strain affects the observed behaviour.

Strain in TMDs on nanomembranes has been the object of a previous study. Raman and photoluminescence spectroscopy measurements showed a maximum deformation below 0.3% for a MoS_2 monolayer on similar geometry nanostructures which have a limited aspect ratio which is around 0.25 (50 nm tall and 230 nm wide)[5]. We could not observe any significant intensity variation associated with funneling and thus strain. Deformation is expected to be even less prominent with increasing thickness. We therefore do not expect any relevant strain variation in the present study.

Reviewer #2 (Remarks to the Author):

We thank the referee for thoroughly evaluating our manuscript.

General Comments/Question:

- Has author also tried MoS2 which is well known as a n-doped TMD?

No, we entirely focused on WSe_2. Indeed, the findings of our work open the path to the investigation of several material stacking including MoS_2. In this work we decided to limit ourselves to WSe_2/GaAs heterostructures.

- Has author try to encapsulate the WSe2 with hBN on top and leave the small corner exposed so the tip can touch the WSe2 ?

We would like to thank the reviewer for this comment. We completely agree with the reviewer that this experiment would reinforce the description of the system. We therefore performed the experiment as suggested. The results are reported in SI (Supplementary Figure 11). Here below we briefly summarize the findings.

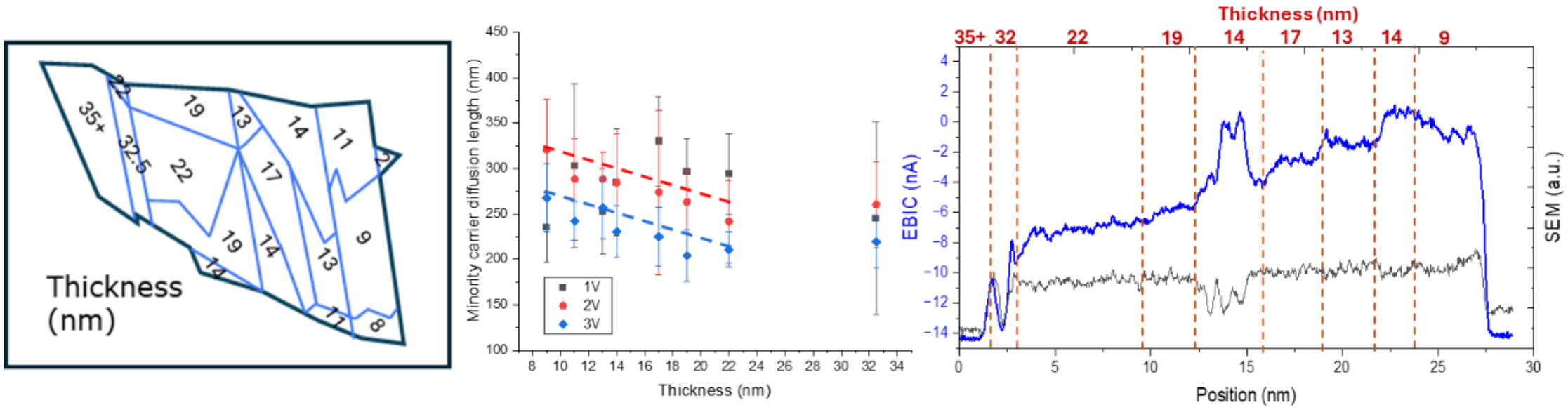

We stacked a WSe_2 flake containing several terraces (left panel) with 13nm thick hBN and transfer the ensemble on GaAs nanomembranes. We left the thick edge uncovered to get electrical access and we were able to acquire EBIC maps (reported in Supplementary Figure 11). The analysis of transversal current profiles enabled to extract hole diffusion length values (central panel) comparable to the case of planar heterostructures (300 nm). Diffusion length is found to decrease with increasing thickness although the relative variation is lower than the case of bare WSe_2. Longitudinal profiles (right panel) show absence of diffusion regimes and remarkable collection of current from thin terraces, indicating efficient carrier transport by drift even on thin areas. This represents the most relevant difference with respect to uncovered WSe_2. Overall, the measurements indicate a significant impact of hBN encapsulation which appears to strongly mitigate – but not preventing completely – the thickness dependent effects discussed in the main manuscript.

For this reason, we reinforced our conclusion by adding a section with these findings into the Supplementary Information and referring to it in the main manuscript as follows.

*** Changes in SI: added Supplementary Fig.11**
*** Changes in the manuscript, page 5**
"However airborne contaminants and molecules have been reported to have a thickness-dependent impact on carrier concentration33 consistent with our work. Measurements performed on hBN-encapsulated samples strongly support the link between the observed vanishing electric field and contaminants on the top surface (Supplementary Fig.11)."

Due to her contribution to this key experiment, Matilde Cantele has been added as a co-author to the manuscript.

- Does it matter where to place the tip for the MW devices? I would suggest author label the location where the tip is touching

The tip position can have severe impact on the measurement only under improper contact conditions. First, the contact should not pierce through the 2D flake, which would cause short-circuiting of the junction. In this case, the measurement would not provide any current extraction at the junction. For this reason, thick regions (t > 50 nm) are chosen to land the tip. Second, the touchpoint needs to be far enough from the investigated region, as the mechanical contact may cause local damage that must not propagate toward the region of interest. Thus, the position of the tip generally lies several microns away from the fields of view presented in the SEM/EBIC images of the manuscript.

Provided that these events do not occur, the in-plane tip position does not affect the current extraction, since the in-plane resistance of the 2D material is expected to be orders of magnitude smaller than the contact resistance. This allows us to consider the series resistance from WSe_2 as negligible with respect to the other contributions. This description applies to planar and nanostructured heterostructures likewise.

Electrical mapping of 2D/3D heterostructures:

"The electrical response through in-situ tungsten tips mounted on nanomanipulators"

- How do authors make sure the tip is just touching the surface? and not poking into the layers? If it did indent into the layer will this change the result? I would assume the better way might be transfer a piece of graphite to touch the WSe2 and the tip can just touch the Graphite ? have author try this?

Achieving electrical access on 2D semiconductor via direct contact with a nanoprobe is a key technical aspect that we developed for this work. We found that acquiring absorbed current maps (from primary beam) is the most reliable strategy to assess whether electrical contact is established. An example is given in the figure below.

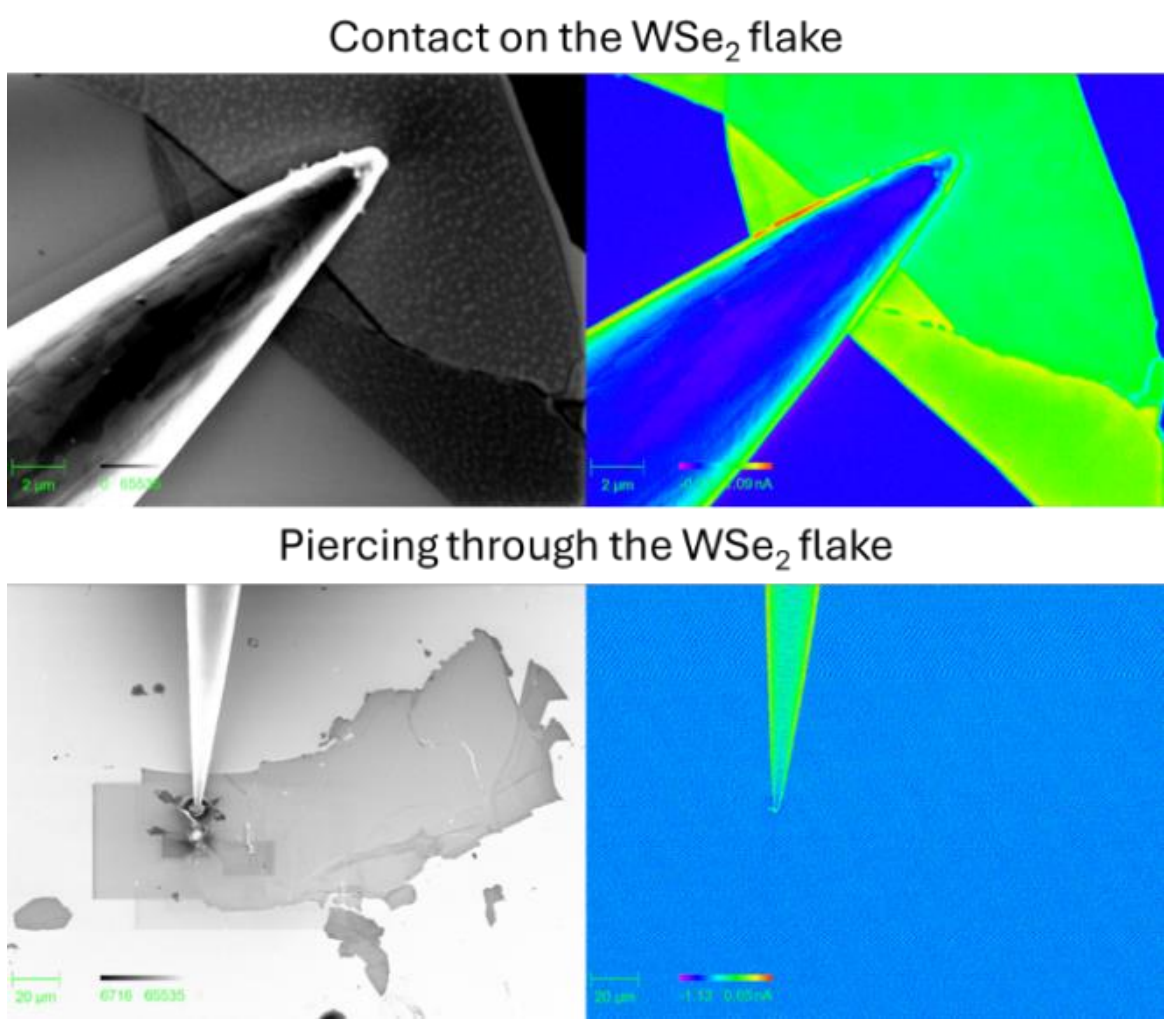

The mechanical contact with the 2D material can result in no or partial damage of the flake (with formation of indents) depending on the pressure exerted during the tip landing. Provided that the tip does not pierce through the flake, local damages at the tip location has little impact on the measurements as it just affects the contact resistance (thus the signal-to-noise ratio). We demonstrated this also in a recent

publication where the same contact strategy has been used to visualize and study lateral pn junctions in 2D materials[6]. Moreover, mechanical contact has been reported to clean locally 2D interface with benefit in terms of contact resistance[7]. In case of piercing, instead, one can observe the complete signal loss (see figure). No Schottky is observed at the p-GaAs/W interface as expected from literature[8].

While the idea of using a graphite contact would ensure homogenous access and damage-free electrical contacts, graphite would also alter the equilibrium concentration in WSe_2 adding a degree of complexity to the data interpretation.

> *"The presence of a gap at III-V/TMDs heterointerface due to hydrophilic adsorbates[5], hinders the assessment of the real WSe_2 thickness" Photoluminescence (PL) measurements excluded the presence of monolayers (Supplementary Fig. 5).*

- Has author do any post cleaning process after exfoliation to ensure the interface between WSe2 and epi-GaAs are clean? Such as annealing? and what does the PL of WSe2 looks like? I would assume the intensity should be really low ? Also , has author try to encapsulate the WSe2 with hBN on top and leave the small corner exposed so the tip can touch the WSe2?

We thank the reviewer for addressing this critical point. Our fabrication process includes steps to mitigate contamination of the III-V surface. The epi-GaAs substrates are stored in vacuum after growth. They are kept under vacuum until the moment of the transfer, thus preventing from major sources of long-time contaminations. Moisture and airborne contaminants could deposit on the surface while moving the sample to the transfer station (maximum 30-60 minutes). Substrate is heated at 40°C during our 2D transfer thus acting as a mild surface dehydration step. We intentionally excluded surface passivation steps and annealing steps from the fabrication flow after thorough investigation.

We performed passivation tests with an ammonium sulphide solution, known post-processing surface passivation treatment for III-V[9]. XPS analysis of the surface did not reveal any relevant modification of the surface composition after the dehydration step. Annealing instead has been proven effective to reduce the the gap between the flake and the substrate – as measured with AFM - but does not remove it completely. Neither of these steps was effective in removing contaminants at the III-V/TMD interfaces thus not justifying to increase the complexity of the fabrication flow.

Concerning PL, the reviewer is correct and the signal from WSe_2 was too weak to be observed with the acquisition times used for the measurements. This is expected as the PL intensity of WSe_2 decreases very rapidly for thicknesses superior to a monolayer. As this was beyond the scope of this study, the PL signal was not explored any further.

Regarding the comment on hBN top encapsulation, we refer the reviewer to our previous answer on a similar comment.

> *" Monte Carlo simulations indicate an electron beam penetration of ~40 nm (Supplementary Fig. 6), elegantly matching these experimental observations"*

- I assume this is under Vacc~2V correct ? author might need to mention it in the main text, Then I would suggest adding the Vacc= ?? in all the figures.

We thank the reviewer for the remark. We added the information about acceleration voltage (2-3 keV) and probe current (~0.4 nA) throughout the manuscript and the SI.

- Based on the SI-6, does that means using the higher Vacc would give you different result in figure 3(b)

Yes, this is indeed correct. Different acceleration voltages change the penetration depth, thus affecting the overlap with the heterointerface and the current generation. This is consistent with the Casino

simulations for the interaction volume (Supplementary Figure S6) and the EBIC maps acquired at different V_{acc} (Supplementary Figure S7) where current collection is extended towards thicker regions of the 2D flake.

Electric field confinement in 2D/1D heterostructures

- Looks like the NWs are ~50nm tall, what is the WSe2 thickness used in the figure 4. I think author has them in the SI, but it is better to add a label in the figure 4(a), again easier to get the info quickly for the readers.

Thanks, we addressed this comment with the following change:

*** Changes in the manuscript, page 7**
"Transfer of WSe_2 flakes on GaAs NMs leads to 2D/1D heterojunctions. We investigated multiple samples containing terraces with thickness ranging between 10 nm and 30 nm. Figure 4a shows the EBIC map of one sample containing three WSe_2 terraces (11 nm, 17 nm and 26 nm as reported in Supplementary Figure 13) where localized 1D heterojunctions can be clearly visualized."

- Does it matter where to place the tip for the MW devices? I would suggest author label the location where the tip is touching

The reviewer accidentally added twice the same question in the review file. This question has been addressed earlier in this document.

Bare GaAs NMs (red star) display narrow SE and AFM profiles, consistent with the <101> facets, and exhibit negligible electrical signal. WSe_2/GaAs NMs (yellow and orange stars) instead can be identified by a weak SE signal due to the screening by the top flake and a wider AFM profile due to the imperfect 2D-on-1D conformation[34]

- Does author mean SE signal as SEM signal? In the text compared to figure 4b?

Yes, we corrected the typo. Thanks for the noticing it.

"EBIC signal peaks at these locations and sharply goes to noise level in between structures, demonstrating effective in-plane confinement of the space charge region. Photoluminescence maps show consistent emission quenching at these locations (Supplementary Fig. 11)"

- Figure S11 need to show what is the color bar and maybe add dashed lines to ID where the NM located? I assume the color bar is for the PL intensity, it is quite confusing without those info. For example: I would assume the dark lines are the NW location ? then why does it has lower intensity ? is it because of the NW width ? Has author considering varying the width and height of the NW and see the PL quenching effect changes ?

We modify Supplementary Figure 11 (now Supplementary Figure 13) as reported below. We highlight the NM locations with lateral arrows instead of dashed lines which would decrease the readability of the image.

The lower intensity at the NM locations below the WSe_2, flake, is a well-known consequence of the built-in-field created between the WSe_2 and the NMs in the areas where these are interfaced. Charge separation of the photogenerated carriers decreases recombination rate thus leading to a quenching of the PL emission. Therefore, varying the width of the NMs would increase the contact area between WSe_2 and GaAs and the darker areas would appear wider. Meanwhile, increasing the height of the NMs

may reduce the contact area between the materials, due to a loss of conformation during the transfer because of the high aspect ratio, and thus the dark areas may appear thinner.

*** Changes in the Supplementary Materials, Supplementary Figure 13**

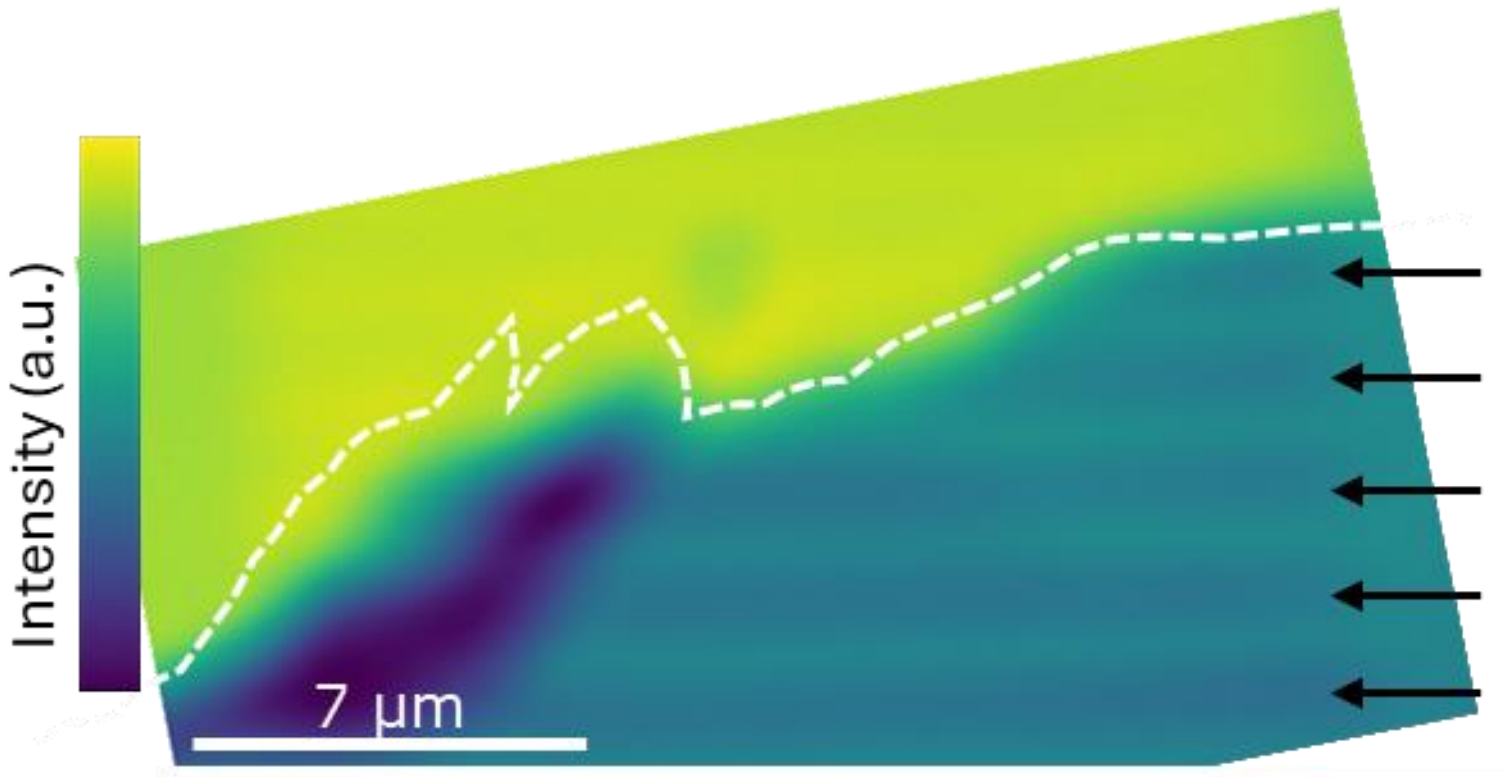

"**Error! Reference source not found.** shows ~~map of the GaAs PL intensity of a sample consisting of a WSe_2 flake on GaAs NMs. The flake is outlined in a dashed white line.~~ intensity map, indicated by the colorbar of the integrated PL emission of GaAs over the range 850 nm – 900 nm. The sample is a WSe_2 flake, outlined in a dashed white line, on GaAs NMs whose position is indicated by the lateral arrows. The NMs appear as bright horizontal lines outside of the flake boundary and as dark horizontal lines within the flake boundary. It can be seen that the GaAs emission intensity is reduced by the presence of the flake (area under the white line), with thicker regions of the flake appearing darker."

- Author mentioned *"These findings remarkably demonstrate a tight correlation between the adhesion of WSe_2 on GaAs and the carrier transport."* Can you explain further ? do author means this quenching only happens if the WSe2 has good contact with GaAs-NM?

Yes, the reviewer is correct. The EBIC and PL measurements show that the observed effects (EBIC signal peak and PL quenching) only occur in the areas where WSe_2 is in good contact with the GaAs nanostructures. This is expected as both radiative recombination quenching and carrier flows across the heterostructures originates from the built-in field established when the two materials are placed in contact. Our measurements – in particular the ones under primary beam exposure – allows to visualize with outstanding resolution the correlation between the functional behaviour (charge flow/luminescence quenching), position of the nanomembranes and WSe_2 coverage.

- I would also suggest to crop the figure 4a and rotate to match the same with figure S11 or the other way around. So reader can follow it more efficiently.

We decided to rotate both images to force the elongation direction to be parallel to the manuscript lines. As a result, figure 4a is zoomed as requested in a following comment.

The main manuscript and the SI have been updated with the new Figures, reported here below

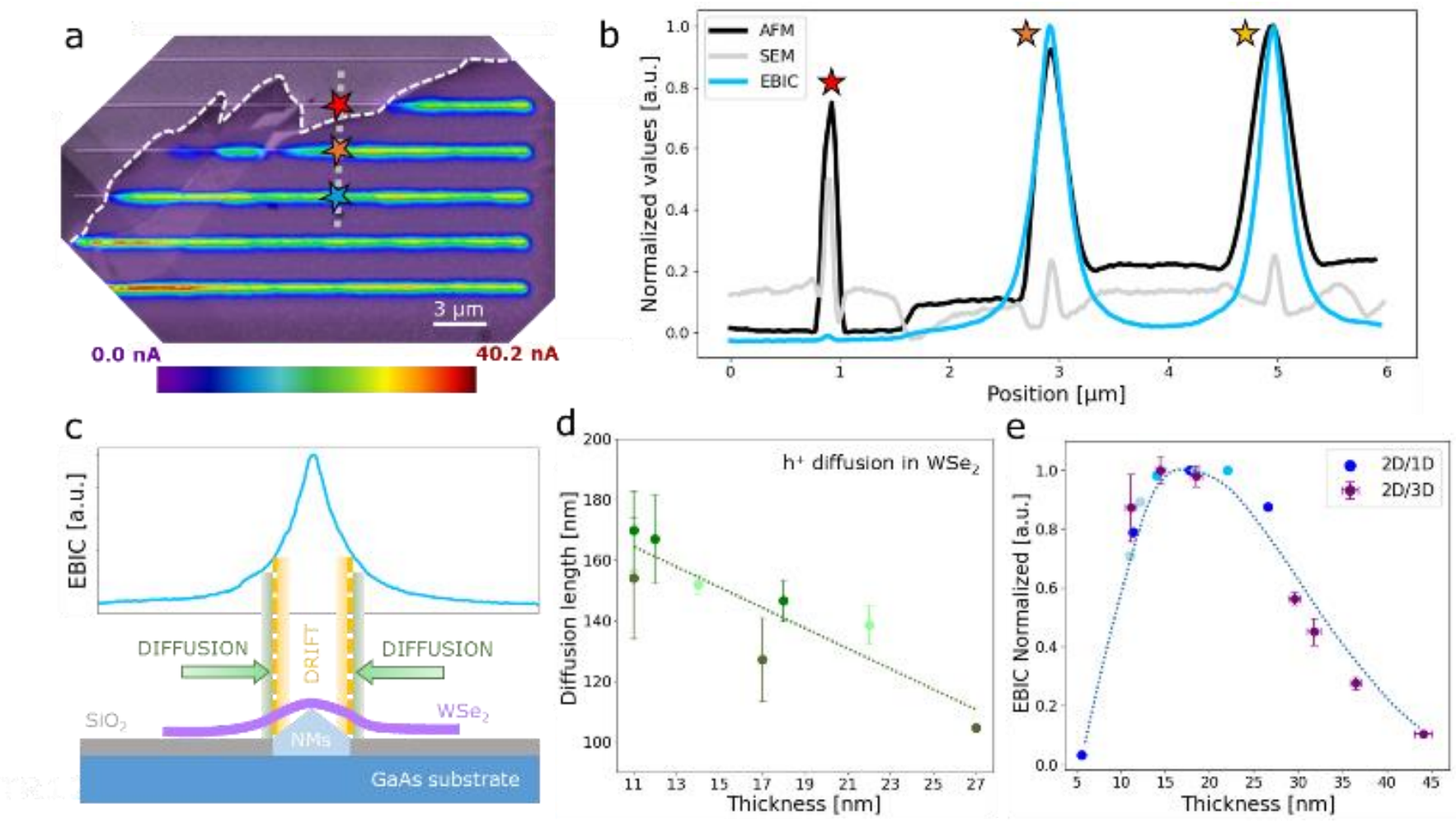

.

- I am not sure if it is a good plotting style for figure 4b as the y axis represent different unit? Maybe waterfall plot is better?

We tried to plot the different profiles in a waterfall fashion. However, it is unclear whether this visualization adds value or clarity. The plot in its current form allows to appreciate the spatial correlation between AFM and EBIC signals when relevant. Even if these profiles do have different units, the overlap of renormalized curves allows to naturally show the strict relationship between morphology and charge transport.

We thank the reviewer for the suggestion, but we decide to maintain the current style.

- I would also suggest to have a zoomed in figure 4a so reader can see it more clearly

As a result of the data rearrangement in Figure 4, panel (a) has been enlarged and zoomed in for improved clarity and to better guide the reader.

- In the over lay background SEM image of the figure 4a, I understand the bottom left corner shows the dark boxes due to the SEM scanning window(dark dashed line). But Why there is a brighter square boundary in the MW area(white dashed line). ? see below

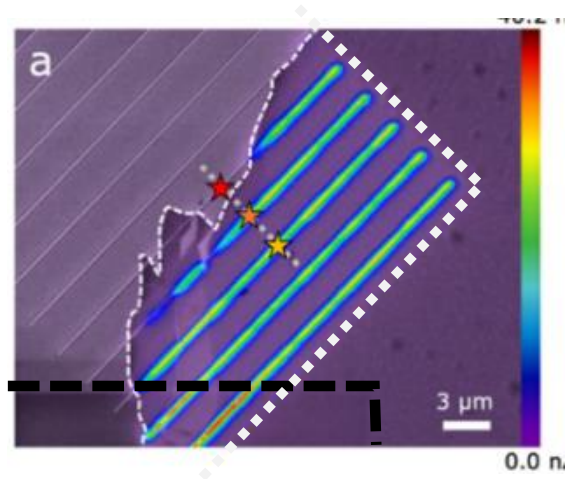

The white contrast is associated with the fabrication of the nanostructures. We believe it may originate from the HF etching step, which can laterally reach the oxide/substrate interface from within the openings. This creates a minor difference in the SiO_2/GaAs interface near the nanostructures compared to regions farther away. The difference is highlighted by secondary electron emission efficiency, appearing as a faint halo around the nanostructures and the array. Below, we show a SEM image of a sample—not used in this work—displaying the same feature, even around the top label. No impact on morphology (oxide thickness or roughness) was observed. Figure S10 reports AFM maps and profiles of bare nanostructures prior to transfer, where no features are detected around the nanostructures. We therefore expect no effect on WSe_2.

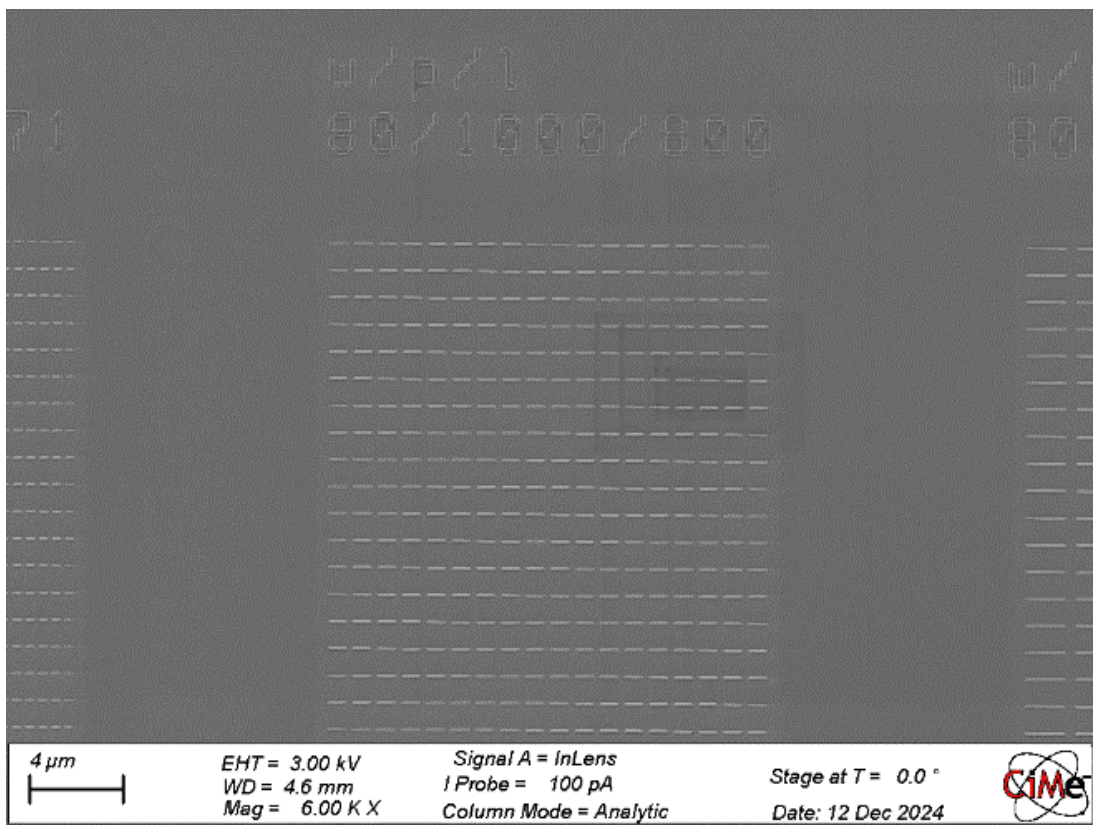

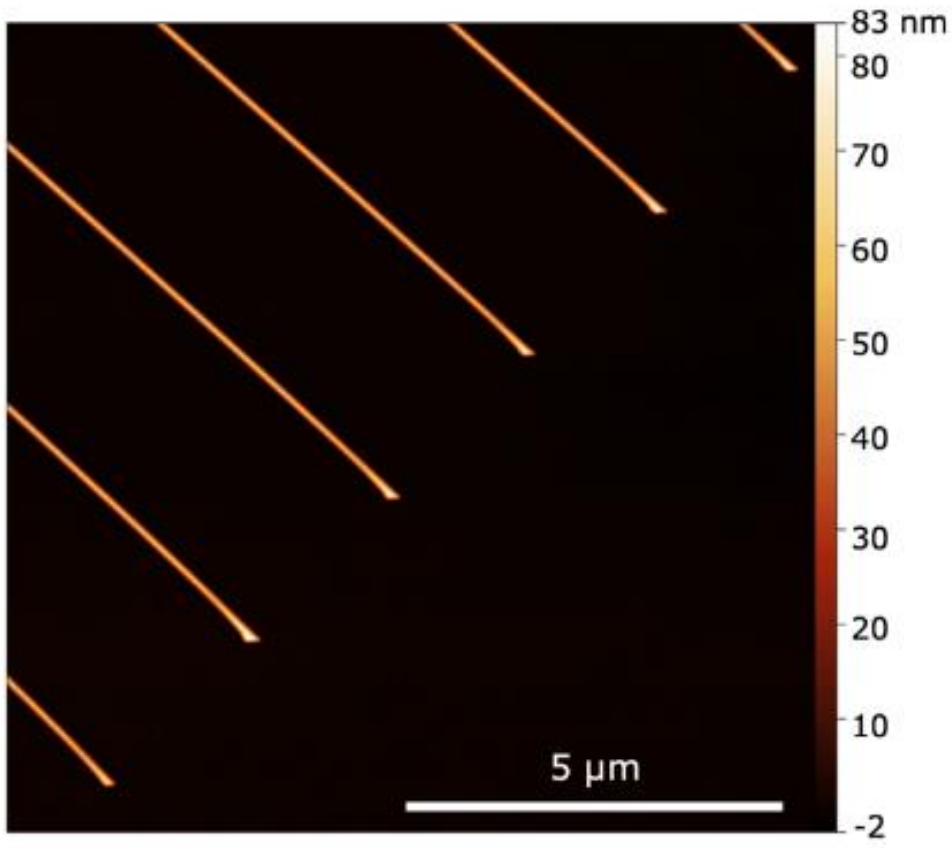

Thicker regions instead appear to result in stronger confinement (blue curve of Figure 5d), most likely due to the reduced conformity of more rigid flakes (Supplementary Fig. 19). Thickness therefore appears to play a significant role also to provide mechanical stability at the nanoscale

- Looks like fig5d and figure S19 is not consistent. Please check

We thank the reviewer for noticing the inconsistency. Figure 5d is indeed correct while Figure S19 presented inverted labels. The figure in the Supplementary Information has been corrected.

Conclusions : NA

Methods

WSe_2 exfoliation and heterostructures fabrication

- *Once contact is obtained between flake and substrate, the substrate stage is heated up to 90°C for 5 minutes to increase adhesion*

I am not sure if the heating the really necessary or beneficial for such a transfer, usually heating is required for the polymer dry transfer such as using PC, PPC, etc…. For directly transferring the TMD from PDMS to the target substrate , you should not need to do any heating. I would suggest Author to try it. It can also reduce any Si contamination from PDMS residue.

We thank the reviewer for this suggestion. Transfer on nanostructures has been found to be more challenging than on flat substrates. We think that this may be related to the limited contact area between the flake and the substrate. We have found empirically that heating improves the sticking of the flake to the substrate in these circumstances. The methodology here presented has been optimized over the years to be effective over a variety of substrates including SiO_2 and III-V. This allows to maintain transfer parameters identical for all samples presented in this work. Although the reviewer is correct and temperature can increase PDMS residues, this will not affect the interface between the flake and the substrate as it is never in contact with PDMS.

Comments To The Figures:

Figure1.

- What is SCR in the (i) label?

SCR refers to "space charge region". We now introduce the acronym in the caption. The changes in caption are reported as an answer to the comment below.

- Authors need to explain what those color coded block means are. For me it is a quite confusing and I am also assuming readers won't catch the idea by simply looking at those cartoon images.

We thank the reviewer for the comment. We modified the introduction to clarify our message.

*** Changes in the manuscript, page 2**

"EBIC exploits a primary electron beam to locally excite excess charges, which are then collected as induced current under the effect of the built-in field ~~(Figure 1)~~ [21,22]. Figure 1 presents the working principle of EBIC highlighting key relevant parameters ~~and functional aspects~~ such as depletion region extension[24], charge concentration[25,26] and minority carrier diffusion length[26,27](panels i and ii schematically represent planar junctions with built-in field and space charge region localized near the heterointerface). EBIC is also extremely effective to localize rectifying interfaces and associated charge transfer in-plane (panel iii) and in depth (panel iv) thanks to the ~~limited~~ reduced size of the interaction volume where the charge generation occurs[23] granting a spatial resolution down to few tens of nanometers."

We also modified the caption to clarify further the visual elements in the Figure.

*** Changes in the manuscript, Figure 1 caption**

"Figure 1. Working principle of EBIC mapping. The central schematic displays a primary electron beam exciting e-/h+ pairs due to inelastic scattering within a nanometric interaction volume. Excess charges in the space charge region drift apart while excited pairs in the vicinity of the junction diffuse. Both flows contribute to electron beam induced current. Panels i and ii depict schematically planar junctions with varying carrier concentrations across the junction. ~~This technique is~~Being sensitive to the strength of the electric field ~~and thus to the carrier concentration across the junction~~, EBIC can define the extension of the space charge region (SCR) (panel i). ~~EBIC~~ Line profiles orthogonal to the rectifying interface also offer a way to measure ~~of~~ the minority carrier diffusion length (panel ii). Panels iii and iv highlight the functional advantages of EBIC when investigating localized rectifying elements. In case of ~~localized~~ junctions localized in the plane, maps display active and inactive regions with lateral resolution defined by the interaction volume (panel iii). Instead, by varying the energy of the primary beam, the penetration depth can be tuned thus accessing buried electric fields (panel iv)."

Figure3.

- I would suggest try to combine fig3 c and d, or maybe just adding thickness range in (d) for each region? So the reader can easily get the main info.

We thank the reviewer for the suggestion. We improved Figure 3c by adding the thickness values for each terrasse and the boxes indicating the type of charge transport in each thickness range consistently with Figure 3d. We modified associated caption as indicated below.

*** Changes in the manuscript, Figure 3 caption**

"(c) EBIC and AFM line profiles along the grey dashed arrows in panels (a) and (b). Each terrasse is delimited by dashed lines. The thickness of the terraces is indicated by the embedded values (in nm). The correlated plot highlights the current variation within terraces with thickness below 15 nm, signature of a diffusive transport. In contrast, current is constant on terraces thicker than the threshold thickness. Four regions can be defined where charge transport is dominated by pure diffusion (green), mixed diffusive/drift transport (violet box), pure drift (yellow and purple boxes, differing from optimal and suboptimal excitation). The color of dashed lines and thickness value reflect the color code used for the transport regimes. Points represent the current values averaged over the area of each terrace. Error bars represent the current variability on the terraces. (d) Comprehensive 3D schematic of carrier generation and collection mechanisms on terraces with varying thickness. The orange layer overlapping

with the epi-GaAs and partially overlapping with WSe_2 and with the GaAs substrate represents the space charge region. The arrows indicate the built-in field becoming less intense (thinner arrows) with decreasing thickness. Consistently with panel c, the panel describes (left to right) the diffusive transport in thin layers where the electron beam penetrates deep enough but the electric field vanishes (pure diffusion regime), intermediate regimes where current variation is sub-exponential (diffusion + weak drift), out-of-plane drift transport where current is constant on each terrace. In case of drift, we collect the highest signal (drift), a reduced signal (sub-optimal drift) or negligible signal (no current) depending on how much the interaction volume overlaps with the space charge region..”

Figure5.

- Missing figure labels for the figures above (d)?

We added a box around the sub-panels, as they are all part of panel (b) of Figure 5, as shown below.

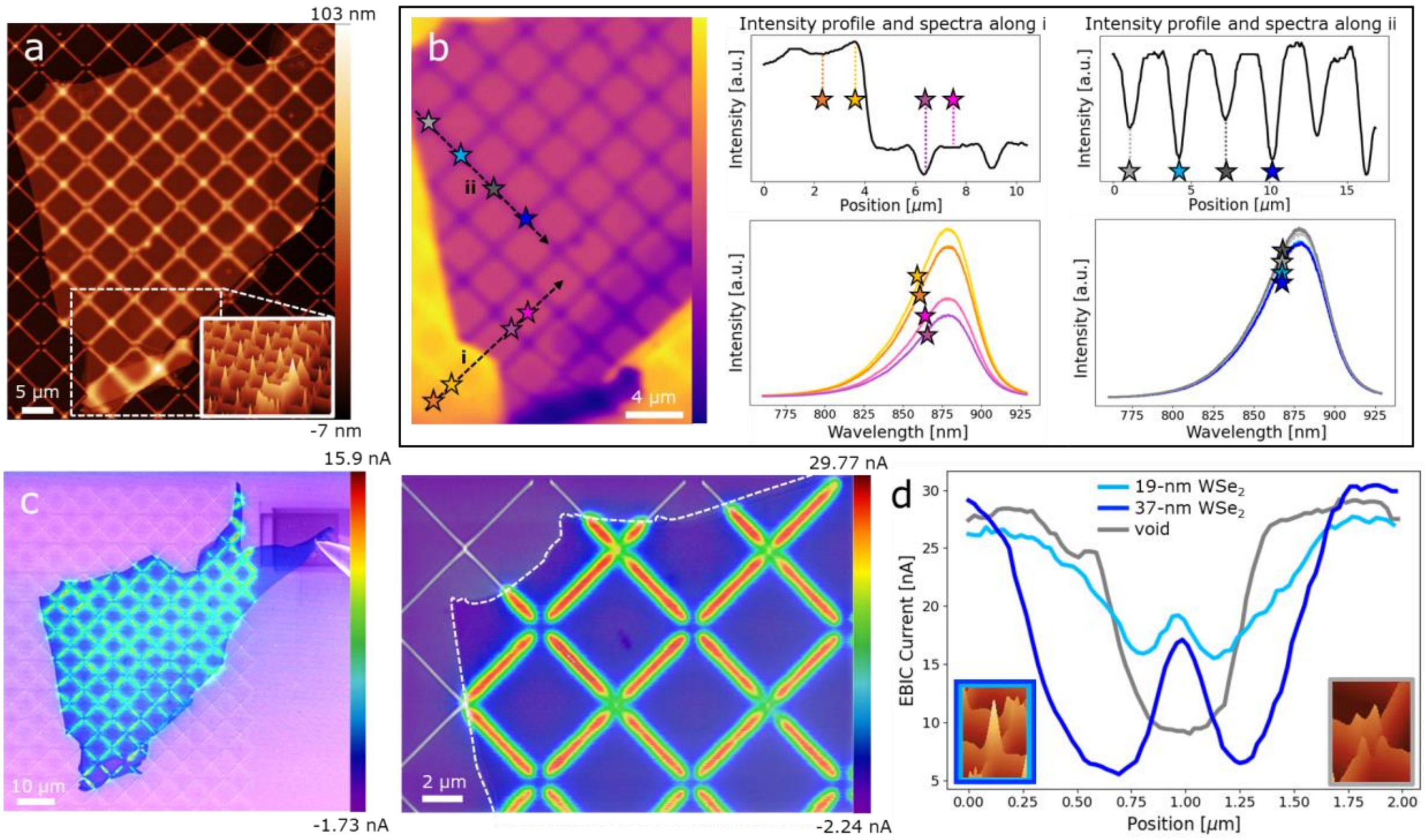

- In (c) why the current seems show different at the same area when zoomed in ?

The absolute value of the extracted current is highly sensitive to the contact resistance, which can vary slightly over time due to probe drift. The tip/WSe_2 contact is frequently adjusted during measurements to maximize the signal-to-noise ratio. Consequently, two nearly identical maps acquired under the same excitation parameters can yield significantly different current values. As an example, the EBIC maps below (in greyscale, corresponding to panel c) were extracted during the same measurement session under identical experimental conditions. Although the maps appear extremely similar, the maximum current differs substantially (30 nA vs. 52 nA). The nanoprobe position was adjusted between these acquisitions to compensate for mechanical drift, as reflected by the current instability visible toward the end of the left map.

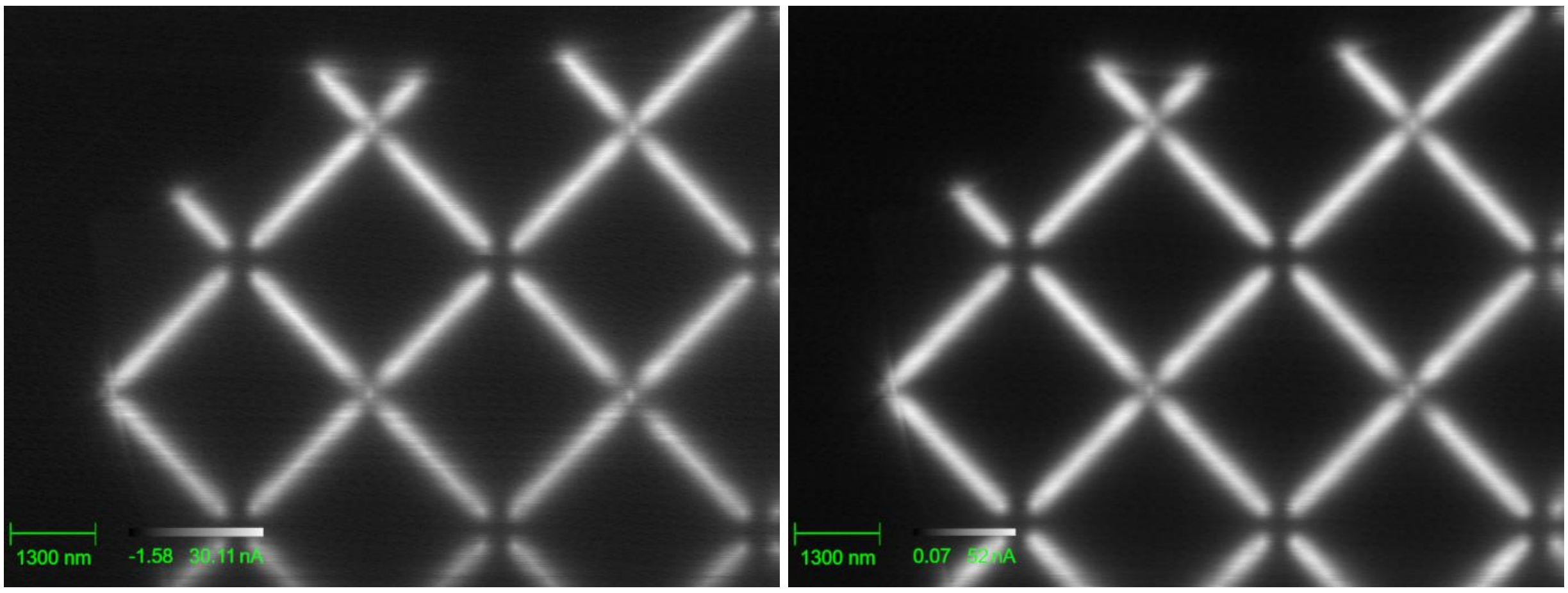

Reviewer #3 (Remarks to the Author):

This study introduces a high-resolution EBIC characterization approach for investigating multi-dimensional WSe_2/GaAs heterostructures, with significant potential to elucidate nanoscale charge transport mechanisms in semiconductor devices. While the methodology and key findings are innovative, several substantial issues require clarification and enhancement before the manuscript is suitable for publication.

We thank the referee for the positive comments and the careful evaluation of our work.

Below are my specific recommendations for revision:

1. Deepen the discussion on the physical mechanism behind thickness-dependent built-in field weakening. The explanation for the marked reduction in built-in field in WSe_2 regions thinner than ~15 nm currently remains somewhat speculative (e.g., attributed mainly to adsorbates). To strengthen this conclusion, the authors should incorporate supporting literature or provide a simplified theoretical estimate, for instance, modeling the relationship between carrier concentration and thickness. Adding a concise paragraph in the Discussion summarizing possible mechanisms would greatly improve the manuscript's rigor.

We thank the reviewer for the suggestion. Our data clearly indicate a weakening of the built-in electric field at the heterointerface. Together with the variation of minority carrier diffusion length with thickness, this evidence strongly suggests a decrease of free electron concentration with thickness. This trend matches the expected variation of intrinsic charge density with band gap but literature convincingly reports high conductivity in monolayer transition metal dichalcogenides due to surface electron accumulation[10,11]. Moreover, one can also expect the impact of external factors such as contaminants, which are to be expected since the fabrication was not performed in a glovebox and without encapsulation. The interplay of all these factors makes extremely difficult to model the trend.

We performed an additional experiment - which reinforces our conclusion - where WSe_2 on GaAs nanostructures is encapsulated with hBN. A thick portion of hBN is left uncovered to enable electrical access. The results are reported in SI (Supplementary Figure 11). Here below we briefly summarize the findings.

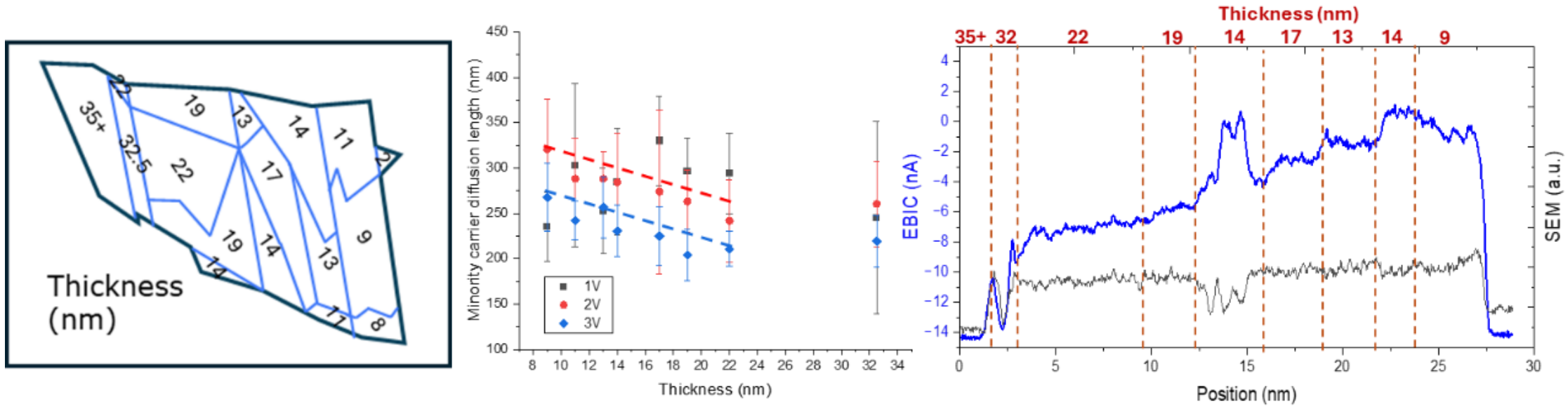

We stacked a WSe_2 flake containing several terraces (left panel) with 13nm thick hBN and transfer the ensemble on GaAs nanomembranes. We left the thick edge uncovered to get electrical access and we were able to acquire EBIC maps (reported in Supplementary Figure 11). The analysis of transversal current profiles enabled to extract hole diffusion length values (central panel) comparable to the case of planar heterostructures (300 nm). Diffusion length is found to decrease with increasing thickness although the relative percentage is lower than the case of bare WSe_2. Longitudinal profiles (right panel) show absence of diffusion regimes and remarkable collection of current from thin terraces, indicating efficient carrier transport by drift even on thin areas. This represents the most relevant difference with respect to uncovered WSe_2. Overall, the measurements indicate a significant impact of hBN encapsulation which appears to strongly mitigate – but not preventing completely – the thickness dependent effects discussed in the main manuscript.

We therefore reinforced our conclusion by adding a section with these findings into the Supplementary Information and referring to it in the main manuscript as follows.

*** Changes in SI: added Supplementary Fig.11**
*** Changes in the manuscript, page 5**
"However airborne contaminants and molecules have been reported to have a thickness-dependent impact on carrier concentration33 consistent with our work. Measurements performed on hBN-encapsulated samples strongly support the link between the observed vanishing electric field and contaminants on the top surface (Supplementary Fig.11)."

Due to her contribution to this key experiment, Matilde Cantele has been added as a co-author to the manuscript.

2. Provide more detailed descriptions of experimental methods and key parameters. To allow better assessment of the accuracy and reproducibility of the EBIC results, it is essential to include crucial measurement parameters such as primary beam current, probe contact force/resistance, signal acquisition time, and signal-to-noise ratio. These details should be added either in the Methods section or in the Supplementary Information.

We modified the Methods section as following:

*** Changes in the manuscript, page 12**
"The electrical behavior of the heterojunctions and the charge transfer across the stack is investigated with a lithographic free approach. As-fabricated heterostructures are ~~inserted in the SEM chamber (SEM Gemini™ 300).~~ investigated via scanning electron microscopy in a Zeiss Gemini SEM 300 microscope operating in high vacuum (HV) mode. Secondary electron (SE) images are acquired with a

Gemini II column in-lens SE detector at 3 keV electron beam energy with an injection current of 100 pA (as measured by a Faraday cup). ~~where the In-situ electrical circuit is created through piezoelectrically driven nanomanipulators (IMINA Technologies) and dedicated connections.~~ Electron beam induced current (EBIC) measurements were performed on an Imina NANO platform compatible with the Zeiss electron microscope. Four miBots, each equipped with a 500 nm radius tungsten tip, were controlled individually via Precisio software. Piezoelectric motion ensured nanometer control over the electrical contacts to individual flakes. Electrical access to WSe_2 is achieved by placing the nanomanipulator on thicker regions to ensure both mechanical stability and efficient in-plane charge collection. A conductive holder allows for electrical access to the substrate. This strategy aims at minimizing the ~~impact of chemicals and~~ contaminations from common cleanroom processes (chemisorption, adsorption, defect formation[44]) on the native charge population. Additionally, the in-situ contacts induce site-specific removal of contaminants[45] and enable to test multiple flakes laying on the GaAs surface under identical conditions. ~~Electrical access to WSe_2 is achieved by placing the nanomanipulator on thicker regions to ensure both mechanical stability and efficient in-plane charge collection. A conductive holder allows for electrical access to the substrate.~~ Measurements were performed at 2 and 3 keV acceleration voltage and injected current in the range of 0.4 nA (as measured by Faraday cup). Experiments were performed by applying an external bias ranging from a maximum of -3 V to +3 V through a Point Electronic amplifier controlled by a Digital Imaging scanning system. ~~The experimental setup enables to apply an external bias to the stack and to collect charges flowing through the heterostructures. The synchronization of current recording and electron beam position, together with the simultaneous detection of secondary electrons, returns spatially correlated SEM images and electrical maps. It is important to note that charge flows recorded in this configuration can have different origins such as potential difference between electrodes, excess charge generation, primary beam absorption and other possible mechanisms related to electron beam/semiconductor interactions[31-33].~~"

The last paragraph of the Methods section, together with relevant measurement parameters, has been moved to the Results and Discussion for the benefit of the readers.

*** Changes in the manuscript, page 3**

"Upon electron beam exposure (with acceleration voltage of 2 keV and probe current ~ 0.4 nA), we measure the electrical response through in-situ tungsten tips mounted on nanomanipulators. The experimental setup enables to apply an external bias to the stack and to collect charges flowing through the heterostructures. The synchronization of current recording and electron beam position, together with the simultaneous detection of secondary electrons, returns spatially correlated SEM images and electrical maps. This lithography-free approach allows testing multiple flakes with relevant morphological features. We can thus outline the physical mechanism behind charge flow via spatial correlation between secondary electron emission (SE) and electrically active features. It is important to note that charge flows recorded in this configuration can have different origins such as potential difference between electrodes, excess charge generation, primary beam absorption and other possible mechanisms related to electron beam/semiconductor interactions[28–30]."

3. Include a discussion on the limitations of the techniques employed. A short subsection in the Discussion or Conclusion should address the inherent limitations of EBIC in characterizing 2D/3D heterostructures, for example, the trade-off between penetration depth and spatial resolution, surface sensitivity, and the influence of beam-induced effects. Additionally, a brief comment on how strain or interfacial defects introduced during sample transfer could affect the results would provide a more balanced perspective.

We expanded the discussion to include limitations of EBIC as following:

*** Changes in the manuscript, page 4-5**
"The progressively increasing overlap between interaction volume and space charge region in the intrinsic epi-GaAs layer perfectly explains the electrical signal collected from 15 nm – 40 nm thick flakes. In this regime, the current collected purely depends on the thickness of the top layer. Therefore, we observe constant current values within terraces and a step-like variation with thickness. Increasing the acceleration voltage of the primary beam allows to probe the heterostructure deeper. However, the increase of beam penetration depth is associated with interaction volume broadening which, in turn, affects the in-plane spatial resolution. Increasing acceleration voltage thus prevents accurate correlation with morphological features (see Supplementary Fig.7). For this reason, low acceleration voltage appears best suited to perform high fidelity micrograph correlation. Under these conditions, charge excitation and extracted current are more sensitive to the surface. Moreover, EBIC sensitivity is suboptimal and decreases with increasing thickness. We therefore optimized the probe current. In fact, signal to noise ratio benefits from increasing the primary beam current. At the same time, aggressive exposure induces carbon deposition and beam induced damage[35]. The chosen probe current maximizes the signal-to-noise ratio while minimizing artifacts arising from electron beam exposure (Supplementary Figure 8). These measurement conditions were found to be optimal to investigate thin terraces. Thin areas (thickness < 15 nm) exhibit different characteristics."

We also added an additional section in the supplementary material (referenced as Supplementary Figure 8) reporting stability of EBIC measurements after repeated map acquisitions. The labels of Supplementary Figures in the new document has been updated to reflect to the order of appearance in the main manuscript.

*** Changes in the SI, page 9**

"Supplementary Figure 1: SEM images and EBIC maps after repeated exposure. The top and the bottom panels report two distinct WSe_2 flakes on GaAs nanostructures. Both the top and the bottom panel display an early map (left), a map under the same bias after several acquisitions (center) and the last recording of the session (right)- commonly at a different bias. All the maps are acquired at 3 keV and probe current around 0.4 nA.

Error! Reference source not found. shows the stability of EBIC maps after repeated exposures. Two examples are displayed where the map at the beginning of the measurement session (left) is compared to an equivalent map acquired towards the end of the measurement session under the same conditions (center) and under different bias (right). Maps do not display signs of electron beam induced damage thus validating the electron beam parameters used for this study. The top example shows a current increase after multiple measurements. This behavior cannot be generalized. For example, the bottom panel displays a lower induced current after repeated exposure. It is important to note that the current collected depends on the contact resistance which in turn varies with the applied pressure. Tip position is adjusted multiple times during measurements to counteract probe drift thus the absolute value of extracted current is hardly comparable amongst different scans."

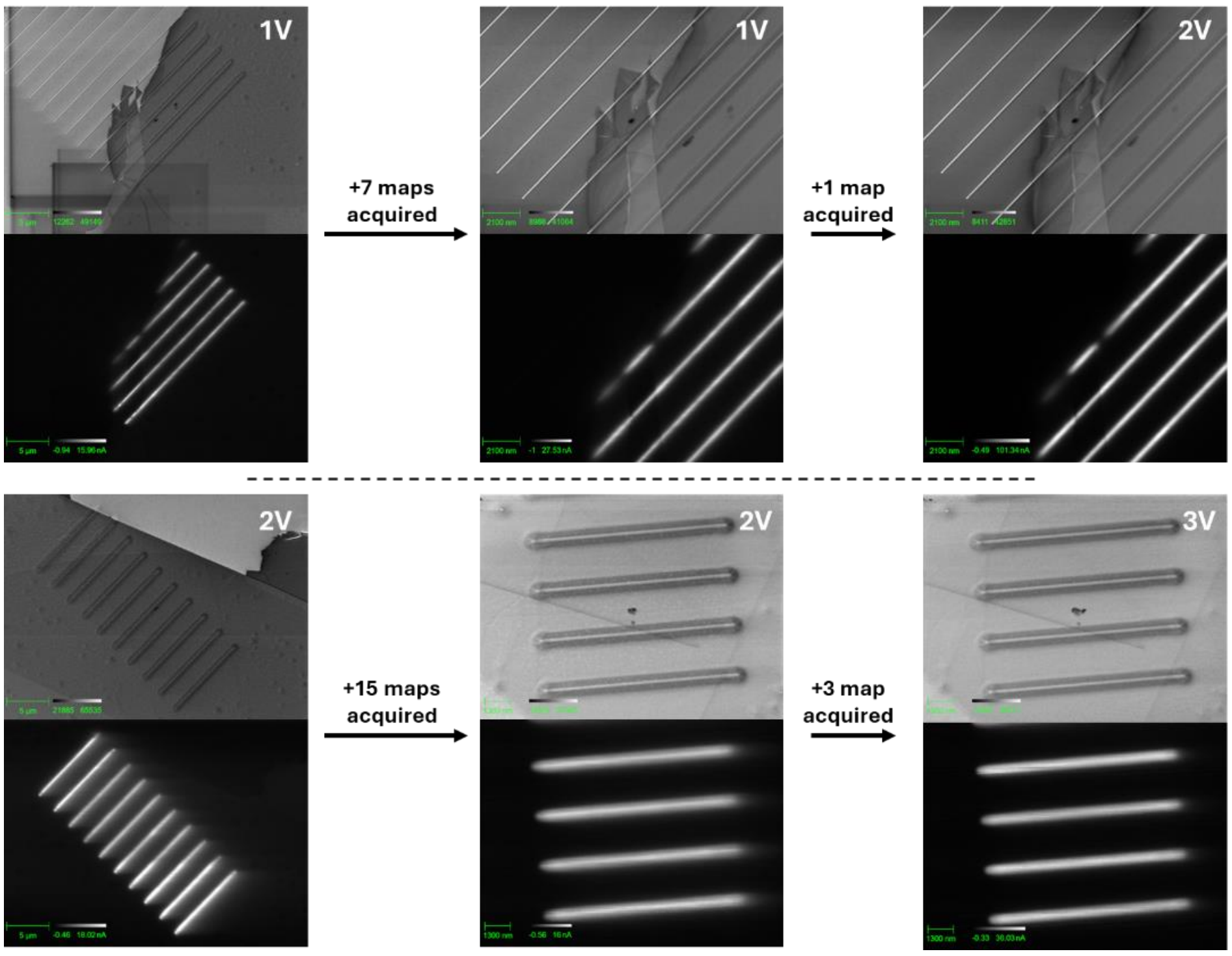

4. Improve consistency between figures, captions, and textual descriptions. Several inconsistencies were noted. For instance, the descriptions for Supplementary Figures 2 and 3 appear nearly identical and should be carefully verified and corrected. Furthermore, the explanation of Figure 3e in the main text should be clarified to better distinguish data points described as having "no physical meaning" from those with clear physical interpretations.

We improved the consistency between figures and captions throughout the manuscript and SI and adjusted the description where needed.

The descriptions of Supplementary Figures 2 and 3 which originally were left nearly identical on purpose have now been differentiated for sake of clarity.

The description of Figure 3e in the manuscript has been modified as following.

*** Changes in the manuscript, page 5**

"Electron diffusion lengths in pure GaAs as a function of the WSe_2 thickness (Figure 3e) confirms the presence of the two transport regimes. Diffusion length associated to terraces is evidently dependent on the WSe_2 thickness. This is extremely surprising as the minority carrier diffusion length in a semi-junction is not affected by the properties of the other semi-junction. This apparent WSe_2 thickness-dependent electron diffusion behavior can be explained, once again by the absence (or extreme weakening) of the built-in field for thin terraces. Charges thus diffusion is therefore not perpendicular to the edge flake and the extracted values lose their physical meaning. ~~In fact we estimate a constant L_{diff} value around 0.29±0.1 µm only for terraces thicker than 15 nm.~~ In contrast we observe a constant L_{diff} value - as expected - for terraces thicker than 15 nm, estimated to be around 0.29±0.1 µm"

References

1 V. Piazza, L. Mancini, H.-L. Chen, S. Collin and M. Tchernycheva, in *Semiconductors and Semimetals*, Elsevier, 2018, vol. 98, pp. 231–319.

2 W. Cao, M. Huang, C.-H. Yeh, K. Parto and K. Banerjee, *IEEE Trans. Electron Devices*, 2020, **67**, 1310–1316.

3 T. Kang, H. Choi, J. Li, C. Kang, E. Hwang and S. Lee, *Nano Convergence*, 2023, **10**, 13.

4 C. Gutsche, R. Niepelt, M. Gnauck, A. Lysov, W. Prost, C. Ronning and F.-J. Tegude, *Nano Letters*, 2012, **12**, 1453–1458.

5 J. Jasiński, A. Balgarkashi, V. Piazza, D. Dede, A. Surrente, M. Baranowski, D. K. Maude, M. Banerjee, R. Frisenda, A. Castellanos-Gomez, A. Fontcuberta i Morral and P. Plochocka, *2D Mater.*, 2022, **9**, 045006.

6 P. Sutter, V. Piazza, P. Ghimire, A. Fontcuberta I Morral and E. Sutter, *Adv Funct Materials*, 2025, e22666.

7 P. Schweizer, C. Dolle, D. Dasler, G. Abellán, F. Hauke, A. Hirsch and E. Spiecker, *Nat Commun*, 2020, **11**, 1743.

8 F. Ren, C. R. Abernathy, S. J. Pearton and J. R. Lothian, *Journal of Vacuum Science & Technology B: Microelectronics and Nanometer Structures Processing, Measurement, and Phenomena*, 1995, **13**, 293–296.

9 D. B. Suyatin, C. Thelander, M. T. Björk, I. Maximov and L. Samuelson, *Nanotechnology*, 2007, **18**, 105307.

10 C. Lattyak, K. Gehrke and M. Vehse, *J. Phys. Chem. C*, 2022, **126**, 13929–13935.

11 M. D. Siao, W. C. Shen, R. S. Chen, Z. W. Chang, M. C. Shih, Y. P. Chiu and C.-M. Cheng, *Nat Commun*, 2018, **9**, 1442.
