# Supplementary material for: Visualizing nanoscale charge flows in multi-dimensional WSe2/GaAs vertical diodes
Source: Commun Mater. 2026 Jun 13;7(1):294. doi: 10.1038/s43246-026-01234-6 (PMC13727687; doi:10.1038/s43246-026-01234-6)
Supplement: Supplementary file 2 [file 43246_2026_1234_MOESM2_ESM.pdf]

Supplementary Information

Visualizing Nanoscale Charge Flows in Multi-Dimensional WSe_2/GaAs Vertical Diodes

Michele Zendrini[1], Claire Blaga[1], Matilde Cantele[1], Kamil Artur Wodzislawski[1,2], Alok Rudra[1,2], Mitali Banerjee[3], Anna Fontcuberta i Morral[1,4], Valerio Piazza[1]

[1] Laboratory of Semiconductor Materials, Institute of Materials, Ecole Polytechnique Fédérale de Lausanne (EPFL), 1015 Lausanne, Switzerland

[2] Epitaxy Core Facility, Ecole Polytechnique Fédérale de Lausanne (EPFL), 1015 Lausanne, Switzerland

[3] Laboratory of Quantum Physics, Topology and Correlations, Institute of Physics, Ecole Polytechnique Fédérale de Lausanne (EPFL), 1015 Lausanne, Switzerland

[4] Institute of Physics, Ecole Polytechnique Fédérale de Lausanne (EPFL), 1015 Lausanne, Switzerland

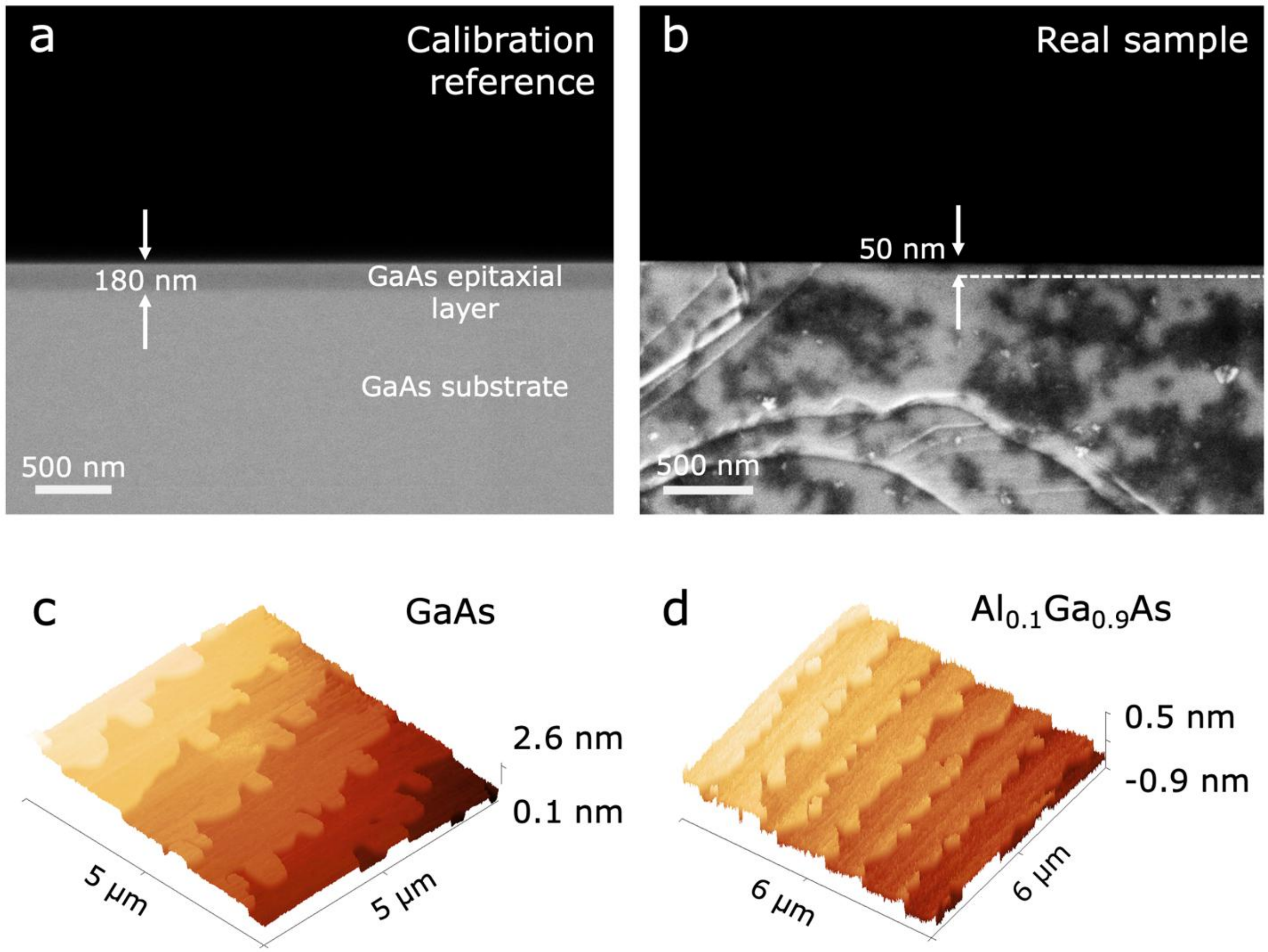

Supplementary Figure 1: *(a) SEM cross section of the calibration growth for the epitaxial GaAs layer on Zn-doped GaAs (100) substrates; (b) SEM cross section of the final epitaxial layer; 3D AFM maps of epitaxially grown GaAs (c) and AlGaAs (d) layers, showing atomic terraces on the surface.*

Planar epitaxial substrates for integration of WSe_2 flakes are grown by MOVPE as detailed in the Methods.

Supplementary Figure 1a shows a cross-section SEM image used for calibrating fluxes and growth time. The SE contrast arising from the difference in doping from the substrate (Zn-doped GaAs) and the intrinsic epitaxial layer allows to measure a thickness of the grown layer around 180 nm. Supplementary Figure 1b shows the cross-section SEM of a thinner layer used for integration. The visible pale contrast allows to measure a layer thickness close to 50 nm.

The surface morphology of the epitaxial layer needs to be atomically smooth to ensure a pristine interface with the WSe_2 flakes. Supplementary Figure 1c shows the 3D AFM map of the epitaxially grown layer, highlighting atomically flat terraces with sub-nm roughness. In a similar manner, Supplementary Figure 1d shows the surface quality of $Al_xGa_{1-x}As$ layers described in the next sections of this Supporting Information document. As for GaAs, $Al_xGa_{1-x}As$ epilayers grow with atomically flat surfaces.

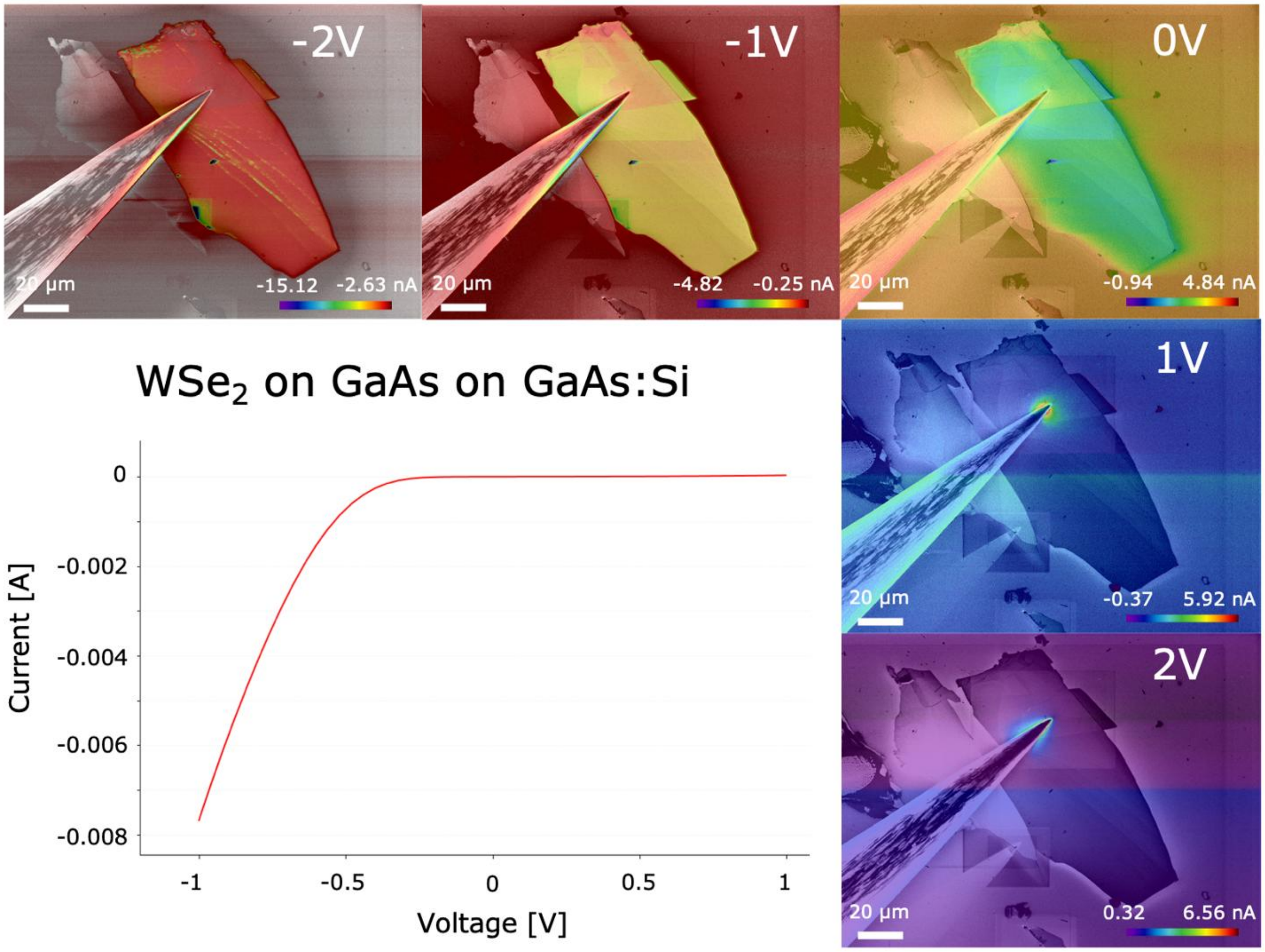

Supplementary Figure 2*: Voltage series of EBIC maps for WSe_2 flake on top on GaAs:Si substrate which is expected to create a n-n junction. Consistently the micrographs show no evident sign of current collection from the heterojunction. IV curve obtained upon contacting shows diode-like behavior at negative bias due to the top Schottky contact with the tip. EBIC maps have been acquired under electron beam with 2 keV acceleration voltage and ~ 0.4 nA probe current)*

Supplementary Figure 2 describes in more detail the electrical measurements (EBIC mapping and IV curve) on WSe_2 on epi-GaAs grown on GaAs:Si. As described in the Methods section, all the measurements are carried out connecting the positive pole to the GaAs substrate and the negative pole to the tungsten tip.

IV curve shows a rectifying behavior thus indicating the presence of electrical diode in the circuit. The polarity of the diode is consistent with p-doping in the WSe_2 component or a Schottky contact at the n-WSe_2/tungeste interface. EBIC maps under reverse bias show no relevant signal. A homogenous current is recorded on WSe2 as well as on the tungsten tip, indicating that this signal originates from absorption of the primary beam or electron beam absorbed current signal (EBAC). Electrical maps under forward bias reveal the origin of the rectifying element observed in the IV curve. In fact, an intense signal is collected on a very narrow region around the tip, compatible with a Schottky contact. The measurement therefore indicates that WSe_2 is electron-rich.

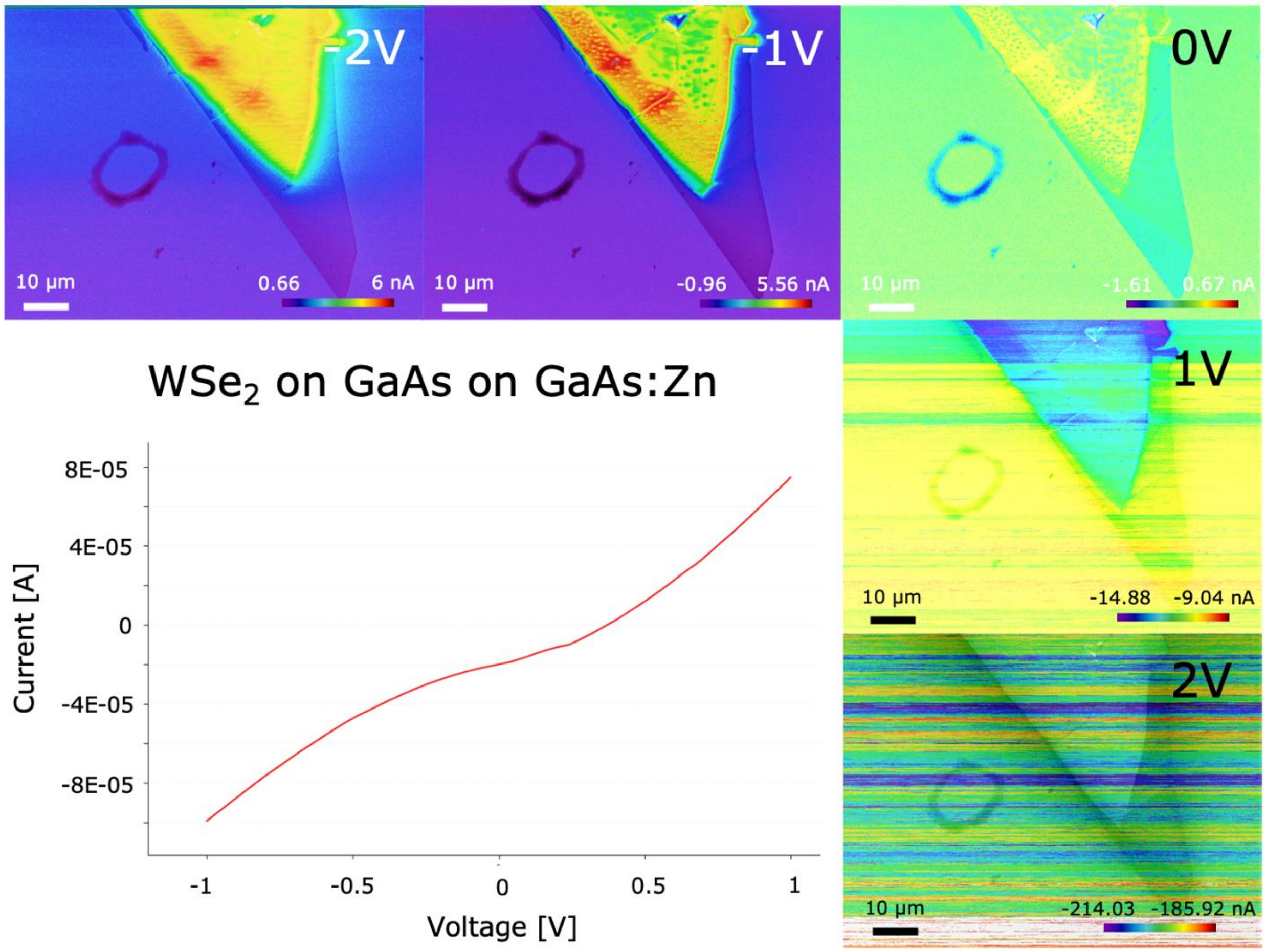

Supplementary Figure 3*: Voltage series of EBIC maps for WSe2 flake on top on GaAs:Zn substrate. The same flake is reported in the main manuscript. EBIC maps show current collection from the heterojunction. IV curve obtained upon contacting shows rectification behaviour thanks to the presence of a built-in field at the heterojunction. EBIC maps have been acquired under electron beam with 2 keV acceleration voltage and ~ 0.4 nA probe current)*

Supplementary Figure 2 describes in more detail the electrical measurements (EBIC mapping and IV curve) on WSe_2 on epi-GaAs grown on GaAs:Si. As described in the Methods section, all the measurements are carried out connecting the positive pole to the GaAs substrate and the negative pole to the tungsten tip.

IV curve shows a rectifying behavior thus indicating the presence of electrical diode in the circuit. The polarity of the diode is consistent with p-doping in the WSe_2 component or a Schottky contact at the n-WSe_2/tungeste interface. EBIC maps under reverse bias show no relevant signal. A homogenous current is recorded on WSe2 as well as on the tungsten tip, indicating that this signal originates from absorption of the primary beam or electron beam absorbed current signal (EBAC). Electrical maps under forward bias reveal the origin of the rectifying element observed in the IV curve. In fact, an intense signal is collected on a very narrow region around the tip, compatible with a Schottky contact. The measurement therefore indicates that WSe_2 is electron-rich.

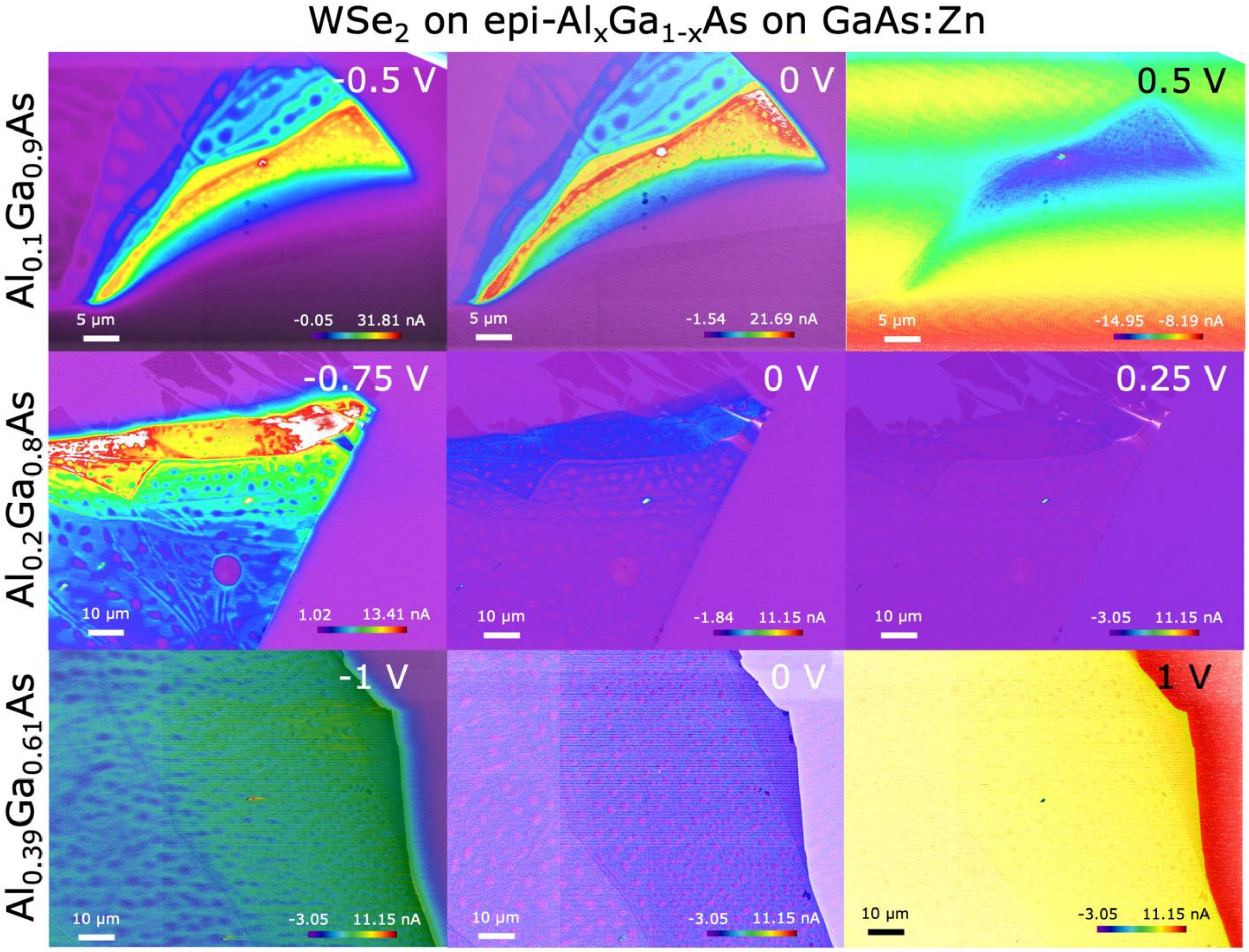

Supplementary Figure 4*: EBIC maps of different heterojunctions between WSe_2 and $Al_xGa_{1-x}As$ epilayers at different Al concentration (10%, 20%, 39%, on the rows) at different external biases. EBIC maps have been acquired under electron beam with 2 keV acceleration voltage and ~ 0.4 nA probe current)*

The electrical behavior of the $WSe_2/Al_xGa_{1-x}As$ heterojunctions is shown in Supplementary Figure 4. The maps show a consistent trend with what has been described in the main manuscript for WSe_2/GaAs:Zn heterojunctions, where the presence of the built-in field is driving the current extraction. Equivalent behaviors at different Al concentrations demonstrate how the built-in field is not significantly affected by the band offsets at the junction.

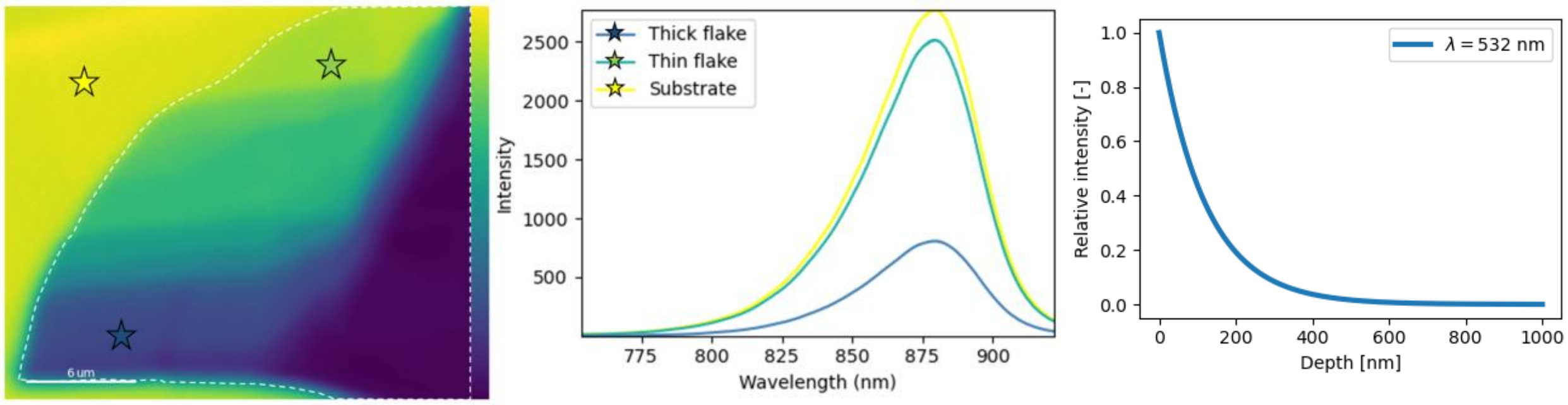

Supplementary Figure 5: *Photoluminescence (PL) panchromatic map (top left) of a multilayered WSe2 flake (outlined in dashed white line) on GaAs epilayer (integrated in the range 750 nm – 925 nm). Selected PL spectra (top right) taken on the substrate, on the thin and thick parts of the* WSe_2 *flake. Decay of 532 nm laser intensity (bottom) with penetration depth in bulk GaAs. Absorption coefficient taken from [1].*

In the panchromatic PL map, WSe_2 emission, which would appear as bright areas, is not visible. This indicates that the minimum flake thickness is over two layers (thicknesses at which WSe_2 signal would be visible with the acquisition conditions used). The PL emission comes solely from the GaAs substrate and is partially to totally quenched below the flake, as shown by the decrease in intensity of the PL emission peaks taken beneath the flake. This is due to the latter's laser screening effect. Indeed, penetration in GaAs of the 532 nm laser used for the measurements is well above the 80 nm maximum thickness of the flake.

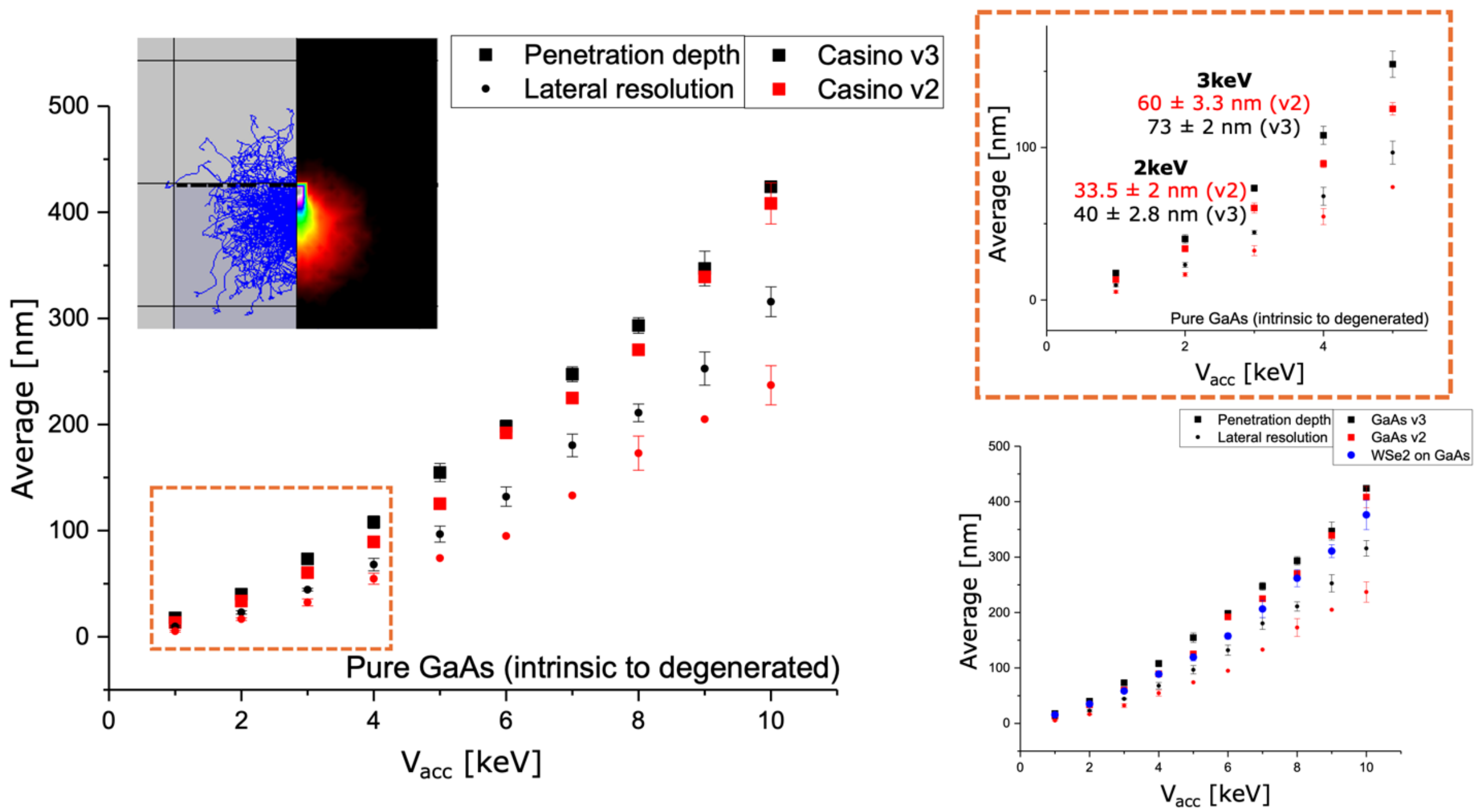

Supplementary Figure 6: *MonteCarlo simulations of penetration depth of the interaction volume at different acceleration voltages in GaAs. Labels v2 and v3 refer to 2-dimensional and 3-dimensional modeling, showing comparable results with minor differences. Error bars refer to values obtained for different simulations at different doping of the substrate: intrinsic, n-doped at 10^{17}, and n-doped at 10^{19}. Orange inset plot shows zoomed-in version of the major plot with reported values of penetration depth for the acceleration voltages used in our experimental investigations. Bottom right plot overlaps additional simulation results for intrinsic GaAs with overlayer of WSe_2, with error bars referring to different simulated thicknesses (from 5 to 50 nm).*

MonteCarlo simulations with Casino software on pure GaAs. The plot summarizes penetration depth and lateral resolution as a function of primary beam energy. The error bar refers to varying electron concentration (intrinsic, 10^{17} cm^{-3}, 10^{19} cm^{-3}). Labels v2 and v3 denotes 2D and 3D modeling. Values are extracted by applying a 5% energy cut. The inset in the dashed orange square highlights the relevant values for the experimental conditions of the maps reported in this work. The plot on the right includes values for WSe_2 on intrinsic GaAs (blue points). The error bar refers to WSe_2 thickness (from 5nm to 50 nm).

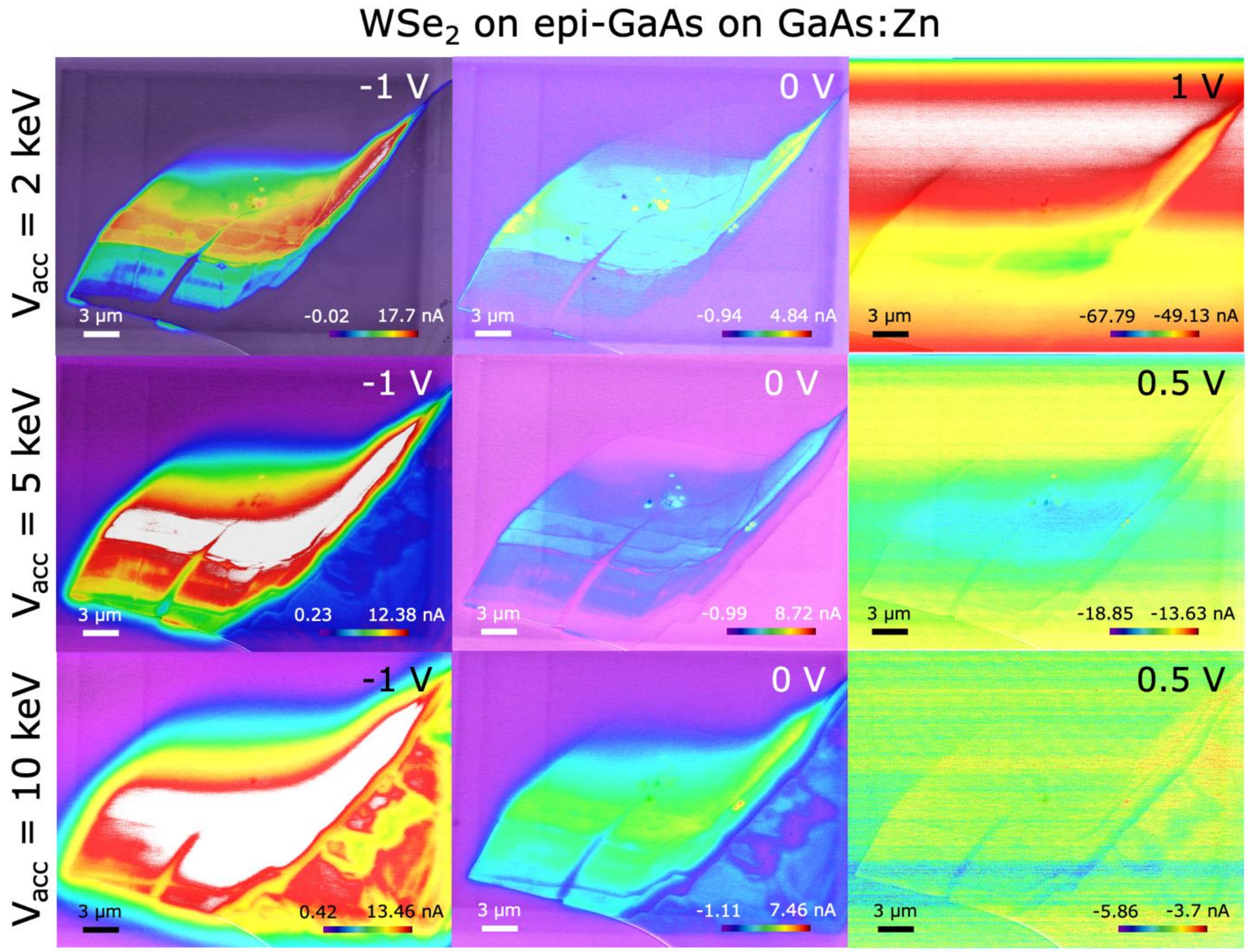

***Supplementary Figure** 7: EBIC maps of WSe2/GaAs:Zn heterojunction at different acceleration voltages (2, 5, and 10 keV, on the rows) at different external biases. EBIC maps have been acquired under electron beam with 2 keV acceleration voltage and ~ 0.4 nA probe current)*

Supplementary Figure 7 shows the EBIC maps obtained for the WSe_2/GaAs:Zn heterojunction at different external biases for different acceleration voltages. The external bias evolution is not affected by the acceleration voltage, qualitatively remaining the same with morphological features reproduced by current profiles in reverse bias. With increasing V_{acc}, thus penetrating deeper in the substrate, the current collection is extended towards thicker regions (see left column evolution with increasing V_{acc}). This observation is fully consistent with Casino simulations, discussed in the previous paragraph of this Supporting Information.

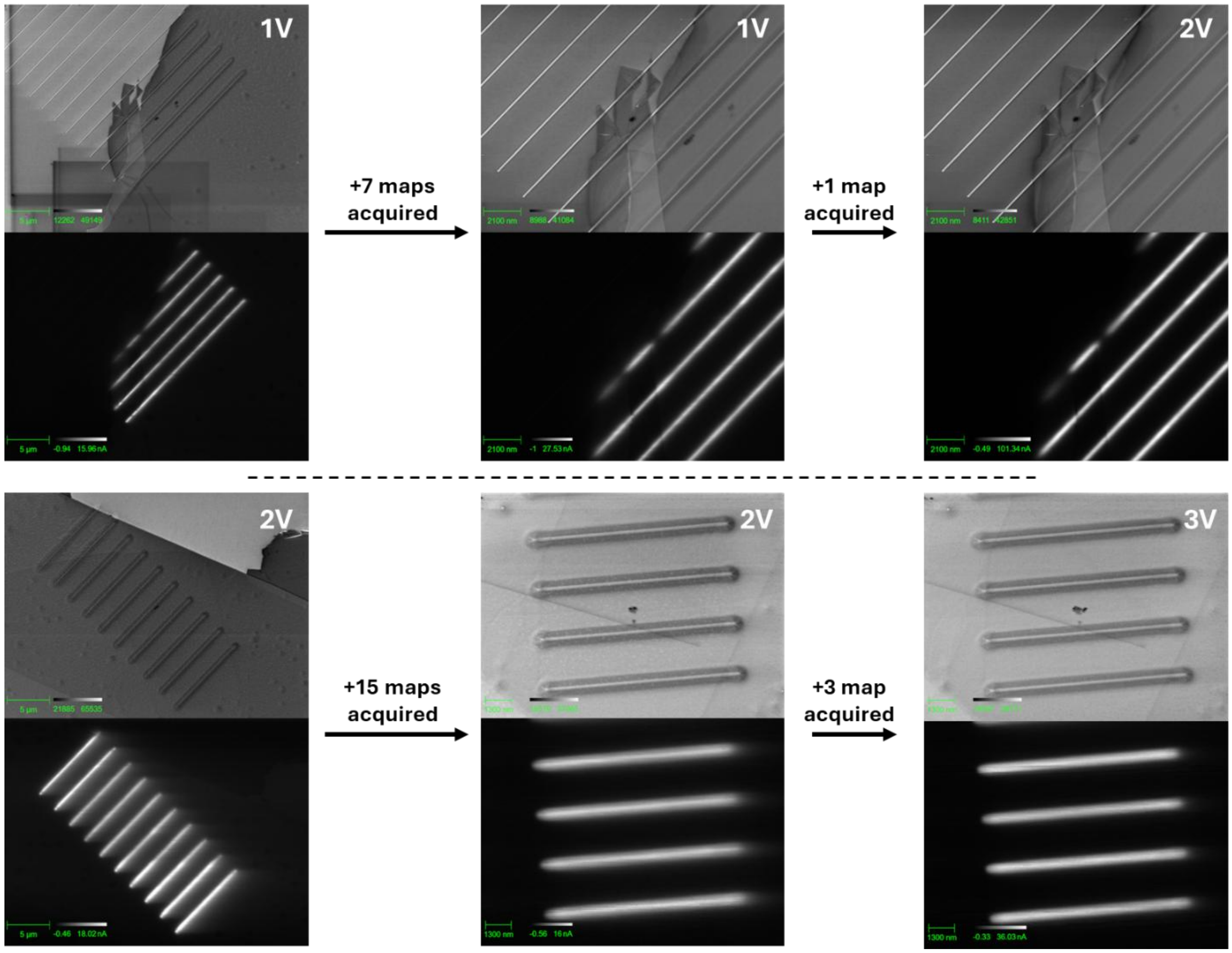

Supplementary Figure 8*: SEM images and EBIC maps after repeated exposure. The top and the bottom panels report two distinct WSe_2 flakes on GaAs nanostructures. Both the top and the bottom panel display an early map (left), a map under the same bias after several acquisitions (center) and the last recording of the session (right)- commonly at a different bias. All the maps are acquired at 3 keV and probe current around 0.4 nA.*

Supplementary Figure 7 shows the stability of EBIC maps after repeated exposures. Two examples are displayed where the map at the beginning of the measurement session (left) is compared to an equivalent map acquired towards the end of the measurement session under the same conditions (center) and under different bias (right). Maps do not display signs of electron beam induced damage thus validating the electron beam parameters used for this study. The top example shows a current increase after multiple measurements. This behavior cannot be generalized. For example, the bottom panel displays a lower induced current after repeated exposure. It is important to note that the current collected depends on the contact resistance which in turn varies with the applied pressure. Tip position is adjusted multiple times during measurements to counteract probe drift thus the absolute value of extracted current is hardly comparable amongst different scans.

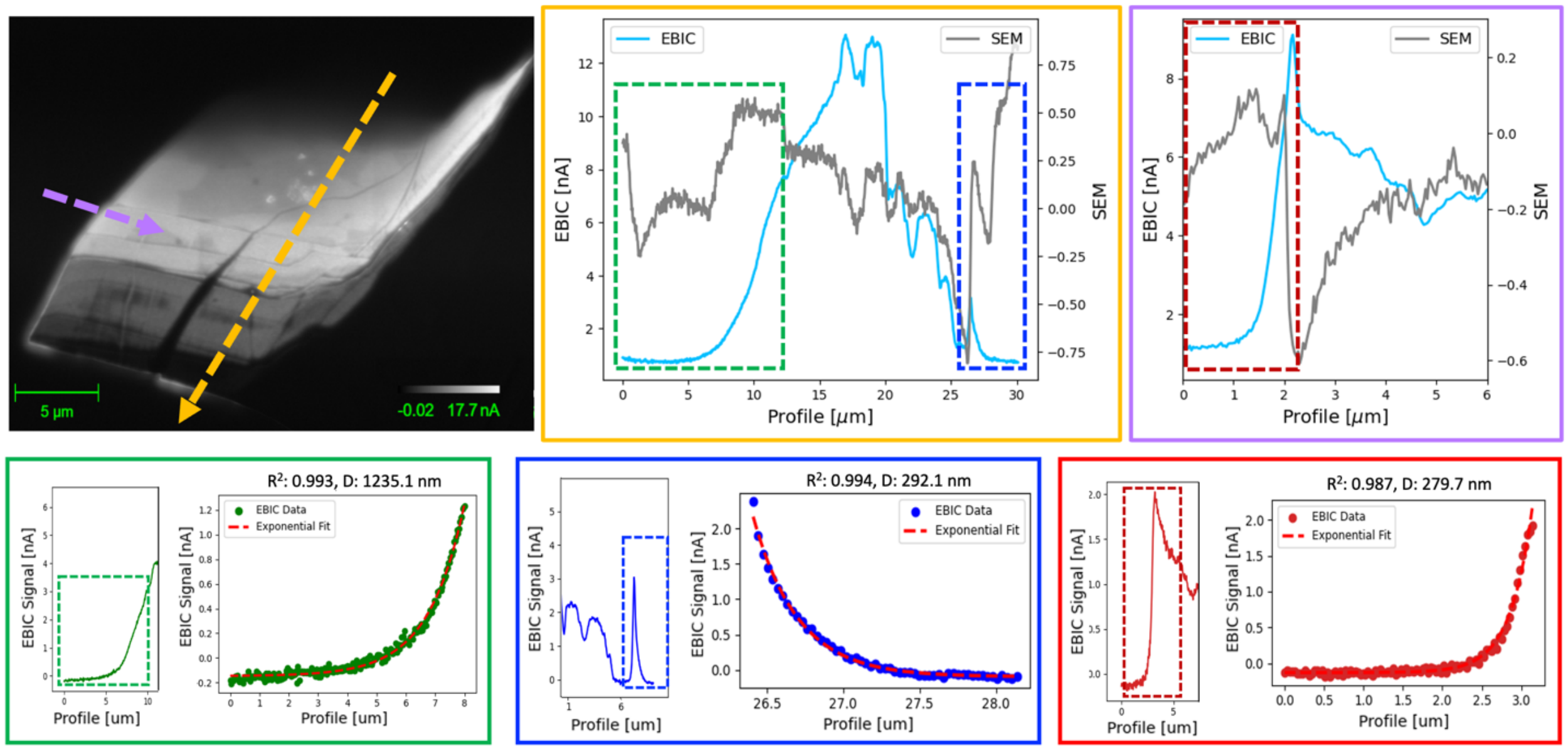

Supplementary Figure 9*: Diffusion length analysis for 2D/3D WSe2 on III-V heterojunctions. At the top left, EBIC map of a WSe2/epi-GaAs/GaAs:Zn heterostructure with colored arrows representing the two major directions of current profiles extraction for diffusion length estimation: across terraces (yellow) and along terraces (violet). At the top right, superimposed EBIC and SEM profiles extracted along yellow and violet lines, showing correspondence between contrast (morphological) features and current trends. Dashed rectangles refer to fit regions for the curves shown at the bottom, where examples of current profiles are shown, together with the extracted fit parameters.*

The diffusion length data extraction and analysis on the 2D/3D WSe_2/epi-GaAs/GaAs:Zn heterostructures have been performed by manually tracing sets of current profiles on the EBIC maps. These sets are shown in Supplementary Figure 9 with arrows of different colors, representing the heterojunction border at different WSe_2 thicknesses (purple), and the heterojunction profile with varying WSe_2 thickness (yellow). Extracted current profiles are shown in the plots on top, together with dashed insets representing the fitted regions. Profiles as in the green inset provide insight on the hole diffusion length in WSe_2, being the portion of the flake where drift is not predominant. Profiles in blue provide equivalent information but on a single thickness at the bottom of the flake to account for reliability of the data extraction. The current profiles at the border of the 2D material (red bottom plot) allow for the extraction of GaAs diffusion lengths, from far away from the flake towards the interface.

Diffusion lengths are obtained by selection of portions of the current profiles to be fitted. Cross-correlation of SEM and EBIC signal defines fit boundaries. Data are fitted with exponential profiles, consistently with theoretical expectations from diffusion regimes. R-squared values of the fits are reported here for the examples, generally resulting larger than 0.98 and suggesting reliable match between experimental data and extracted parameters.

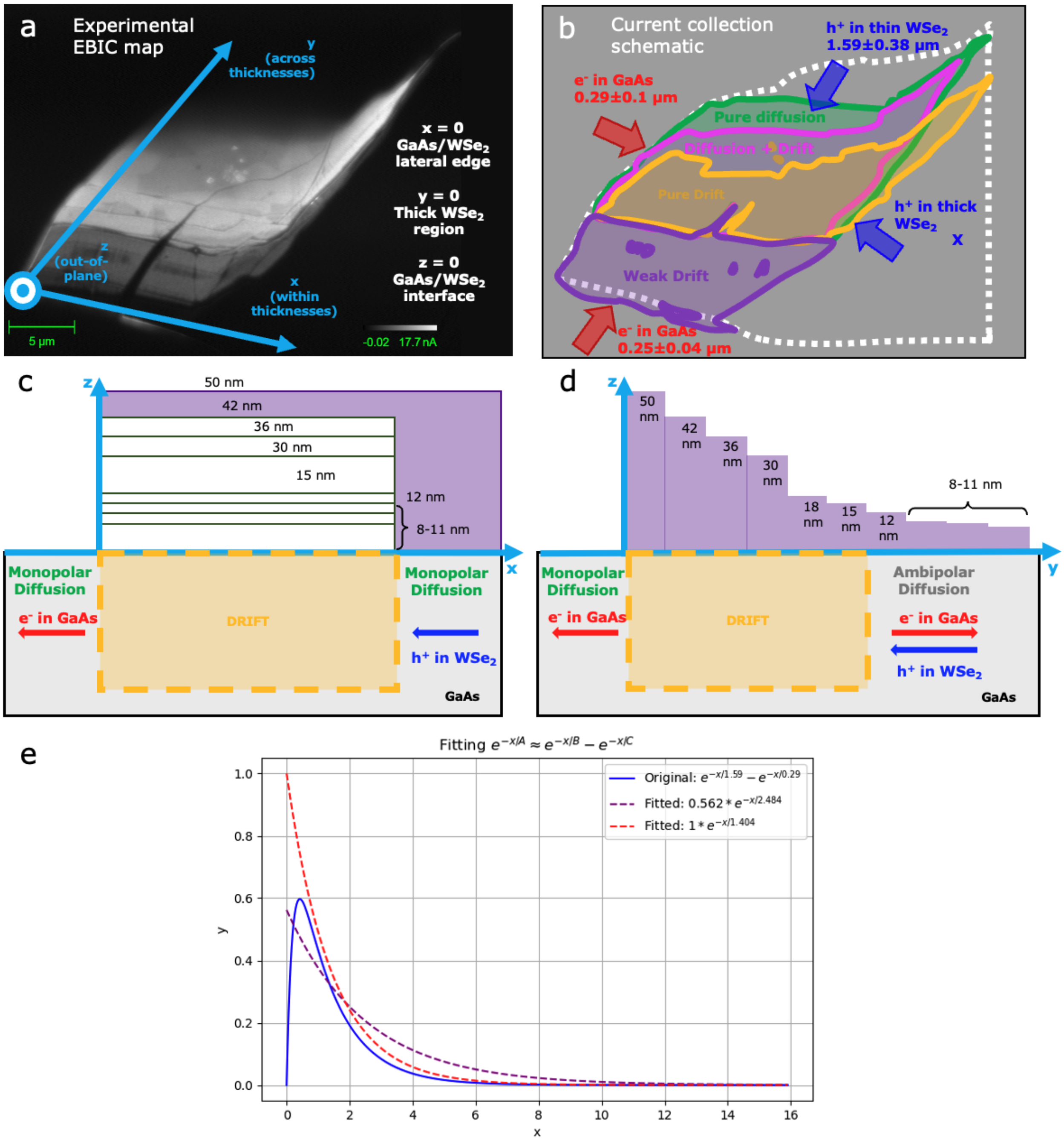

Supplementary Figure 10: *(a) EBIC experimental map of WSe_2/GaAs:Zn heterojunction (b) Current collection schematic for different WSe_2 thickness regions; (c) Cross-sectional diagram along a cut on the x-z plane, showing two monopolar regimes for holes diffusion in WSe_2 and electron diffusion in GaAs; (d) Cross-sectional diagram along a cut on the y-z plane, showing ambipolar diffusion in thin regions of the WSe_2 flake. (e) Plots of ambipolar diffusion mechanism for the separate regime (blue line) and the fitted ones with estimated values of effective diffusion lengths with (purple dashed) and without (red dashed) pre-exponential fitting. EBIC maps have been acquired under electron beam with 2 keV acceleration voltage and ~ 0.4 nA probe current)*

Supplementary Figure 10 shows a series of diagrams for the diffusion analysis of the 2D/3D WSe_2/GaAs:Zn heterojunction. The set of coordinates on the EBIC experimental map in panel a are used as reference in the schematics, while panel b shows an overlaid schematic on the equivalent thickness regions for the

current collection mechanisms, together with estimated diffusion length values for electrons in GaAs and holes in WSe_2. Panels c and d display two cross-sectional diagrams obtained from perpendicular cuts along the x and y directions of the heterojunction in our reference system.
Along x (panel c), monopolar diffusion on both sides can be described, as the interaction volume remains fully in GaAs on the left part (outside the heterojunction) and fully in WSe_2 on the right part where the flake is thick enough. These two regimes allow for the electron diffusion length extraction in GaAs, reading 290 ±101 nm (as discussed in the main manuscript). On the other side, holes diffusion length in thick WSe_2 was not quantitatively estimated due to insufficient data to extract profiles.
In a similar manner, along y (panel d), monopolar diffusion on the bottom edge can be described with interaction volume fully in GaAs. In this case, electron diffusion length in GaAs extracted from profiles results equal to 250 ±40 nm. An ambipolar diffusion regimes is found on the top edge of the flakes, where the interaction volume partially lies into thin WSe_2 and partially in GaAs. The effective diffusion length extracted from profiles as discussed in the main manuscript results equal to 1590 ±380 nm. The ambipolar diffusion regime can be described by the following Equation as in Supplementary Figure 10e:

$$J = A * exp\left(-\frac{x}{L_{diff}^{eff}}\right) = B * exp\left(-\frac{x}{L_{diff}^{GaAs}}\right) + C * exp\left(-\frac{x}{L_{diff}^{WSe2}}\right)$$

If A ≈ B ≈ C, one can rewrite:

$$exp\left(-\frac{x}{L_{diff}^{WSe2}}\right) = exp\left(-\frac{x}{L_{diff}^{eff}}\right) - exp\left(-\frac{x}{L_{diff}^{GaAs}}\right)$$

where L_{diff}^{eff} is defined by the fitting and L_{diff}^{GaAs} can be deduced by the previous analysis. By using L_{diff}^{eff} = 1.59±0.38 μm and L_{diff}^{GaAs} = 0.29±0.1 μm, we find L_{diff}^{WSe2} = ~ 1.5 μm, (dashed red curve).

If one assumes B ≈ C instead:

$$\frac{A}{B} * exp\left(-\frac{x}{L_{diff}^{WSe2}}\right) = exp\left(-\frac{x}{L_{diff}^{eff}}\right) - exp\left(-\frac{x}{L_{diff}^{GaAs}}\right)$$

A double parameter fitting returns L_{diff}^{WSe2} = ~ 2.5 μm (purple dashed curve).

Although the first assumption offers a better fit, we conservatively indicate a rough value of $L_{diff}^{WSe2} \sim$ 2 μm in the main text.

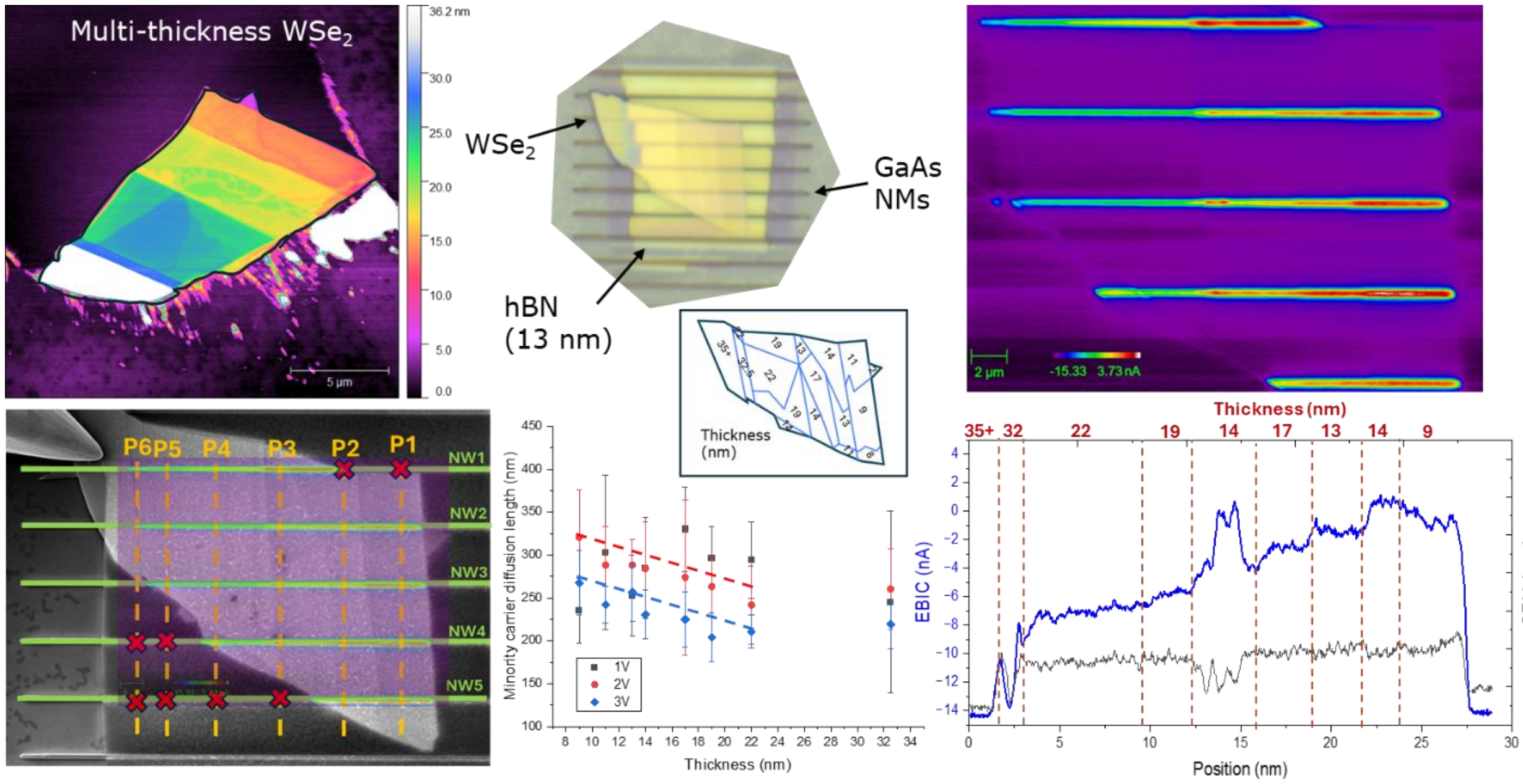

Supplementary Figure 11: Characterization and EBIC measurements on an encapsulated flake. AFM micrograph of the multi-thickness WSe_2 flake (top, left). Optical image of the encapsulated heterostructure (top,center). EBIC map under 2keV electron beam, ~ 0.4 nA probe current and external bias of 3V. Other probing and acquisition parameters are the same described in the Methods section (top, right). Visual summary of the system obtained by overlaying SEM and EBIC micrographs. For the analysis we extracted representative longitudinal profiles along GaAs nanostructures (highlighted in green) and transversal profiles (dashed orange lines). The red crosses indicate points along the transversal profile where WSe_2 and GaAs do not touch (bottom left). Hole diffusion length in WSe_2 - extracted by transversal EBIC profiles – as a function of the layer thickness. The inset reports the mosaic of thicknesses identified by AFM (bottom, central). Longitudinal SEM (black) and EBIC (blue) profile along the central NW. The dashed lines separate terraces with different thickness. The thickness of each terrasse is indicated with the corresponding value in red.

To demonstrate the role of surface contamination on carrier transport, we fabricated a WSe_2/GaAs heterostructure encapsulated with a hBN flake (on top) approximately 13 nm thick as measured by AFM. GaAs nanostructures used as a substrate (grown on (111)B substrate) are fabricated as described in the main manuscript. The top panels of Supplementary Figure 11 display, respectively, the AFM micrograph of the bare WSe_2 flake, the optical image of the stack and the corresponding EBIC map. As described in the main manuscript, the latter exhibits electrical signal only along the nanostructure.

The bottom left panel reports overlaid SE and EBIC images thus displaying the 2D stack on GaAs nanostructure and the corresponding electrical signal. In the top-left corner, one can see that the tungsten tip is in contact with the thick part of the flake (> 35nm), intentionally left uncovered by hBN to enable electrical access. Overall, the map exhibits no major difference with respect to measurements on non-encapsulated samples except for a degradation of the signal-to-noise ratio due to the presence of the top insulating layer. We therefore analyzed the results by extracting longitudinal profiles along the 5 nanostructures covered in WSe_2 (highlighted in green in the bottom left panel) and by extracting arbitrary transversal profiles in the orthogonal direction with respect to the elongated nanocrystals (in orange in the bottom left panel).

The combination of these line profiles allows investigation diffusion length and electrical signal intensity variation on a large number of terraces with different thicknesses (identified by AFM as reported in the central inset of Supplementary Figure 11). Transversal profiles were fitted with the same method as

discussed in the main manuscript thus extracting hole diffusion length in WSe_2. The results reported in the plot in the bottom central panel report values extracted under bias of 1V, 2V and 3V. Even though the hole diffusion length extracted at 1V has similar values to those observed at 2V and 3V, the poor signal to noise ratio hinders accurate analysis (fitting R2 at 1V below 0.85). Under larger external bias, the diffusion length values agree with the estimate found for bare planar heterostructures (around 300nm). It must be noted that here we observe an unexpected slight variation of the minority carrier diffusion length with external bias (around 320 nm at 2V, around 270 nm at 3V). This may indicate a drift component slightly extending laterally. At the same time, this observation goes beyond the purpose of the measurement, thus it is not investigated further. Interestingly the extracted hole diffusion length values are found to decrease with thickness as reported for the non-encapsulated sample (dashed blue and red line in Supplementary Figure 11). However, the relative variation is lower after hBN encapsulation.

Longitudinal profiles instead reveal a significant difference with respect to the case of uncovered WSe_2. The bottom right panel of Supplementary Figure 11 reports the current profile along the central nanostructures as a representative case. In the case of heterostructure covered with hBN no diffusive regime is observed despite the presence of three large terraces below the threshold value of 15 nm, identified in the main manuscript. Since under these experimental conditions EBIC signal is entirely drift-dominated it is possible to correlate the extracted current with flake thickness. The attribution of current-thickness relationship appears extremely robust since two distinct terraces with the same thickness (14 nm) exhibit the same current level. Interestingly the current-thickness relationship is not monotonic for thin terraces below the threshold value as current level slightly decreases for terraces thinner than 14 nm. This may indicate residual contamination which still acts to weaken the built-in field. Another possible explanation may be related to a limited extension of the depletion region in GaAs: in this case, the lower current would represent a suboptimal overlap between the space charge region and the interaction volume as for thick terraces.

Altogether this measurement suggests that hBN encapsulation has a significant impact on carrier transport even if it does not completely prevent thickness-dependent carrier distribution. Although this measurement suggests that the electric field may still decrease with thickness in encapsulated samples, the physical description appears strongly modified. Altogether these additional data reinforce the hypothesis that the underlying mechanism is dominated by surface contaminations which are, at the very least, significantly mitigated by hBN encapsulation with respect to the case of non-encapsulated samples.

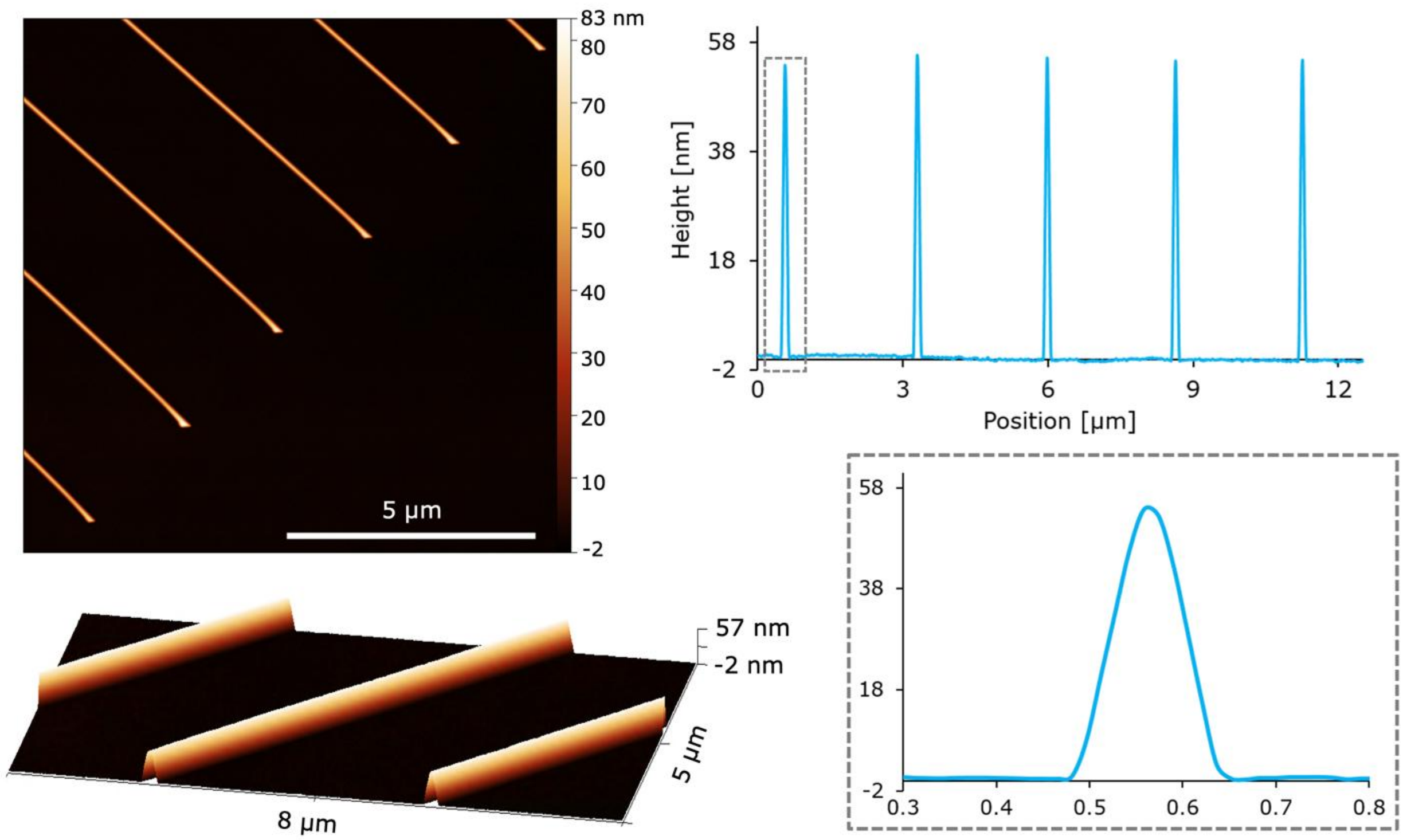

Supplementary Figure 12: *Morphological characterization of GaAs (100) NMs. On the left, 2D and 3D AFM maps of 1D horizontal NMs, showing perfect selectivity on the SiO2 mask and reproducible uniformity over the array. On the right, line profiles from the AFM data traced perpendicularly to NMs, highlighting clear homogeneity in height for different structures (55 nm on average). Width of NMs is nominally patterned at 60 nm, but after growth they laterally expand up to 230 nm on average.*

Nanostructured substrates are carefully characterized by SEM and AFM to evaluate the nanostructures' quality and dimensions. Supplementary Figure 12 shows the AFM analysis that is generally performed on the substrates prior to integration. The 2D AFM map confirms the absolute selectivity of the epitaxial growth on the specific array for integration, with flawless NMs and no trace of parasitic growth. This aspect is also generally assessed and verified by SEM on larger scale on the whole growth sample. The 3D AFM outline shows the morphology of NMs growing on GaAs (100) substrates. As demonstrated in detail in previous studies [2,3], NMs evolve outside the SiO2 mask with a pyramidal cross-section, truncated at medium growth stages, towards completion at final ones, as in Supplementary Figure 12 and in general in the substrates we used for integrations. The AFM profiles highlight the ideal reproducibility of the NMs within a single array, with height deviations of ±1 nm for heights of tens of nanometers. Finally, the inset profile at the bottom shows once again the cross-sectional profile of the NMs more in detail, evidencing the lateral size of the structures being as large as 200 nm. The nominally patterned width of the NMs, equal to 60 nm in our case, enlarges not only due to slight electron beam overexposure, but also because of natural growth mode of these nanostructures. Lateral overgrowth over the mask happens at first growth stages, while progressive cross-sectional expansion occurs upon completion of the pyramid [2].

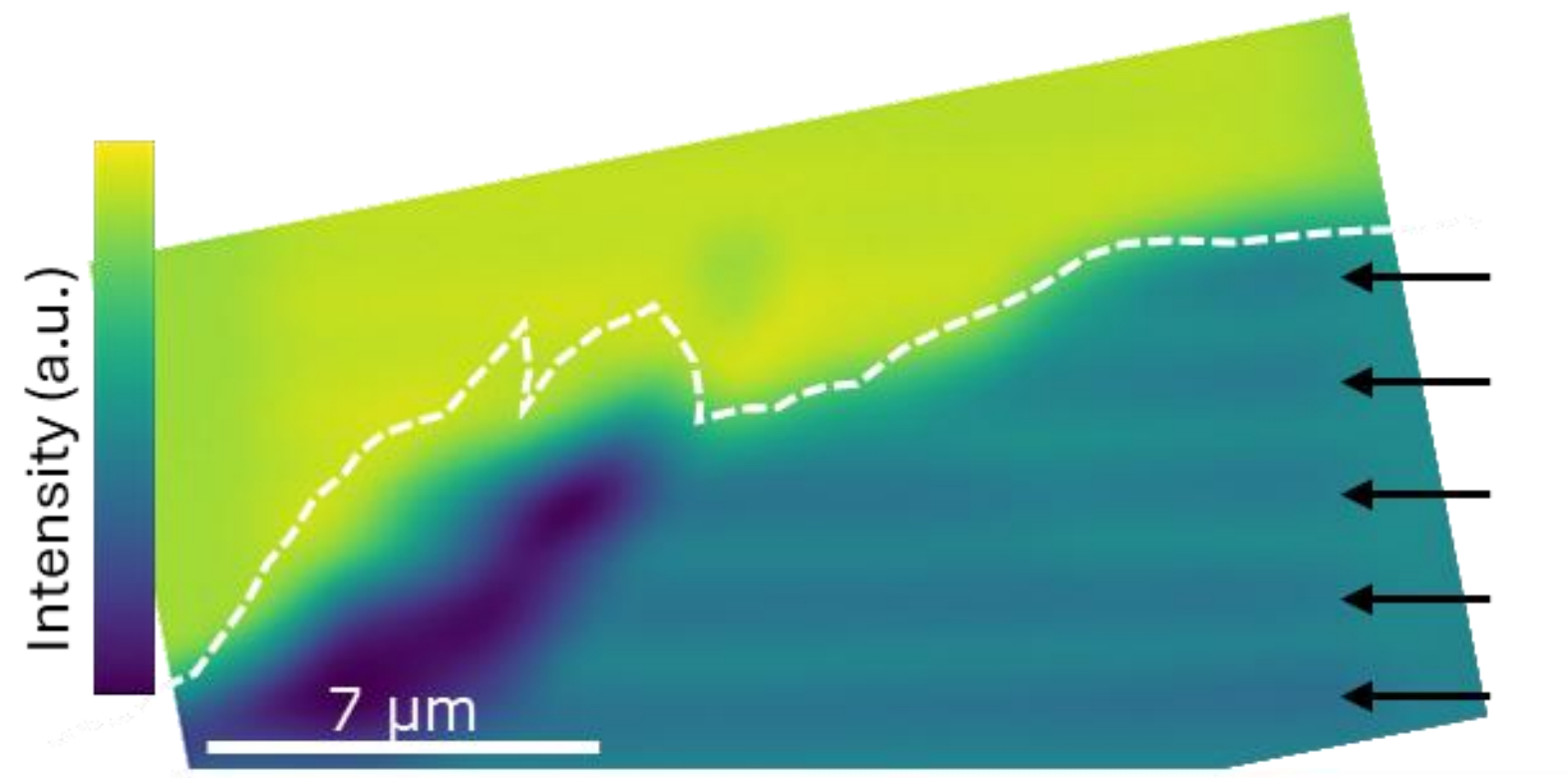

Supplementary Figure 13*: PL map of the WSe_2 flake (outlined in dashed white line) transferred on GaAs NMs, integrated in the GaAs emission range 850 nm – 900 nm.*

Supplementary Figure 13 shows intensity map, indicated by the colorbar of the integrated PL emission of GaAs over the range 850 nm – 900 nm. The sample is a WSe_2 flake, outlined in a dashed white line, on GaAs NMs whose position is indicated by the lateral arrows. The NMs appear as bright horizontal lines outside of the flake boundary and as dark horizontal lines within the flake boundary. It can be seen that the GaAs emission intensity is reduced by the presence of the flake (area under the white line), with thicker regions of the flake appearing darker. This is due to the WSe_2 flake screening the laser intensity and thus lowering the excitation power on the substrate below. Furthermore, outside of the flake region (above the white line), it can be observed that the bare NMs have a stronger emission than the surrounding substrate. However, the NMs covered by the WSe_2 show lower emission compared to the surrounding substrate under the flake. This indicates a change in the recombination process of the photogenerated charge carriers confined to the areas where the GaAs NMs are in contact with the WSe_2. In these 1D heterojunction regions, the presence of a built-in-field leads to charge separation of the photogenerated carriers and therefore, partial quenching of the photoluminescence. This phenomenon is absent in the surroundings of the NMs due to the presence of the SiO_2 mask.

.

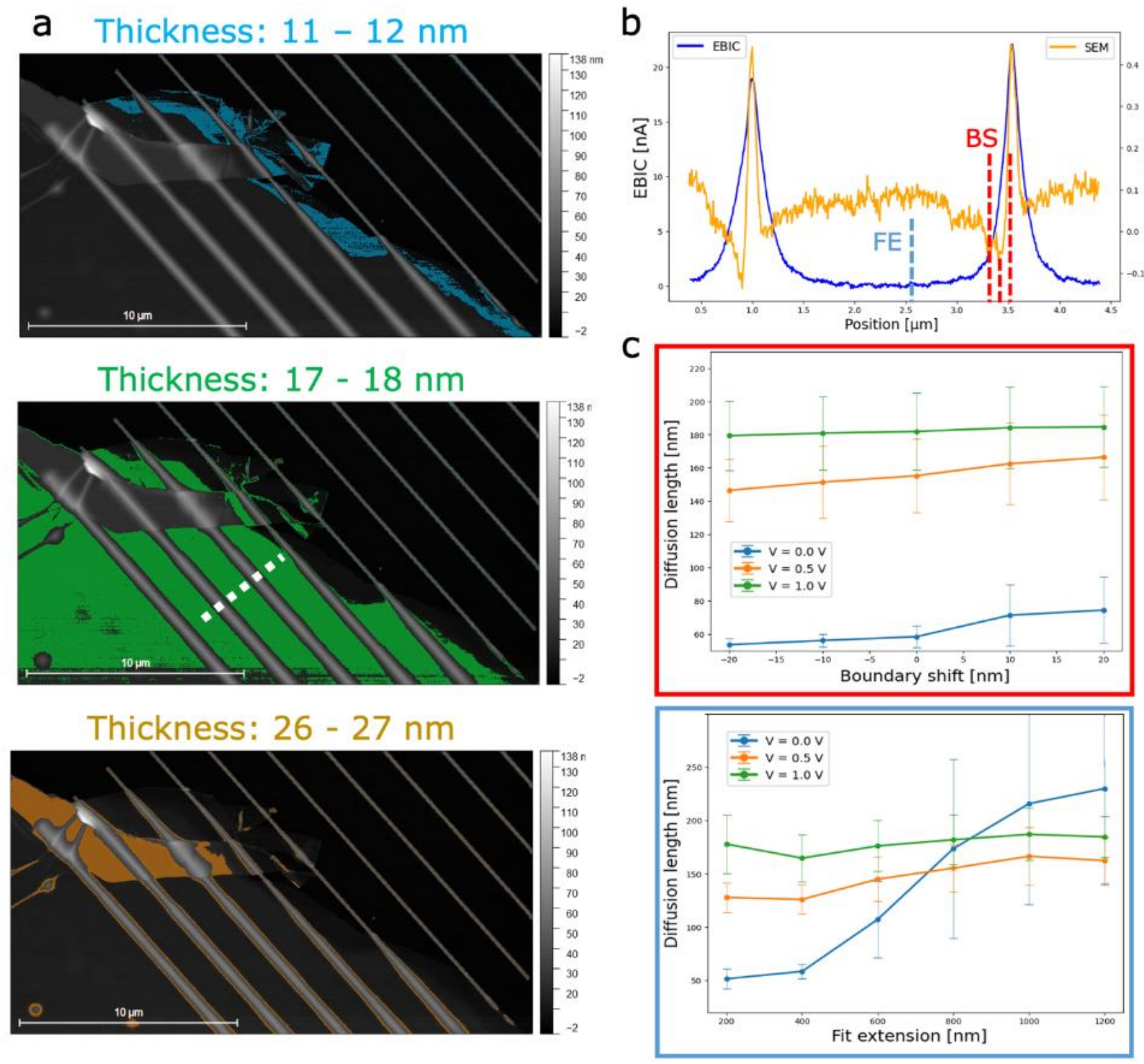

Supplementary Figure 14*: Diffusion length analysis for 2D/1D TMDs/III-V heterojunctions. (a) AFM maps with different height mask for flake thicknesses identification; (b) Current (EBIC) and contrast (SEM) superimposed profiles taken along the white line in panel (a), showing clear match between electrical and morphological features, together with a schematic of the fundamental fit parameters, namely boundary shift (BS) and fit extension (FE); (c) Diffusion length data extracted from the same fitted profile at different voltages and different boundary shifts (in red contour) and fit extensions (in blue contour), highlighting marked reproducibility at different choice of boundary parameters. Error bars are standard deviations referred to the different profiles at different positions.*

AFM data of WSe_2 on GaAs nanomembranes are masked at different height levels to define flake single-thickness regions (Supplementary Figure 14a). WSe_2 diffusion lengths for different thicknesses are estimated through current profiles thanks to well-defined interfaces between NMs and each WSe_2 thickness Supplementary Figure 14b shows the superimposed profiles of SEM contrast and EBIC signal along the white line on the central map of the panel a. Morphological-electrical correspondence evidences electrical signal only coming from regions where the 2D/1D interface is present. A Python script is hence developed to analyze the superimposition of the morphological and current profiles and define boundaries for fitting regions. Lateral borders of the NMs are identified (first two neighboring minima to the morphological maxima), defining theoretical onsets of diffusion regimes. However, pixelization and slight misalignment of the two different signals can cause mismatch of the morphological minimum with respect to the real interface borders. For this reason, a boundary shift (BS) parameter is assigned to discrete displacements of the fitting border. Similarly, a fit extension (FE) parameter is defined to account for possible variabilities when expanding or compressing the fit region. An upper limit for this parameter is set at half the pitch to avoid contributions from neighboring NMs. Supplementary Figure 14c shows the results for BS (red) and FE (blue), showing hole diffusion lengths in 17-18 nm thick WSe_2 at different voltages. The slight variation of diffusion length with BS reveals little impact of this parameter in the considered range. FE only has a significant influence at low/absent external bias (e.g blue curve), due to reduced fit accuracy in wide data ranges when decreasing signal-to-noise ratio. This discrepancy was also accompanied by a marked decrease in the fit quality parameter.

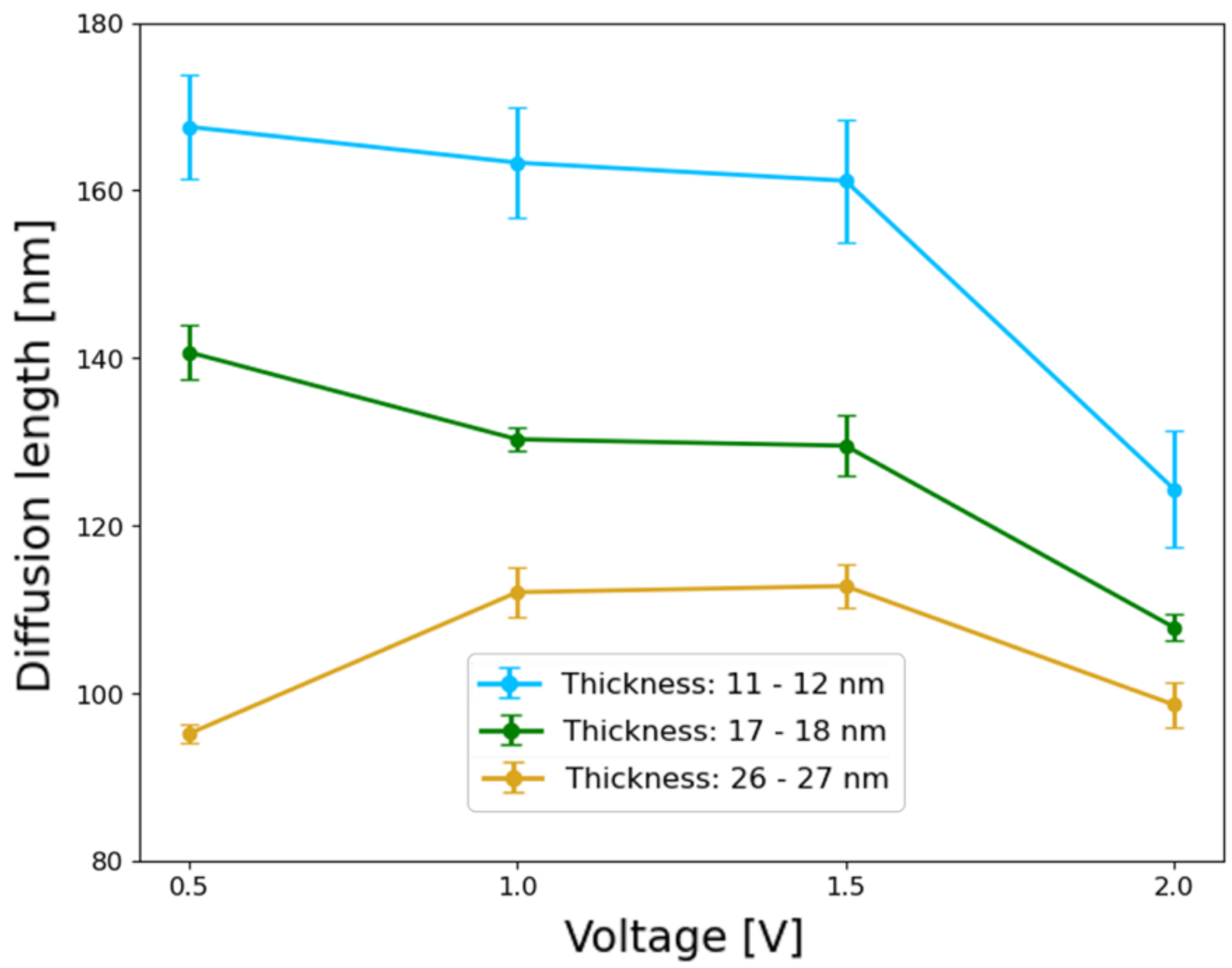

Supplementary Figure 15: *Diffusion length extracted values for different thicknesses at different voltages, showing clear thickness dependence and weak voltage dependence.*

Supplementary Figure 15 presents an example of diffusion lengths extracted for the different thicknesses under various applied voltages, for the specific integrations whose AFM maps are presented in Supplementary Figure 15. The fitting approach allowed to extract a statistically reliable set of data, considering that for each thickness several profiles are extracted, where geometrically possible thanks to the heterostructure shape. Each profile enclosed the maximum number of nanostructures covered by the 2D material at that thickness. For each covered NM in the profile, the two branches of the current profiles at the sides of the nanostructure are fitted to extract diffusion values. Diffusion length values are calculated for 5 different boundary shifts, in the range of ± 2 pixels from the identified minimum. A fit extension equal to 1 µm is chosen to include as many data as possible, apart from low voltage cases where it is reduced to 600-800 nm to increase the fit quality. All these values are averaged together to obtain one single diffusion length for each profile. All the values from different profiles at a certain thickness are averaged to obtain a single diffusion length, representative to one thickness under one specific applied bias. Error bars refer to the variability among profiles for each thickness. This analysis is repeated for each applied voltage. The same approach is consistently applied to other integrations of WSe_2 flakes on GaAs NMs to obtain the full set of comparable data. Discussion of these trends is presented in the main manuscript.

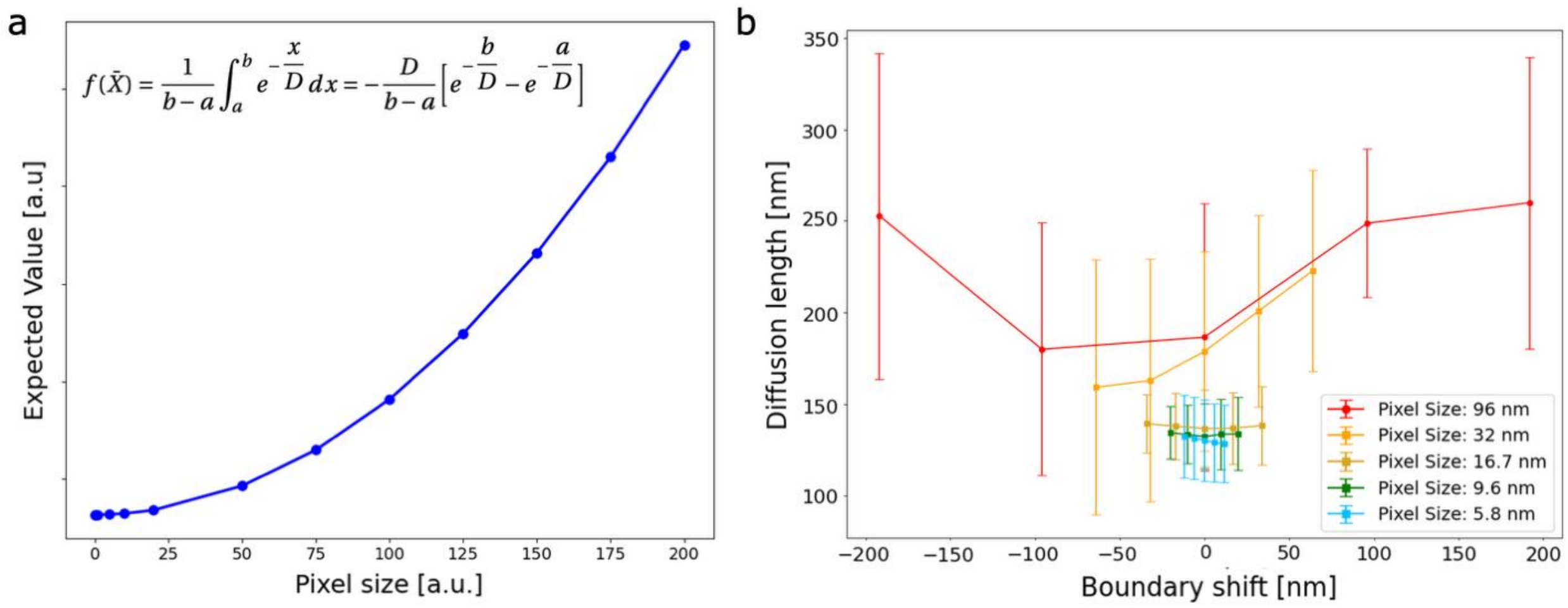

Supplementary Figure 16: *Magnification effect on diffusion length estimation. (a) Trend of the expected value of an exponential function with increasing discretization (e.g. pixel size), as described by mean value theorem in the inset equation; (b) Estimated values of diffusion length for a magnification series on a heterojunction of WSe2 on GaAs NMs at a fixed voltage and flake thickness, showing how the same profile gives pixel size dependent diffusion length values, saturating when pixel size becomes small enough.*

When extracting current profiles, the pixel size of the experimental map plays a role in determining the curve resolution, translating into possible artifacts when trying to fit the curves. The reason for this behaviour is presented in Supplementary Figure 16a: increasing the pixel size is equivalent to averaging the real current profile on larger discretized intervals of the trend. For an exponential function, this turns into larger expected values in the measured profiles than what it should be if the resolution was higher, as analytically described by the mean integral value theorem. When this happens, the characteristic length scale of fitting exponentials hence increases, as presented in Supplementary Figure 16b. Extracted diffusion lengths are plotted for acquisitions at different magnifications of the same WSe_2/GaAs-NMs heterojunction. Even if the external bias is fixed, the injection conditions are the same, and the profiles are extracted at the same location in different acquisitions, the extracted diffusion length markedly decreases when reducing the pixel size. The diffusion length values saturate around a certain final value (in this case around 130 nm) when the pixel size is small enough. The diffusion length is a material parameter that should not be influenced at all by the scale of investigation. As shown by the graph, and more in general for other cases that we cross-checked, extracted values from the fits resulted overall constant once the pixel size was roughly 10 times smaller than the diffusion lengths themselves.

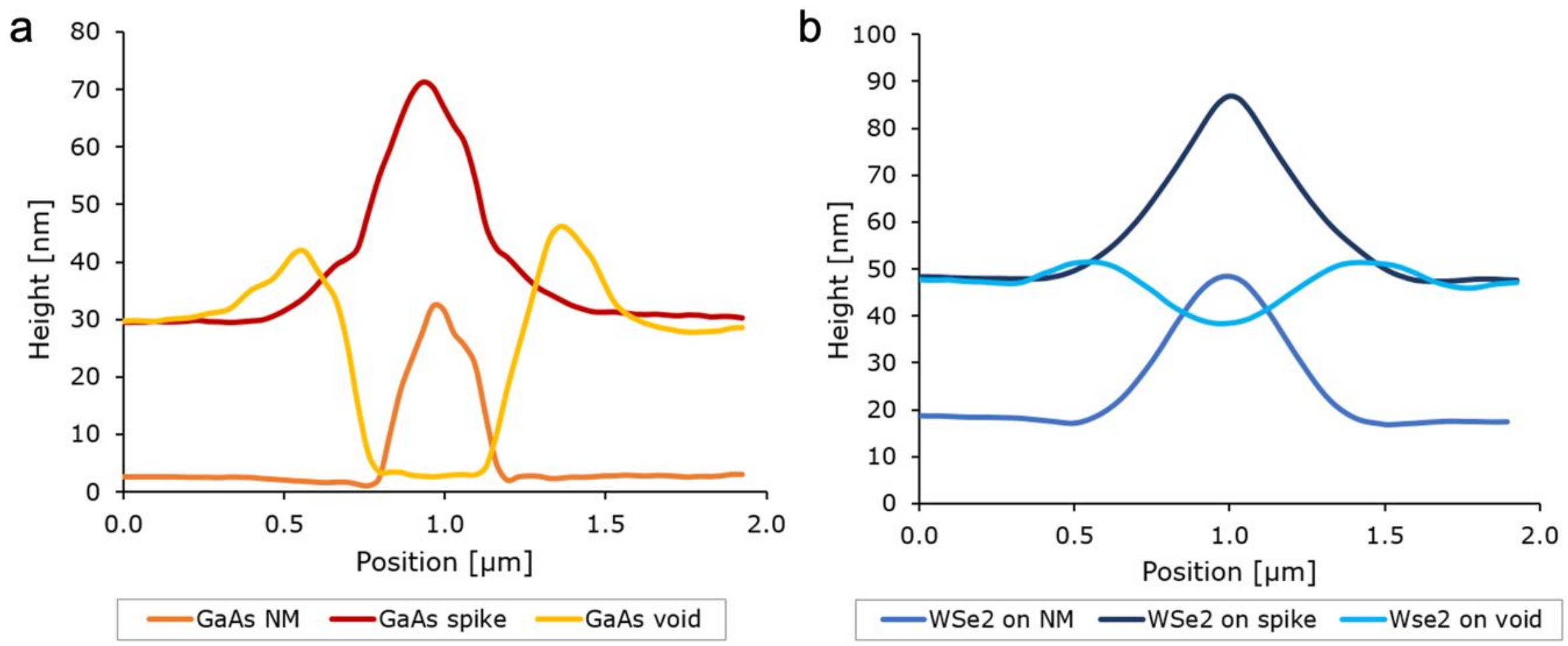

Supplementary Figure 17*: Morphological characterization of NXs and WSe_2 on NXs. (a) AFM height profiles of the NM constituting the branch of a NX, of the spike at the center of the NX, and of the void between different NXs; (b) AFM height profiles of the WSe_2 flake in its central region of constant thickness on top of the NM, on top of the spike at the center of the NXs, and floating on top of the central void between NXs.*

The NXs network consists of parallel and perpendicular linear nanostructures partially intersected. Intersections result in the growth of localized spikes with average base diameter of 850 nm (red curve) while disconnected areas appear as 530 nm wide voids reaching down the SiO_2 mask (yellow curve), as shown by the profiles in Supplementary Figure 17a. Similar profiles for the flake on top of NXs are presented in Supplementary Figure 17b, highlighting conformality of the 2D material on top of the spikes (dark blue curve) and evident suspension at disconnected regions in between NXs (light blue curve).

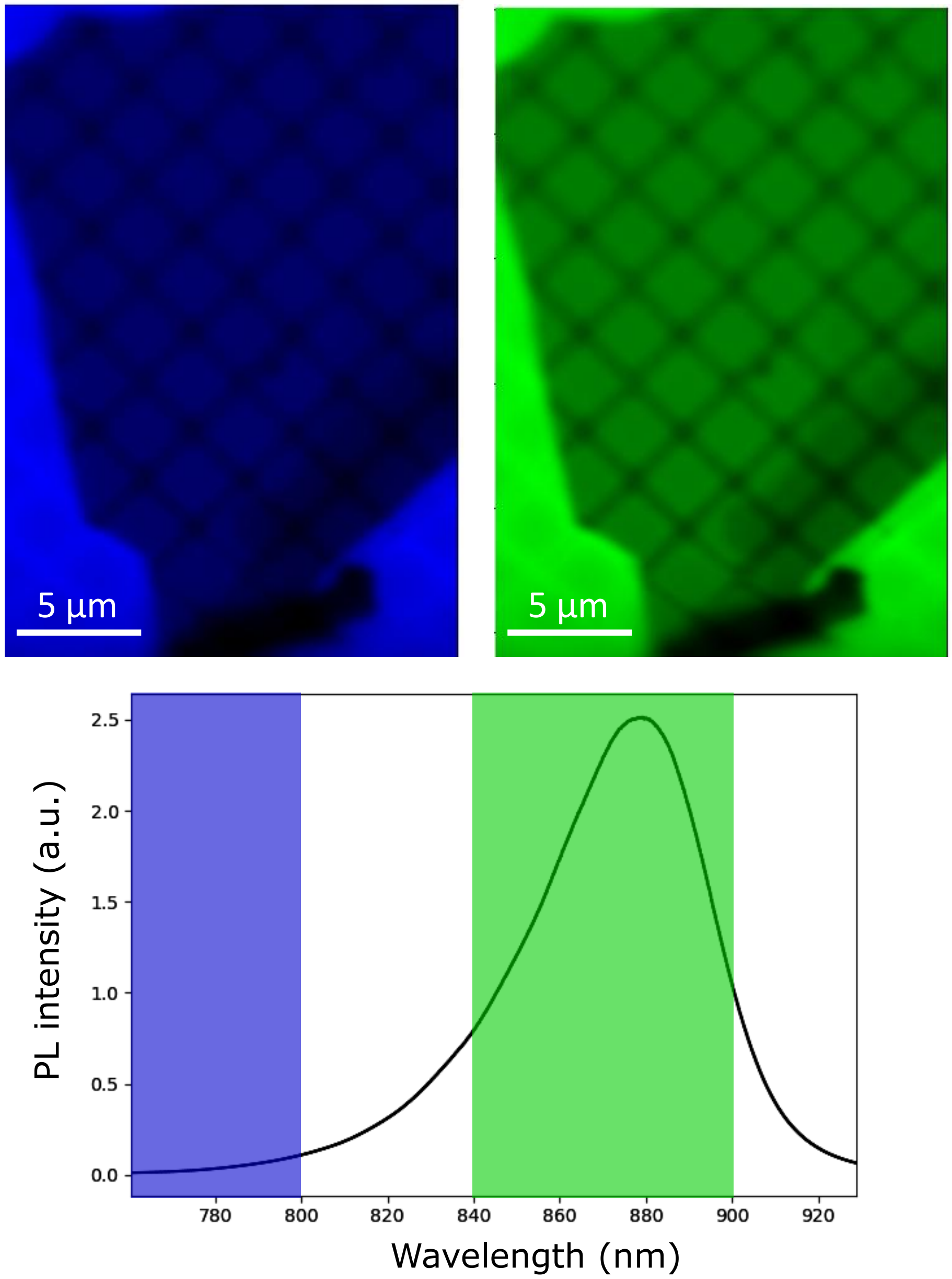

Supplementary Figure 18*: Hyperspectral maps of a WSe_2 flake on GaAs NXs integrated in the WSe2 range, between 760 nm and 800 nm (left) and the GaAs range, between 840 nm and 900 nm (right), as shown in the spectrum cumulated over all pixels of the map, shown below.*

The absence of bright emission areas from the PL hyperspectral map integrated in the WSe_2 wavelength range (760 nm to 800 nm) on Supplementary Figure 18 (top left), as well as the similarity with the map integrated in the GaAs range (840 nm to 900 nm) on Supplementary Figure 18 (top right) confirms the absence of a WSe_2 monolayer. The PL contribution in the WSe_2 wavelength range comes entirely from the tale of the GaAs emission.

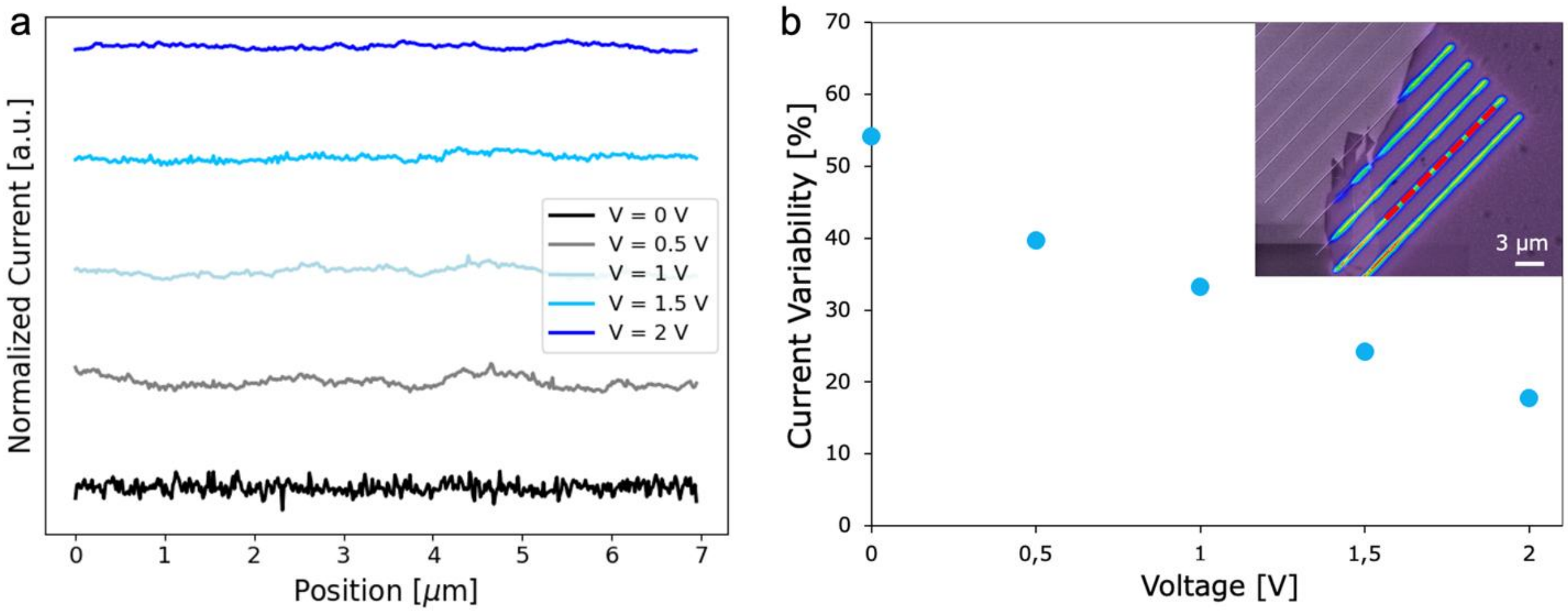

Supplementary Figure 19: *Current variability at different bias. (a) Normalized current profiles along the interface between WSe_2 and GaAs NMs at different biases, showing increased reproducibility when increasing external voltage; (b) Percentage current variability decreasing with external bias at the WSe_2/GaAs NMs interface, proving signal stabilization with increasing external voltage. Top right inset shows the experimental EBIC map from which profiles are extracted along the red dashed curve at different voltages. EBIC maps have been acquired under electron beam with 3 keV acceleration voltage and ~ 0.4 nA probe current)*

Current values differ under various applied voltage, increasing significantly at larger bias. This trend is shown in Supplementary Figure 19a, for current profiles extracted longitudinally on top of the 2D/1D heterojunction, as the red dashed curve of the inset of Supplementary Figure 19b. At low voltages, EBIC signal-to-noise ratio decreases, causing current fluctuations that drop from more than 50% at 0.0V to less than 20% at 2.0V, as presented by the plot in Supplementary Figure 19b.

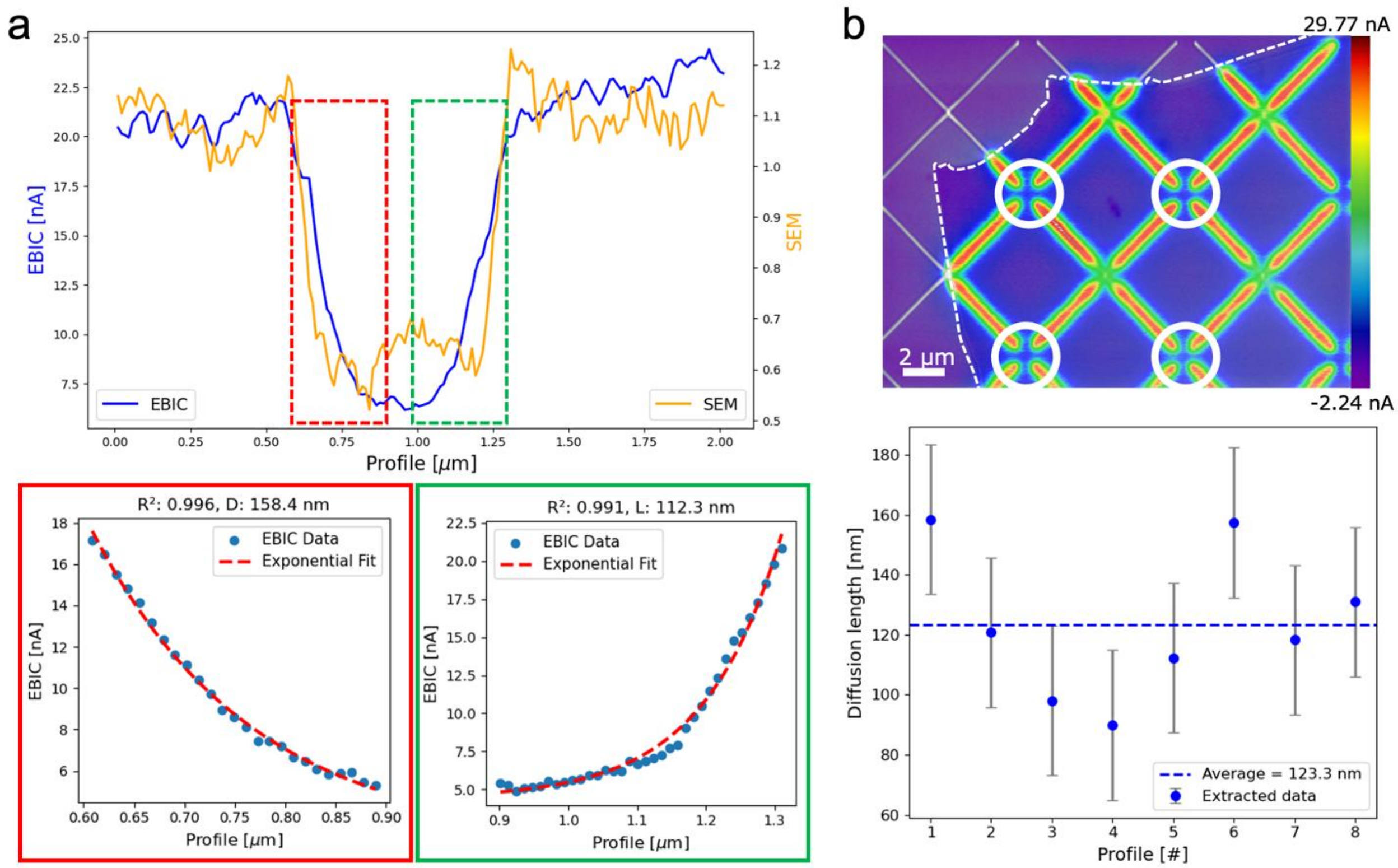

***Supplementary Figure 20**: Diffusion length analysis for NXs. (a) Superimposed SEM and EBIC signal in correspondence of the void between NXs. Overlaid dashed rectangles highlight regions for the fitted portions of the EBIC signal, as shown in the respective insets below the plot. (b) Extracted diffusion length values (in the bottom plot) for different profiles traced at the locations indicated by white circles in the EBIC map on top. EBIC maps have been acquired under electron beam with 3 keV acceleration voltage and ~ 0.4 nA probe current)*

The diffusion length analysis for WSe_2 on GaAs NXs is performed analogously to the previous integrations. Here, void regions in between NXs where no WSe_2/GaAs interface is present allow for hole diffusion length estimation in WSe_2. Supplementary Figure 20a shows superimposed SEM and EBIC signals where fitting regions are highlighted by dashed curves in red and green. Consistently, the two subplots at the bottom show the fitted exponentials with highly reliable fit quality. Diffusion length values for this heterointegration are averaged among various profiles obtained from multiple void locations, when possible. Supplementary Figure 20b shows the magnified EBIC map where such locations are highlighted by white circles. The plot at the bottom shows extracted diffusion length values from different profiles, where the dashed line the average value and the error bars represent the standard deviation. Discrepancies in extracted diffusion length values could be ascribed to different morphological relaxations of the flake in the void regions.

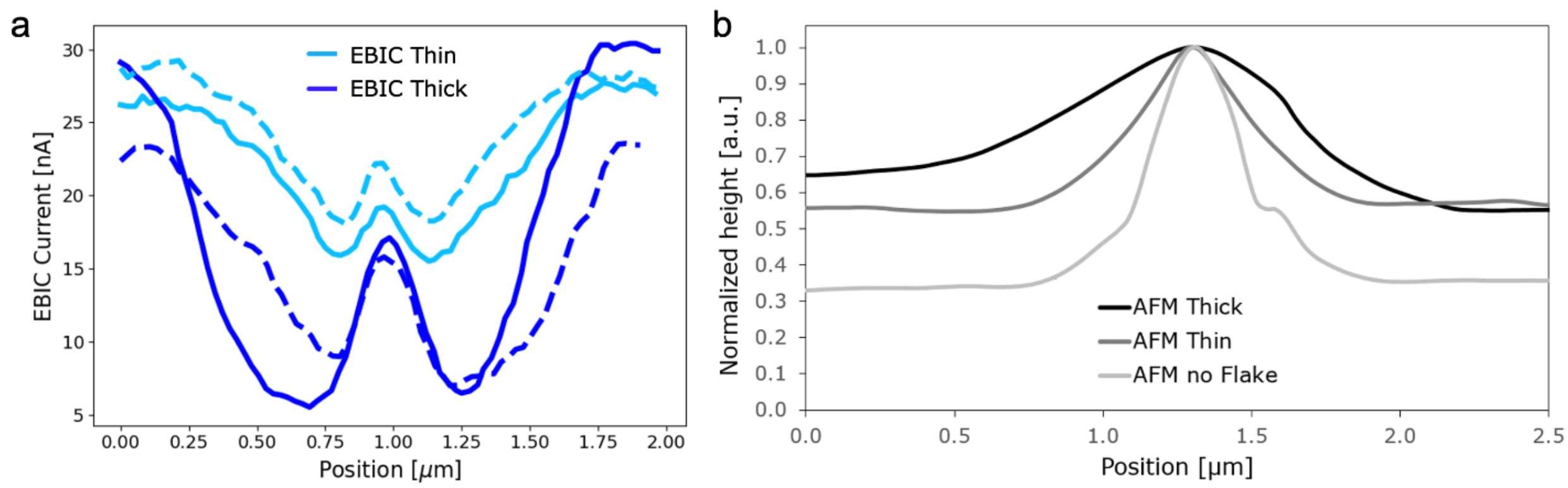

Supplementary Figure 21*: Current and morphological behavior at the spikes. (a) EBIC current profiles on the thin (19 nm) and thick (37 nm) homogeneous portions of the WSe_2 flake on top of the central spike in the NXs. Solid curves refer to the <001> direction, while dashed ones to the perpendicular <010> one. (b) AFM normalized heights of the bare spike, thin flake and thick flake on top, showing different conformality depending on the thickness. EBIC maps have been acquired under electron beam with 3 keV acceleration voltage and ~ 0.4 nA probe current)*

The presence of a central area of contact is expected to lead to localized current collection, as indeed shown in Supplementary Figure 21a. EBIC profiles at the spike location reveal the presence of current peaks at the central position as expected. The current profiles extracted along perpendicular directions (filled and dashed curves of same color) on both flake thicknesses show marked reproducibility. Taller structures could improve the in-plane confinement but would also impact the strain distribution and could increase the chance of destructive events such as piercing [4]. Thicker regions instead appear to result in stronger confinement (blue curve of Supplementary Figure 21a), most likely due to a reduced lateral adhesion of stiffer flakes. AFM profiles in Supplementary Figure 21b indeed show different conformality at different thicknesses.

References

1. Papatryfonos, K. *et al*. Refractive indices of MBE-grown $Al_xGa_{(1-x)}As$ ternary alloys in the transparent wavelength region. *AIP Advances* **11**, 025327 (2021).
2. Morgan, N. *et al.* From Layer-by-Layer Growth to Nanoridge Formation: Selective Area Epitaxy of GaAs by MOVPE. *Crystal Growth & Design* **23**, 5083–5092 (2023).
3. Dede, D. *et al.* Selective area epitaxy of GaAs: the unintuitive role of feature size and pitch. *Nanotechnology* **33**, 485604 (2022).
4. Balgarkashi, A. *et al*. Spatial modulation of vibrational and luminescence properties of monolayer MoS_2 using a GaAs nanowire array. *IEEE Journal of Quantum Electronics* **58**(4), 1–8 (2022).
